# Nowcasting COVID‐19 deaths in England by age and region

**DOI:** 10.1111/rssc.12576

**Published:** 2022-06-15

**Authors:** Shaun R. Seaman, Pantelis Samartsidis, Meaghan Kall, Daniela De Angelis

**Affiliations:** ^1^ MRC Biostatistics Unit University of Cambridge Cambridge Cambridgeshire UK; ^2^ COVID‐19 National Epidemiology Cell UK Health Security Agency London UK; ^3^ Statistics, Modelling and Economics Department, Data, Analytics and Surveillance UK Health Security Agency London UK

**Keywords:** epidemic monitoring, generalised Dirichlet, reporting delay, right‐truncation

## Abstract

Understanding the trajectory of the daily number of COVID‐19 deaths is essential to decisions on how to respond to the pandemic, but estimating this trajectory is complicated by the delay between deaths occurring and being reported. In England the delay is typically several days, but it can be weeks. This causes considerable uncertainty about how many deaths occurred in recent days. Here we estimate the deaths per day in five age strata within seven English regions, using a Bayesian model that accounts for reporting‐day effects and longer‐term changes in the delay distribution. We show how the model can be computationally efficiently fitted when the delay distribution is the same in multiple strata, for example, over a wide range of ages.

## 1 INTRODUCTION

The first COVID‐19 case in the United Kingdom was reported on 30 January 2020, and the first death was announced on 6 March. Underlying the Government's response to the COVID‐19 epidemic is surveillance information on a number of epidemiological indicators. In particular, trends in the number of deaths have been crucial to monitoring the burden of COVID‐19 as well as to informing models reconstructing and predicting the pandemic. Information on these trends is affected by delayed reporting. While the number of deaths in people with laboratory‐confirmed COVID‐19 infection peaked on 9 April, the reports of such deaths peaked 13 days later, on 22 April. This is because deaths are rarely reported to the authorities on the day they occur, and some deaths take weeks to be reported. This reporting delay makes it more difficult to establish the true pattern of the numbers of deaths occurring over time and to identify quickly any increase in recent days. The process of estimating the number of deaths that occurred on each day from the deaths so far reported is called ‘nowcasting’. Here we nowcast the numbers of deaths in people with COVID‐19, for all of England and separately for seven English regions, broken down into five age strata: 0–44, 45–54, 55–64, 65–74 and ≥75 years.

We use data on deaths in people with a laboratory‐confirmed COVID‐19 diagnosis reported to Public Health England (PHE) from three sources. Reports from National Health Service England (NHSE) include deaths in hospitals notified by NHS trusts through the COVID‐19 Patient Notification System. Demographics Batch Service (DBS) reports result from the daily tracing of COVID‐19 positive tests reported to PHE through the Second Generation Surveillance System in the NHS Spine to identify individuals who have died in the previous 24 h in any setting. Health Protection Team (HPT) reports include deaths notified by PHE HPT during outbreak management, primarily in non‐hospital settings (e.g. care homes). The three data sources are not independent; duplications are identified daily and only the earliest report date are considered.

**FIGURE 1:**
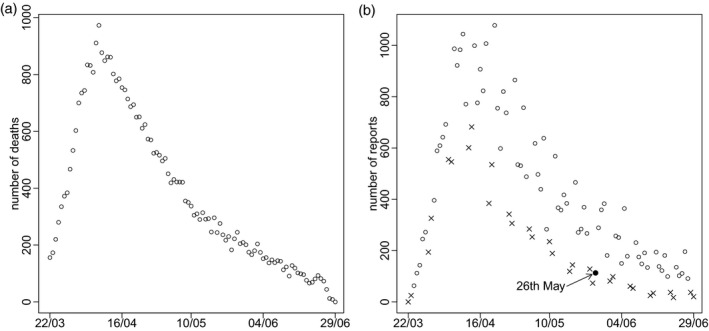
Numbers of reported deaths by date of (a) death and (b) report (cross indicates Sunday or Monday).

In this article we shall illustrate our method using the data available on 29 June 2020. There were 37529 deaths that occurred after 22 March (the day after the first reports from DBS became available) and were reported by 29 June. Figure 1a shows the number of deaths that occurred on each day. The effect of reporting delay is particularly evident in the last 3 days: while there were 50–100 deaths per day in the week up to 25 June, the numbers for 27, 28 and 29 June were 12, 9 and 0. Figure 1b shows the number of deaths reported on each day. It tends to be lower on Sundays and Mondays, probably due to some staff not working at the weekend. This is particularly so in more recent weeks. Also, the number of reports over a single Tuesday‐to‐Saturday period varies considerably from day to day. It is particularly low, for example, on Tuesday 26 May.

**FIGURE 2:**
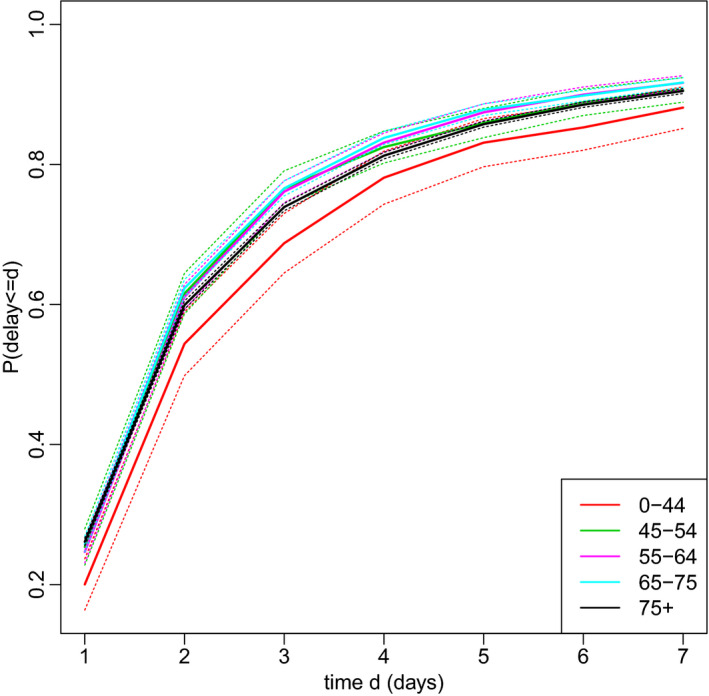
Estimated reporting delay distribution by age. Dotted lines represent 95% confidence intervals. For ease of viewing, only the first seven days are shown.

Figure 2 shows a simple estimate (see Brookmeyer and Liao (1990) for method) of the reporting delay distribution by age stratum, calculated assuming the delay does not depend on date of death. This distribution is conditional on the delay being at most 42 days. Forty‐two was chosen because only 61 of the 37529 reported deaths had delays longer than 6 weeks. We see that delays tend to be slightly longer in the ≥75 years stratum and slightly longer still in the 0–44 stratum.

A simple way to adjust for reporting delay is to use the estimates in Figure 2 to weight up the deaths reported so far. For example, in the ≥75 stratum, nine deaths occurred on 27 June and were reported by 29 June. The estimated proportion of deaths reported within 2 days in this strata is 0.60. So, the actual number of deaths can be estimated as 9/0.60 = 15. However, this estimate does not account for dependence of the delay on date of death. Delays may length or shorten over calendar time and may depend (as Figure 1b suggests) on the day of the week (henceforth, ‘weekday’) on which the death occurred. Also, while the number of deaths occurring on each day is likely to change smoothly over time, this estimate does not use information about the numbers of deaths that occurred on preceding and succeeding days, and so can be very different from the estimates for those days. In addition, simple confidence interval calculations (Brookmeyer & Liao, 1990) underestimate uncertainty, unless the delay distribution really is the same for all dates of death.

Accounting for reporting delays is complicated by weekday reporting effects, changes over calendar time in the delay distribution, and excess variability (overdispersion) in the daily numbers of reports. Stoner and Economou (2020b) reviewed recent literature on delayed reporting, and elaborated a Bayesian model earlier proposed by Höhle and an der Heiden (2014). Stoner and Economou (2019) further developed this model into a hierarchical model for multiple strata (e.g. regions), and Stoner and Economou (2020a) used this, together with reports from NHSE, to nowcast COVID‐19 deaths over the period 2 April to 5 May in each of seven English regions.

The general approach of Stoner and Economou (henceforth, S&E) has several advantages. It flexibly models weekday reporting effects and longer‐term changes in the delay distribution. By allowing for overdispersion in the daily numbers of reports that cannot be explained by weekday reporting effects, it prevents any such overdispersion from causing excessive variance in the estimates of the daily numbers of deaths (S&E, 2020b). By modelling the expected number of deaths per day in each stratum as a smooth function of time, it allows the estimate of number of deaths that occurred on 1 day to borrow information about the numbers of deaths on the preceding and succeeding days. Also, information about the delay distribution and pattern of the epidemic in one stratum informs those quantities in the other strata. Uncertainty can be quantified by posterior credible intervals (CIs).

Here we build on S&E's hierarchical model to nowcast in each region, with stratification by age group. Our model improves on theirs in a number of ways. First, we model weekday reporting effects more realistically. Second, we account for correlation between the numbers of reports made on the same day about individuals who died on different days. If, for example, the data suggest that an unusually low proportion of those deaths that occurred between 2 and 7 days ago have been reported today (compared to what would typical for this day of the week), then it is likely that few of the deaths that occurred yesterday and today will have yet been reported. Third, we describe a computationally efficient way to constrain the delay distribution to be the same in multiple strata. This is particularly useful if the distribution can be expected to be the same across a wide range of ages. We also illustrate how to use the model to calculate a posterior probability that the number of deaths has been rising in recent days.

## 2 MATERIALS AND METHODS

Denote the first day for which data on reporting delays will be used as day 0 and the most recent day for which data are available as day *T*. We restrict attention to deaths that are reported within *D* days. We use *D* = 42 in Section 3. Let $D_{t}$ denote the longest delay that we could have observed in individuals who died on day *t* (*t* = 0, …, *T*). So, $D_{t}=D$ if *t* ≤ *T* − *D* and $D_{t}=T-t$ otherwise. Let $Z_{\mathrm{std}}$ denote the number of deaths that occurred on day *t* in age stratum *s* (*s* = 1, …, *S*) and are reported with a delay of *d* days (*d* = 0, …, *D*). If $d\leq D_{t}$, $Z_{\mathrm{std}}$ is observed; otherwise it is unobserved. In Section 3 we used *S* = 5 strata: 0–44 (*s* = 1), 45–54 (*s* = 2), 55–64 (*s* = 3), 65–74 (*s* = 4) and ≥75 (*s* = 5) years. Let $W_{\mathrm{st}}=\sum_{j=0}^{D_{t}}Z_{\mathrm{stj}}$ be the (observed) number of deaths reported in stratum *s* by time *T* among individuals who died on day *t*. Let $Y_{\mathrm{st}}=\sum_{d=0}^{D}Z_{\mathrm{std}}$ be the number of deaths that occurred on day *t* in age stratum *s*. For *t* ≤ *T* − *D*, $Y_{\mathrm{st}}=W_{\mathrm{st}}$ is observed. For *t* > *T* − *D*, $Y_{\mathrm{st}}$ is unobserved and we seek to estimate it.

We model the data in a Bayesian framework, specifying three submodels: one for the marginal distribution of the number of deaths, $Y_{\mathrm{st}}$, occurring on each day in each stratum (Section 2.1); one for the conditional distribution of the observed numbers of reports, $Z_{\mathrm{std}}$, given the numbers of deaths and the delay distribution (Section 2.2); and one for the delay distribution (Section 2.3). Figure S1 shows a directed acyclic graph for the model (figures prefixed by ‘S’ are in the Supplementary Material). We fit this model using a Markov chain Monte Carlo (MCMC) algorithm.

### 2.1 Model for numbers of deaths

For *t* > *T* − *D*, $Y_{\mathrm{st}}$ is unobserved. For stratum *s* = 1, …, *S* and time *t* = *T* − *D* + 1, …, *T*, assume

(1) $$Y_{\mathrm{st}}\;|\;\lambda_{\mathrm{st}},\theta ∼\text{NegativeBinomial}\;\left(\text{mean}=\lambda_{\mathrm{st}},\text{variance}=\lambda_{\mathrm{st}}\left(1+\theta \right)\right),$$

where $\lambda_{s,T-D+1},\ldots ,\lambda_{\mathrm{sT}}$ and overdispersion *θ* (≥0) are unknown parameters. If *θ* = 0, then $Y_{\mathrm{st}}|\lambda_{\mathrm{st}},\theta ∼\text{Poisson}\left(\lambda_{\mathrm{st}}\right)$. We shall assume $\lambda_{\mathrm{st}}$ changes smoothly over time *t*.

So far we have not used the observed data on the numbers of deaths $Y_{\mathrm{st}}$ that occurred in the period before day *T* − *D* + 1. Such data may be useful, as they indicate whether the number of deaths per day was rising or falling over that period. It could reasonably be expected that the trajectory of deaths per day in the period shortly after day *T* − *D* + 1 would be a continuation of the trajectory immediately before that day. To exploit this information, we shall assume expression (1) also holds for $t=T_{0},\ldots ,T-D$, where $T_{0}$ is some time between 0 and *T* − *D*. We chose $T_{0}=T-D-13$, thus using two weeks of data.

We follow S&E in using hierarchical restrictive penalised cubic splines to model the assumption that $\lambda_{\mathrm{st}}$ changes smoothly over time *t* and may follow somewhat similar trajectories in different strata *s*. More precisely, $\log\lambda_{\mathrm{st}}$ ($t=T_{0},\ldots ,T$) is taken to be the sum of an unknown stratum‐specific intercept term $\iota_{s}$ and two restricted cubic splines, one common to all strata and one specific to stratum *s*. That is, $\log\lambda_{\mathrm{st}}=\iota_{s}+g_{\mathrm{st}}^{M}$, where $g_{\mathrm{st}}^{M}=x_{t-T_{0}+1}^{⊤}\left(\tilde{\mu }+\mu_{s}\right)$ (superscript ‘M’ stands for ‘mortality’). Here, $\tilde{\mu }\;|\;\tilde{\tau }∼\text{Normal}\left(0,V{\tilde{\tau }}^{-1}\right)$, $\mu_{s}\;|\;\tau_{s}∼\text{Normal}\left(0,V\tau_{s}^{-1}\right)$, ${x_{j}}^{⊤}$ ($j=1,\ldots ,T-T_{0}+1$) is the *j*th row of the design matrix *X* for the splines, and *V* is the corresponding variance matrix (Wood, 2016, 2017). The unknown parameters $\tilde{\tau }$ and $\tau_{s}$ determine the smoothness of the splines. If $\tau_{s}=\infty$ for all *s*, then $\lambda_{\mathrm{st}}\propto \lambda_{s^{\prime }t}$ for all *s* and $s^{\prime }$, that is, the trends in mortality are the same in all strata.

### 2.2 Model for observed number of reports

For stratum *s* = 1, …, *S* and time *t* = 0, …, *T*, assume

(2) $$\left(Z_{st0},\ldots ,Z_{\mathrm{stD}}\right)\;|\;Y_{\mathrm{st}},\left(P_{st0},\ldots ,P_{\mathrm{stD}}\right)∼\text{Multinomial}\left(Y_{\mathrm{st}},\;\left(P_{st0},\ldots ,P_{\mathrm{stD}}\right)\right),$$

where $P_{\mathrm{std}}$ is the unknown probability that an individual in stratum *s* who died on day *t* is reported with a delay of *d* days.

We shall allow $P_{\mathrm{std}}$ to depend on *t* through both a smooth function of calendar time and weekday reporting effects. Even after accounting for these systematic effects, $Z_{\mathrm{std}}$ may be overdispersed compared to a multinomial distribution. As S&E (2020a) showed, failure to allow for such overdispersion can lead to overdispersion in $Y_{\mathrm{st}}$ being inferred (i.e. large estimated *θ*), and thus to unnecessarily imprecise nowcasts. So, to allow for overdispersion in $Z_{\mathrm{std}}$, assume, for each *s* and *t*, that $(P_{st0},\ldots ,P_{\mathrm{stD}})∼\text{GD}\left(\alpha_{\mathrm{st}},\beta_{\mathrm{st}}\right)$, where GD is the generalised Dirichlet distribution (Connor & Mosimann, 1975) and $\alpha_{\mathrm{st}}=\left(\alpha_{st0},\ldots ,\alpha_{st,D-1}\right)$ and $\beta_{\mathrm{st}}=\left(\beta_{st0},\ldots ,\beta_{st,D-1}\right)$ are unknown parameters, which will be modelled as functions of *s*, *t* and *d*. The GD distribution is a flexible generalisation of the Dirichlet distribution; it allows, for example, positive correlation between $P_{\mathrm{std}}$ and $P_{std^{\prime }}$ for $d\neq d^{\prime }$, unlike the Dirichlet distribution.

The marginal distribution of $\left(Z_{st0},\ldots ,Z_{\mathrm{stD}}\right)$ (given $Y_{\mathrm{st}}$, $\alpha_{\mathrm{st}}$ and $\beta_{\mathrm{st}}$) obtained by integrating out $\left(P_{st0},\ldots ,P_{\mathrm{stD}}\right)$ is the generalised‐Dirichlet‐multinomial (GDM) distribution (Connor & Mosimann, 1975). This GDM distribution can be factorised as the product over *d* = 0, …, *D* − 1 of conditional distributions $Z_{\mathrm{std}}|Z_{st0},\ldots ,Z_{st,d-1},Y_{\mathrm{st}},\alpha_{\mathrm{st}},\beta_{\mathrm{st}}∼\text{Beta‐Binomial}(\alpha_{\mathrm{std}},\beta_{\mathrm{std}};Y_{\mathrm{st}}-\sum_{j=0}^{d-1}Z_{\mathrm{stj}}).$ This beta‐binomial distribution has probability mass function

(3) $$\frac{\left(Y_{\mathrm{st}}-\sum_{j=0}^{d-1}Z_{\mathrm{stj}}\right)!}{\left(Y_{\mathrm{st}}-\sum_{j=0}^{d}Z_{\mathrm{stj}}\right)!Z_{\mathrm{std}}!}\times \frac{B\left(Z_{\mathrm{std}}+\alpha_{\mathrm{std}},\;Y_{\mathrm{st}}-\sum_{j=0}^{d}Z_{\mathrm{stj}}+\beta_{\mathrm{std}}\right)}{B(\alpha_{\mathrm{std}},\beta_{\mathrm{std}})},$$

where *B*(.,.) denotes the beta function (a ratio of gamma functions). Since $Z_{\mathrm{std}}$ is unobserved for $d>D_{t}$, we only need include the likelihood contribution of $\left(Z_{st0},\ldots ,Z_{stD_{t}}\right)$, that is, the product of expression (3) over $d=0,\ldots ,\min(D_{t},D-1)$. By integrating out the unknown variables $Z_{st,D_{t}+1},\ldots ,Z_{\mathrm{stD}}$ in this way, the MCMC algorithm used to fit the model is simplified compared to the algorithm used by S&E, the computational load is reduced, and the mixing of the Markov chain should be improved. Note the $Z_{\mathrm{std}}!$ term in expression (3) can be ignored when calculating full‐conditional distributions, because it does not depend on any unobserved variable.

### 2.3 Model for the delay distribution

We reparameterise the GD distribution in terms of parameters $\left(v_{st0},\ldots ,v_{st,D-1}\right)$ and $\left(\phi_{st0},\ldots ,\phi_{st,D-1}\right)$, where $v_{\mathrm{std}}=\alpha_{\mathrm{std}}/\left(\alpha_{\mathrm{std}}+\beta_{\mathrm{std}}\right)$ and $\phi_{\mathrm{std}}=\alpha_{\mathrm{std}}+\beta_{\mathrm{std}}$. Now, $v_{\mathrm{std}}=E\left(P_{\mathrm{std}}/\sum_{j=d}^{D}P_{\mathrm{stj}}\right)$ is the expectation of the hazard of a reporting delay of *d* days for an individual in stratum *s* who died on day *t*, and the variance of this hazard is $\mathrm{Var}\left(P_{\mathrm{std}}/\sum_{j=d}^{D}P_{\mathrm{stj}}\right)=v_{\mathrm{std}}\left(1-v_{\mathrm{std}}\right)/\left(\phi_{\mathrm{std}}+1\right)$ (Connor & Mosimann, 1975). Note that as $\phi_{\mathrm{std}}\to \infty$, this variance tends to zero, which makes the GDM distribution of $(Z_{st0},\ldots ,Z_{\mathrm{stD}})|Y_{\mathrm{st}}$ reduce to the multinomial distribution of expression (2) with $P_{\mathrm{std}}=v_{\mathrm{std}}\prod_{j=0}^{d-1}\left(1-v_{\mathrm{stj}}\right)$. Correspondingly, the beta‐binomial distribution of expression (3) reduces to a binomial distribution. Thus, the $\phi_{\mathrm{std}}$ parameters describe the overdispersion of the numbers of reports relative to a multinomial distribution.

It is easy to show that $v_{\mathrm{std}}$ can be written as $v_{\mathrm{std}}=\left(q_{\mathrm{std}}-q_{st,d-1}\right)/\left(1-q_{st,d-1}\right),$ where $q_{\mathrm{std}}=E\left(\sum_{j=0}^{d}P_{\mathrm{stj}}\right)$ is the expectation of the probability that the delay is at most *d* days.

#### 2.3.1 Weekday effects

We model weekday reporting effects by assuming for *d* = 0, …, *D* − 1 that

(4) $$\begin{array}{cc} \text{logit}\left(v_{\mathrm{std}}\right)\equiv \text{logit}\left(\frac{q_{\mathrm{std}}-q_{st,d-1}}{1-q_{st,d-1}}\right) & =\text{logit}\left(\frac{q_{\mathrm{std}}^{*}-q_{st,d-1}^{*}}{1-q_{st,d-1}^{*}}\right)+\sum_{j=1}^{7}\eta_{\mathrm{sj}}\;I\left\{\text{Day}\left(t+d\right)=j\right\}\\ & \;+g_{\mathrm{st}}^{W}\;I\left\{\text{Day}\left(t+d\right)=1\;\text{or}\;7\right\}. \end{array}$$

Here, *I*(.) is the indicator function, and Day(*t* + *d*) = 1 if day *t* + *d* is Monday, Day(*t* + *d*) = 2 if Tuesday, and so on. $q_{\mathrm{std}}^{*}$ and $\eta_{s1},\eta_{s2},\eta_{s3},\eta_{s4},\eta_{s5},\eta_{s7}$ are unknown parameters; we constrain $\eta_{s6}=0$ for identifiability (so, Saturday is baseline). $g_{\mathrm{st}}^{W}$ is a smooth function of *t*, which describes how the Sunday and Monday effects change over calendar time (superscript ‘W’ stands for ‘weekend’). As with $g_{\mathrm{st}}^{M}$, we assume $g_{\mathrm{st}}^{W}$ is the sum of two restricted cubic splines, one common to all strata and one specific to stratum *s*. Equation (4) means that, given the delay is at least *d* days, the odds of a death on day *t* being reported with delay *d* is $\exp\{\eta_{\mathrm{sj}}+g_{\mathrm{st}}^{W}I\left(\;j=1\;\text{or}\;7\right)\}$ times greater when day *t* + *d* is weekday *j* than it would be if day *t* + *d* were Saturday.

#### 2.3.2 Calendar‐time effects

To model calendar‐time effects, we assume for *d* = 0, …, *D* − 1 that

(5) $$\text{probit}\left(q_{\mathrm{std}}^{*}\right)=\text{probit}\left(q_{\mathrm{sd}}^{†}\right)+g_{\mathrm{st}}^{C},$$

where $q_{\mathrm{sd}}^{†}$ is an unknown parameter and $g_{\mathrm{st}}^{C}$ is a smooth function of *t*. As with $g_{\mathrm{st}}^{M}$ and $g_{\mathrm{st}}^{W}$, we assume $g_{\mathrm{st}}^{C}$ is the sum of two restricted cubic splines. To ensure $0\leq q_{s0}^{†}\leq q_{s1}^{†}\leq \cdots \leq q_{s,D-1}^{†}\leq 1$, and hence $(q_{\mathrm{std}}^{*}-q_{st,d-1}^{*})/\left(1-q_{st,d-1}^{*}\right)$ in Equation (4) lies between 0 and 1, we reparameterise $q_{\mathrm{sd}}^{†}$ as $q_{\mathrm{sd}}^{†}=1-\prod_{j=0}^{d}\left(1-\psi_{\mathrm{sj}}\right),$ where $0\leq \psi_{\mathrm{sd}}\leq 1$ (*d* = 0, …, *D* − 1). Note that if $\eta_{1}=\cdots =\eta_{7}=g_{\mathrm{st}}^{W}=g_{\mathrm{st}}^{C}=0$, then $\psi_{\mathrm{sd}}=v_{\mathrm{std}}$ and $q_{\mathrm{sd}}^{†}=q_{\mathrm{std}}$. In Section 5, we contrast our approach to modelling weekday and calendar‐time reporting effects with S&E's approach.

#### 2.3.3 Reporting‐day random effects

Parameters $\eta_{\mathrm{sj}}$ and $g_{\mathrm{st}}^{W}$ are reporting‐day effects that allow deaths to be more likely to be reported on certain days of the week. In addition to these systematic weekday effects, there may be non‐systematic reporting‐day effects. That is, there may be individual days on which the numbers of reports are unpredictably higher or lower than would be expected given the weekday and the number of deaths that have occurred in recent days. Figure 1b suggests 26 May is an example. These non‐systematic reporting‐day effects would alter the expected hazard $v_{\mathrm{std}}$ on day *t* + *d* for delays of all lengths *d*, and hence induce correlations between $P_{\mathrm{std}},P_{s,t+1,d-1},P_{s,t+2,d-2},P_{s,t+3,d-3},\ldots$. Such correlations are not reflected by the GD distribution introduced in Section 2.1, which instead induces correlations between $P_{st0},P_{st1},P_{st2},P_{st3},\ldots$. To allow for these non‐systematic effects, we introduce i.i.d. random effects $\delta_{0},\ldots ,\delta_{T}∼\text{Normal}\left(0,\omega^{2}\right)$ (where *ω* is an unknown parameter) and add $\delta_{t+d}$ to the right‐hand side of Equation (4).

#### 2.3.4 Strata with a common delay distribution

We now consider two alternative model adaptations to constrain the delay distributions to be the same in some set 𝒮 of strata. The first takes $\eta_{\mathrm{sj}}$, $\psi_{\mathrm{sd}}$, $g_{\mathrm{st}}^{W}$, $g_{\mathrm{st}}^{C}$ and $\phi_{\mathrm{std}}$ to be equal for all s∈𝒮. Denote the common values by a dot, so $\eta_{\mathrm{sj}}=\eta_{•j}$, $\psi_{\mathrm{sd}}=\psi_{•d}$, $g_{\mathrm{st}}=g_{•t}^{W}$, $g_{\mathrm{st}}=g_{•t}^{C}$ and $\phi_{\mathrm{std}}=\phi_{•td}$. Then $v_{\mathrm{std}}=v_{•td}$, and $\left(P_{st0},\ldots ,P_{\mathrm{stD}}\right)$ have the same GD distribution for strata s∈𝒮.

The second adapted model instead assumes $P_{\mathrm{std}}=P_{•td}$ for all s∈𝒮, that is, the delay distributions $\left(P_{st0},\ldots ,P_{\mathrm{stD}}\right)$ are exactly equal for s∈𝒮. Let $Z_{+td}=\sum_{s\in 𝒮}Z_{\mathrm{std}}$, $Y_{+t}=\sum_{s\in 𝒮}Y_{\mathrm{st}}$ and $W_{+t}=\sum_{s\in 𝒮}W_{\mathrm{st}}$. We show in the Supplementary Material that when $P_{\mathrm{std}}=P_{•td}$,

$$\begin{array}{cc} & p(\left\{Z_{st0},\ldots ,Z_{\mathrm{stD}}:s\in 𝒮\right\}|\left\{Y_{\mathrm{st}}:s\in 𝒮\right\},\alpha_{•t},\beta_{•t})\\ & \;\propto \prod_{d=0}^{\min(D_{t},D-1)}p\left(Z_{+td}\;|\;Z_{+t0},\ldots ,Z_{+t,d-1},Y_{+t},\alpha_{•t},\beta_{•t}\right)\times p\left(\left\{W_{\mathrm{st}}:s\in 𝒮\right\}|\left\{Y_{\mathrm{st}}:s\in 𝒮\right\},W_{+t}\right). \end{array}$$

Assuming $\left(P_{•t0},\ldots ,P_{•tD}\right)$ is drawn from a GD distribution, we have (as in expression (3)),

(6) $$\begin{array}{cc} & p(Z_{+td}\;|\;Z_{+t0},\ldots ,Z_{+t,d-1},Y_{+t},\alpha_{•td},\beta_{•td})\\ & \;=\frac{\left(Y_{+t}-\sum_{j=0}^{d-1}Z_{+tj}\right)!}{\left(Y_{+t}-\sum_{j=0}^{d}Z_{+tj}\right)!\;Z_{+td}!}\times \frac{B\left(Z_{+td}+\alpha_{•td},\;Y_{•t}-\sum_{j=0}^{d}Z_{+tj}+\beta_{•td}\right)}{B(\alpha_{•td},\;\beta_{•td})}. \end{array}$$

and, given $\left\{Y_{\mathrm{st}}:s\in 𝒮\right\}$ and $W_{+t}$, $\left\{W_{\mathrm{st}}:s\in 𝒮\right\}$ has multivariate hypergeometric distribution

(7) $$p\left(\left\{W_{\mathrm{st}}:s\in 𝒮\right\}\;|\;\left\{Y_{\mathrm{st}}:s\in 𝒮\right\},W_{+t}\right)=\prod_{s\in 𝒮}\left(\begin{array}{c} Y_{\mathrm{st}}\\ W_{\mathrm{st}} \end{array}\right)/\left(\begin{array}{c} Y_{+t}\\ W_{+t} \end{array}\right).$$

So, for each of *t* = 0, …, *T* and each of $d=0,\ldots ,\min(D_{t},D-1)$, the |𝒮| likelihood contributions of expression (3) are replaced by the single likelihood contribution of expression (6), and for each of *t* = 0, …, *T*, there is the additional single likelihood contribution of expression (7).

Compared to the first adapted model, the second has the advantage of involving less computation. This is because each time $\eta_{•j}$, $\psi_{•d}$, $\delta_{j}$ or a parameter of the splines underlying $g_{•t}^{W}$ and $g_{•t}^{C}$ is updated in the MCMC algorithm the single expression (6) is evaluated, rather than the |𝒮| expressions (3). The only time expression (7) is evaluated is when $Y_{\mathrm{st}}$ is updated. Note the $Z_{•td}!$ term in expression (6) can be ignored when calculating full‐conditional distributions.

### 2.4 Priors and model fitting

The intercept $\iota_{s}$ in the model for $\log\lambda_{\mathrm{st}}$ was given a non‐informative $\text{Normal}(0,10^{2})$ prior. For precision parameters $\tilde{\tau }$ and $\tau_{s}$ of the splines for $g_{\mathrm{st}}^{M}$, and the corresponding parameters for $g_{\mathrm{st}}^{W}$ and $g_{\mathrm{st}}^{C}$, we used the same inverse‐gamma prior with shape and rate 0.5 that S&E (2020a) used. The overdispersion *θ* was given an exponential prior with rate 2. This assigns substantial mass to values close to zero (no overdispersion), while still allowing considerable overdispersion. For the delay distribution, we used a uniform prior on $\psi_{\mathrm{sd}}$, assumed $\phi_{\mathrm{std}}=\phi_{d}$ and, following S&E (2020a), gave $\phi_{d}$ an exponential prior with rate 0.01. Weekday effects $\eta_{\mathrm{sj}}$ were given $\text{Normal}(0,10^{2})$ priors. The prior on the precision, $\omega^{-2}$, of the reporting‐day random effects was gamma with shape 1 and rate 0.005. This implies a prior mean of 0.125 on the standard deviation *ω*, with 90% prior CI (0.04, 0.31). The value *ω* = 0.125 corresponds to 95% of the odds ratios $\exp(\delta_{t+d})$ lying between 0.78 and 1.28; *ω* = 0.31 corresponds to them lying between 0.54 and 1.84.

The model was fitted by MCMC, using the NIMBLE package in R (de Valpine et al., 2017). R code provided by S&E (2020a) was used as a template for our code, which is provided in the Supplementary Material. We generated 300,000 iterations, discarding the first 50,000 as burn‐in.

## 3 RESULTS

Here we describe results from analysing data from all of England, focusing on the ≥75 age stratum, that with the most deaths. Further results, including for other strata and nowcasts obtained by analysing data from the seven NHS regions separately, are in the Supplementary Material. We take the delay distribution to be the same in the 45–54, 55–64 and 65–74 strata, using the second adapted model of Section 2.3.4. For these analyses, we made two modifications to the model described in Section 2. First, only 3% of delays are longer than 14 days. We grouped these into weeks, that is, delays of 15–21 days were grouped together, as were 22–28, 29–35 and 36–42 days. We also grouped delays of zero and one days, because only 1% of delays were zero days. Second, when fitting the model by MCMC, we struggled to achieve good mixing of $g_{\mathrm{st}}^{W}$ and $g_{\mathrm{st}}^{C}$. This may be due to the small number of groups of strata and large imbalance in deaths in these groups: 461, 10042 and 27020 in the 0–44, 45–74 and ≥75 groups respectively. To improve the mixing, we removed, for both $g_{\mathrm{st}}^{W}$ and $g_{\mathrm{st}}^{C}$, the spline specific to the ≥75 stratum.

**Weekday reporting effects**

**FIGURE 3:**
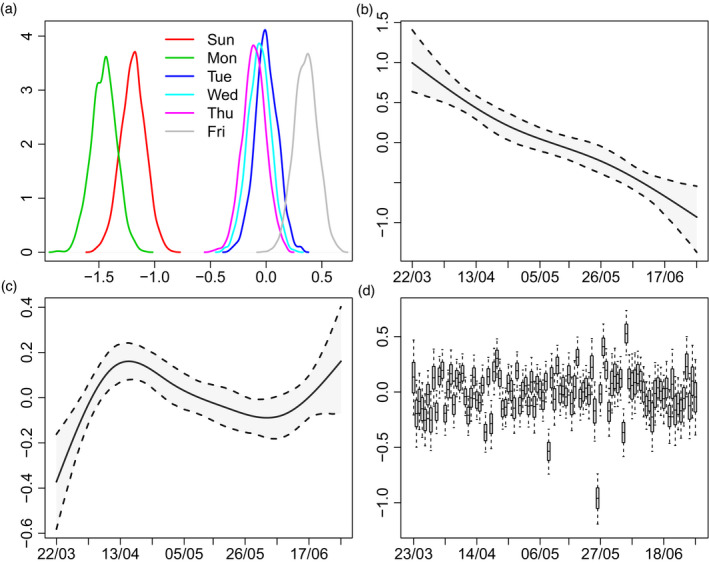
(a) Posterior distributions of weekday effects ($\eta_{5j}$); (b) posterior mean and 95% credible interval (CI) of change in Sunday/Monday effects ($g_{5t}^{W}$); (c) posterior mean and 95% CI of calendar‐time effect ($g_{5t}^{C}$); (d) posterior distributions of random effects ($\delta_{t+d}$) (boxes show posterior median and quartiles; whiskers are 95% CI).

Figure 3a shows the posterior distribution of the weekday effects $\eta_{5j}$ in the ≥75 stratum. As expected, the reporting hazard is lower on Sundays and Mondays. It is also somewhat higher on Fridays. Figure 3b shows the posterior for $g_{5t}^{W}$, the change in the Sunday and Monday effects over time. The change is substantial: the posterior mean of $g_{5t}^{W}$ decreases from around 0.7 at the end of March to around −0.9 at the end of June. Since the posterior mean of $\eta_{57}$ is −1.2, this means that the odds ratio of reporting on Sunday relative to Saturday (recall that Saturday is baseline) is around  exp (−1.2 + 0.7) = 0.6 in late March but  exp (−1.2 − 0.9) = 0.12 in late June.

**Calendar‐time reporting effects**

Figure 3c shows the posterior for the calendar‐time effect $g_{5t}^{C}$ in the ≥75 stratum. Delays become shorter until mid‐April (the epidemic peak), then lengthen, and finally shorten again.

**Reporting day random effects**

Figure 3d shows the posteriors for random effects $\delta_{0},\ldots ,\delta_{T}$. The largest is for 26 May, with a posterior mean of −1. This is unsurprising: Figure 1b shows the number of reports on Tuesday 26 May is much lower than the numbers on each of the preceding Tuesday to Saturday and following Wednesday to Friday. Although it is similar to the numbers on the preceding 2 days, these are Sunday and Monday, when numbers are expected to be low. The posterior mean of *ω*, the standard deviation of the random effects, is 0.23, with 95% posterior CI (0.19, 0.29).

**Overdispersion parameter** ***θ*** **for** $Y_{\mathrm{st}}$

The posterior mean of *θ* is 0.30, with 95% posterior CI (0.08, 0.55). This suggests mild Poisson overdispersion: the variance of $Y_{\mathrm{st}}$ is around 1.3 times as large as its expectation.

**Nowcasts**

**FIGURE 4:**
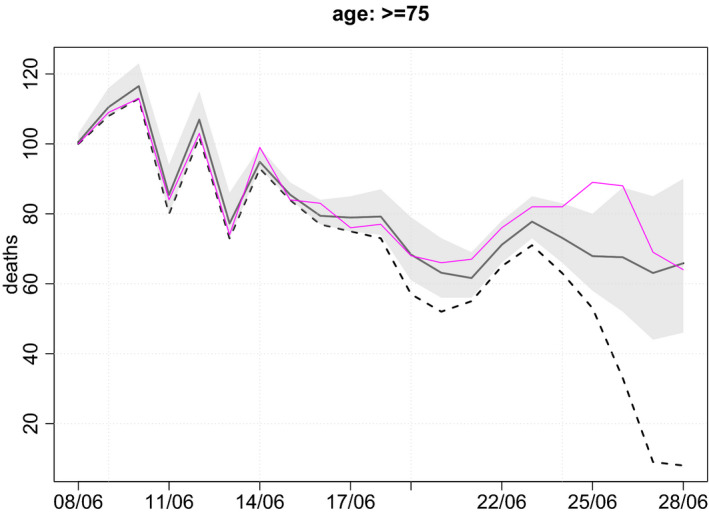
Estimated numbers of deaths occurring on each day (black line) in the ≥75 stratum, with corresponding numbers of deaths reported by 29th June (broken line) and the true numbers (purple line). Posterior 95% credible intervals are shown by shaded region. For ease of viewing, only most recent 21 days are shown.

Figure 4 shows the nowcast for the ≥75 stratum. Nine and eight deaths occurred on 27 and 28 June, respectively, and were reported by 29 June. The corresponding estimated numbers of deaths are 63 and 66. So, an estimated 9/63 = 14% of deaths on Saturday 27 June were reported within 2 days, and 12% of deaths on Sunday 28 June were reported within 1 day. These percentages may seem low, considering that Figure 2 shows around 26% of deaths are reported within one day and around 60% within 2 days. There are two reasons for this. First, reporting is lower on Sundays and Mondays than during the rest of the week, especially more recently. Second, the estimates for the days before 27 June were all above 60 and it is unlikely that deaths would suddenly drop far below 60. The 95% posterior CIs for these two days are particularly wide (around 45–90).

By 9 August (i.e. 42 days after 28 June), the true numbers of deaths that occurred on each day (and were reported within 42 days) were known. Figure 4 shows these true numbers. Of the 21 true numbers, 19 lie within their corresponding 95% CI (and one lies just outside). This is roughly as expected: 19/21 = 90.5%.

**Probability of increase in deaths in recent days**

It is useful to be able to detect early signs of an increase in deaths in recent days. Figure S20 shows, for each stratum, the posterior probability that the number of deaths in the most recent *x* days was greater than the number in the preceding *x* days (*x* = 1, …, 7). There is no evidence of an increase. However, many of the probabilities are around 0.5, meaning that deaths are as likely to be increasing as they are to be decreasing.

## 4 EVALUATION OF PERFORMANCE OF NOWCASTS

As mentioned earlier, 42 days after a nowcast is made, the true numbers of deaths are known. This enables us to evaluate the performance of our nowcasts. For this purpose, we carried out a nowcast every 5 days from 29 June 2020 to the end of March 2021, in each of the seven NHS regions separately and for all of England (a total of 56 × 8 = 448 nowcasts). For the nowcast based on data available on day *T* we calculated, for each of days *t* = *T* − 1, *T* − 2, …, *T* − 7, the difference between the posterior mean of the number of deaths that occurred on day *t* and the true number. We also examined whether the corresponding 95% posterior CI included the true value. We shall refer to the average difference between the posterior mean and the true value as the ‘bias’, and to the percentage of CIs that include the true value as the ‘coverage’. To prevent computation time increasing as *T* increases, the nowcasting model was fitted only to data on the most recent 90 days, that is, days *T*, *T* − 1,…, *T* − 89. On 12 August 2020, PHE revised its definition of a COVID‐19 death to include only deaths within 28 days of a positive test (Public Health England, 2020). So, for nowcasts after 12 August, we used only those deaths.

Table 1 shows the bias and coverage for each age group and for regional and nationwide nowcasts. The bias is small (<8%) relative to the average true number of deaths per day (the ‘mean’ column of Table 1), with the exception of the nowcasts for day *T*−1 in the 0–44 age group in the regions. Here the average true number of deaths is only 0.4, whereas the average nowcast is 0.47, a bias of 17%. Coverage of 95% CIs is at least 91%, except for nowcasts of the ≥75 age group in England, where it falls to 88% for nowcasts of four or more days ago. The coverage is higher than 95% when the average true number of deaths is small, that is, for groups 0–44 and 45–54 in England and all groups except ≥75 in the regions. This is due to the discreteness of the data. That is, when the true number of deaths on a day is very low (e.g. zero or one), the 95% and 99% CIs will often be the same: both equal to [0, 1]. In such cases, it would be expected that more than 95% of the 95% CIs would include the true value. For groups 0–44 and 45–54 in England and all groups except ≥75 in the regions, the percentage of days on which the true number of deaths is zero or one (the ‘low’ column of Table 1) is at least 30% and can be as high as 90%.

**TABLE 1:** Bias and coverage for nowcasts. Results for pairs of days *T* − 2 and *T* − 3, and *T* − 4 and *T* − 5, and *T* − 6 and *T* − 7 are averaged, as are results for the seven regions. Bias is mean difference between estimated and true number of deaths. Coverage is percentage of 95% credible intervals (CIs) that contain true number of deaths

		Bias for day *T* minus					Coverage for day *T* minus				
Area	Age	1	2/3	4/5	6/7	mean${\;}^{a}$	1	2/3	4/5	6/7	low${\;}^{b}$
England	0–44	−0.09	0.01	−0.04	0.05	2.9	96	98	96	98	54.1
	45–54	−0.54	−0.54	−0.53	−0.25	6.8	98	96	95	97	30.9
	55–64	−0.38	−0.11	−0.19	−0.28	18.4	96	96	94	95	18.1
	65–74	−1.21	1.16	0.18	0.44	41.3	91	91	93	94	9.4
	≥75	1.37	−0.75	−0.31	−0.74	166.0	93	92	88	88	1.0
Regions	0–44	0.07	0.03	0.00	0.00	0.4	98	98	98	98	90.1
	45–54	−0.03	−0.07	−0.07	−0.04	1.0	98	98	98	99	77.0
	55–64	0.00	0.01	−0.02	−0.04	2.6	99	99	98	98	57.8
	65–74	−0.01	0.22	0.04	0.06	5.9	96	98	97	99	40.7
	≥75	0.28	−0.06	−0.02	−0.16	23.7	97	96	96	96	18.5

${\;}^{a}$The ‘mean’ column contains the average true number of deaths in the age group.

${\;}^{b}$The ‘low’ column contains the percentage of days on which the true number of deaths was zero or one.

## 5 DISCUSSION

Timely monitoring of the number of COVID‐19‐related deaths relies on methods that adjust the observed data for reporting delay. These methods need to be flexible enough to deal with the peculiar reporting patterns of these data and the way these patterns change over time. Here we built on the approach of S&E and derived age‐specific estimates of the number of deaths occurring in each of the seven English NHS regions.

The modelling of weekday and calendar‐time effects described in Sections 2.3.1 and 2.3.2 is different from the way S&E (2020a, 2020b, 2020a, 2020b) modelled them. If weekday effects are removed from our model, it becomes what S&E (2020b) called a ‘Survivor Model’ with calendar‐time effects but without weekday effects. Figure S19 provides some support for this way of modelling the calendar‐time effect, suggesting that it is indeed additive on the probit scale, at least for delays of two or more days. Whereas we apply the weekday effects to the hazard in Equation (4), S&E's model replaces Equations (4) and (5) with $\text{probit}\left(q_{\mathrm{std}}\right)=\text{probit}\left(q_{\mathrm{sd}}^{*}\right)+g_{\mathrm{st}}^{C}+h_{\mathrm{st}},$ where $h_{\mathrm{st}}$ is a cyclic cubic spline with a period of 7 days (i.e. $h_{\mathrm{st}}=h_{s,t+7}$). We believe that applying the weekday effects in Equation (4) more accurately reflects these effects. Suppose, for example, that deaths are never reported on Sundays. This would be captured by our model through $\eta_{7}=-\infty$, but would not be captured by the spline $h_{\mathrm{st}}$, because $h_{\mathrm{ts}}$ is a function of the weekday on which the individual died, rather than the weekday on which this death might be reported (see Supplementary Material for further explanation). That weekday affects hazard of reporting is demonstrated by Figure S21, which shows the estimated distribution of delay by weekday of death. The probability of reporting within 1 day is approximately the same for deaths occurring on any of Monday to Friday, but is lower for deaths occurring on Saturday or Sunday. The probability of reporting within 2 days is the same for deaths occurring on Monday to Thursday, but lower for those occurring on Friday and Saturday (and to some extent Sunday). For reporting within 3 days, the probability is the same for deaths on Monday to Wednesday, but lower for Thursday to Saturday.

An alternative way to model the calendar‐time effect would be to apply it, together with the weekday effect, to the hazard in Equation (4). This would yield what S&E (2020b) call a ‘Hazard Model’. However, S&E (2021, 2020a, 2020b) argue that the Hazard Model formulation is less parsimonious than their Survival Model formulation (and hence our model), because it requires multiple splines for the calendar‐time effect in each stratum: one for each possible delay *d*.

To generate the results described in Section 3, the computation time required to fit the model was 5.0 h using one core of a MacBook Pro with a 2.6 GHz 6‐core Intel i7 processor. This time was for the second adapted model of Section 2.3.4 and represents a reduction compared to that needed for the first adapted model (7.7 h). If computation time were a concern, two measures that could be taken to reduce it, in addition to the measures already described, would be to decrease the maximum delay *D* and to use more grouping of longer delays. We adjusted for delays of up to *D* = 42 days, whereas S&E (2020a) used only *D* = 14. Overall, around 97% of deaths reported within 42 days are reported within 14 days, and so this restriction may be reasonable. We distinguished between delays of *d* and *d*+1 days all the way up to *d* = 14 days, grouping longer delays into weeks. S&E (2020a) instead grouped together all delays of more than 7 days. This reduces computation time, but has the drawback of discarding some information. Specifically, data on deaths that occurred between 8 and 13 days ago and were reported with delays of more than 7 days are ignored. As well as potentially reducing statistical efficiency, this discarding of information allows the estimated number of deaths that occurred on any of these days to be lower than the number so far observed to have occurred on that day. It was for these reasons that we waited until 15 days before grouping delays and thereafter grouped them into weeks rather than into a single group.

Several research groups have applied nowcasting methods to COVID‐19 deaths or diagnoses (which are also subject to reporting delays). Bird and Nielsen (2020) nowcasted deaths in England, using a modification of the chain‐ladder approach employed in general insurance. They used only data from the previous 7 days and estimated numbers of deaths reported within 7 days. Greene et al. (2021) nowcasted cases (diagnoses) in New York City using a Bayesian method proposed by McGough et al. (2020). This method, also an adaptation of the chain‐ladder approach, assumes that the number of cases that occur on day *t* and are reported with delay *d* has a negative‐binomial distribution with mean equal to the expected incidence of positive tests at time *t* multiplied by an effect of *d*. The expected incidence over time is modelled as a random‐walk process, and the effect of *d* is assumed constant over the 21‐day period from which data are used. De Salazar et al. (2022) used the same approach to nowcast symptomatic cases by date of symptom onset in two Spanish regions. Sarnaglia et al. (2021) nowcasted deaths in Brazil, taking a similar approach to Greene et al. (2021) but allowing the delay distribution to change over time according to a AR1 process. Abbott et al. (2020) nowcasted cases separately in several countries. They assumed that reporting delays have a log normal distribution that remains constant over a 12‐week period. Sahai et al. (2022) nowcasted cases in Ohio, using a random forest to predict the proportion of cases reported thus far. The covariates used by the random forest are functions of the delay, the weekday of the report, and the number of cases that occurred on each day and were reported with each delay. The nowcast was calculated as the number of cases reported thus far divided by the estimated proportion reported thus far. Uncertainty associated with this nowcast was not quantified. Hawryluk et al. (2021) nowcasted weekly deaths in Brazil. They aggregated daily data into weeks and assumed that the number of deaths that occurred in a given week and were reported in another week has a negative binomial distribution with a mean that follows a Gaussian process with separable two‐dimensional kernel over week of death and number of weeks of delay. Glöckner et al. (2020) applied the method of Höhle and an der Heiden (2014) to nowcast cases in Japan. This method is similar to that of S&E (2020a, 2020b, 2020a, 2020b), but uses a 14‐day moving window to estimate the delay distribution. Günther et al. (2021) applied a modified version of the same method to nowcast cases in Bavaria. This assumes a random walk for the incidence of positive tests over time and allows both a piecewise‐linear effect of test time and a weekend effect on the delay distribution. However, it uses the more restrictive multinomial distribution for case reports, in place of S&E's (and our) GDM distribution. Hildebrandt et al. (2021) described an implementation of Günther et al.'s method to nowcast cases in Germany at the level of district or state. Bergström et al. (2022) extended Günther et al.'s method to incorporate additional data on a second event that reflects an earlier stage in disease progression than the event being nowcasted. In their application to Swedish data, they nowcasted COVID‐19 deaths using additional data on ICU admissions. This model assumes that the log expected number of deaths occurring on a given day is a linear combination of the log expected number of deaths on the previous day and the log observed number of ICU admissions a fixed number of days earlier. In principle, this approach could also be used in our model. The log expected number of deaths ($\log\lambda_{\mathrm{st}}$) would then be equal to the sum of two spline functions and the lagged log number of ICU admissions.

As mentioned in Section 1, S&E (2020a) used reports from NHSE to nowcast deaths over the period 2 April to 5 May 2000 in seven English regions (not broken down by age). Using the same data, Stoner et al. (2020) found that the coverage of their 95% credible intervals was 95% for days *T* and *T* − 1 (i.e. same‐day and previous‐day nowcasts), 91% for day *T* − 2 and 84% for day *T* − 3. In the comparison with the nowcasting methods of McGough et al. (2020) and Bastos et al. (2019), they found their method yielded more precise estimates and credible intervals with better coverage for nowcasts of day *T*.

More recently, two other groups have adapted S&E's hierarchical model and nowcasted COVID‐19 cases by region. Jersakova et al. (2021) assume a negative‐binomial model for the number of cases on each day, with expectation described by a random walk with drift modified by weekend effects. A beta‐binomial distribution is used for the number of these cases reported thus far, with an empirical Bayes approach to choosing the parameters of the beta distribution. Data on delays from the previous 14 days are used, with the delay distribution assumed constant over this period. The model is applied to data at the level of English lower‐tier local authorities and there is an element of borrowing of information on delays (but not on numbers of cases) from neighbouring local authorities. Kline et al. (2021) nowcast cases in the counties of Ohio state. They argue that an autoregressive model is more suitable than S&E's spline model for the incidence of cases when temporal trends may be quite different across counties. They assume the expected number of cases in a county over time follows a semi‐local linear trend model modified by a weekday effect and with an intrinsic conditional autoregressive element to capture spatial correlation across counties. The delay distribution is modelled (almost) independently in each county and allowed to change over time according to a AR1 process, although the autoregressive parameter is common to all counties and a hierarchical structure is assumed for weekday effects. It would be interesting to explore whether this very flexible model would work when nowcasting deaths, which are much smaller in number than cases.
