# Supplementary material for: Nowcasting COVID‐19 deaths in England by age and region
Source: J R Stat Soc Ser C Appl Stat. 2022 Jun 15:10.1111/rssc.12576. Online ahead of print. doi: 10.1111/rssc.12576 (PMC9349735; doi:10.1111/rssc.12576)
Supplement: Supplementary file 1 [file RSSC-9999-0-s002.tar › RcodeNowcasting/figures/dataset_2020-10-11-reportingdelay.pdf]

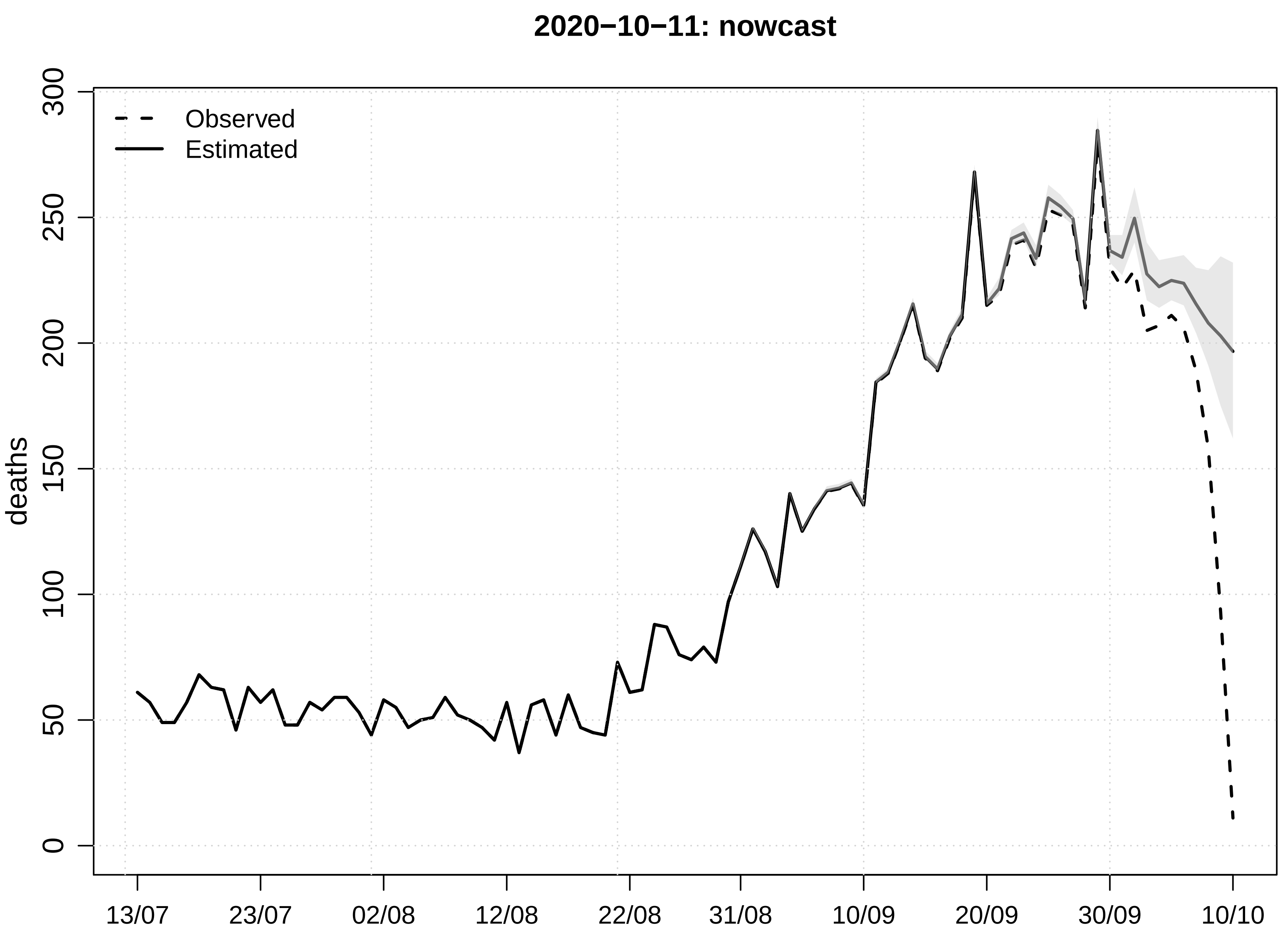
2020−10−11: nowcast
Observed
Estimated
deaths
0
50
100
150
200
250
300
13/07
23/07
02/08
12/08
22/08
31/08
10/09
20/09
30/09
10/10

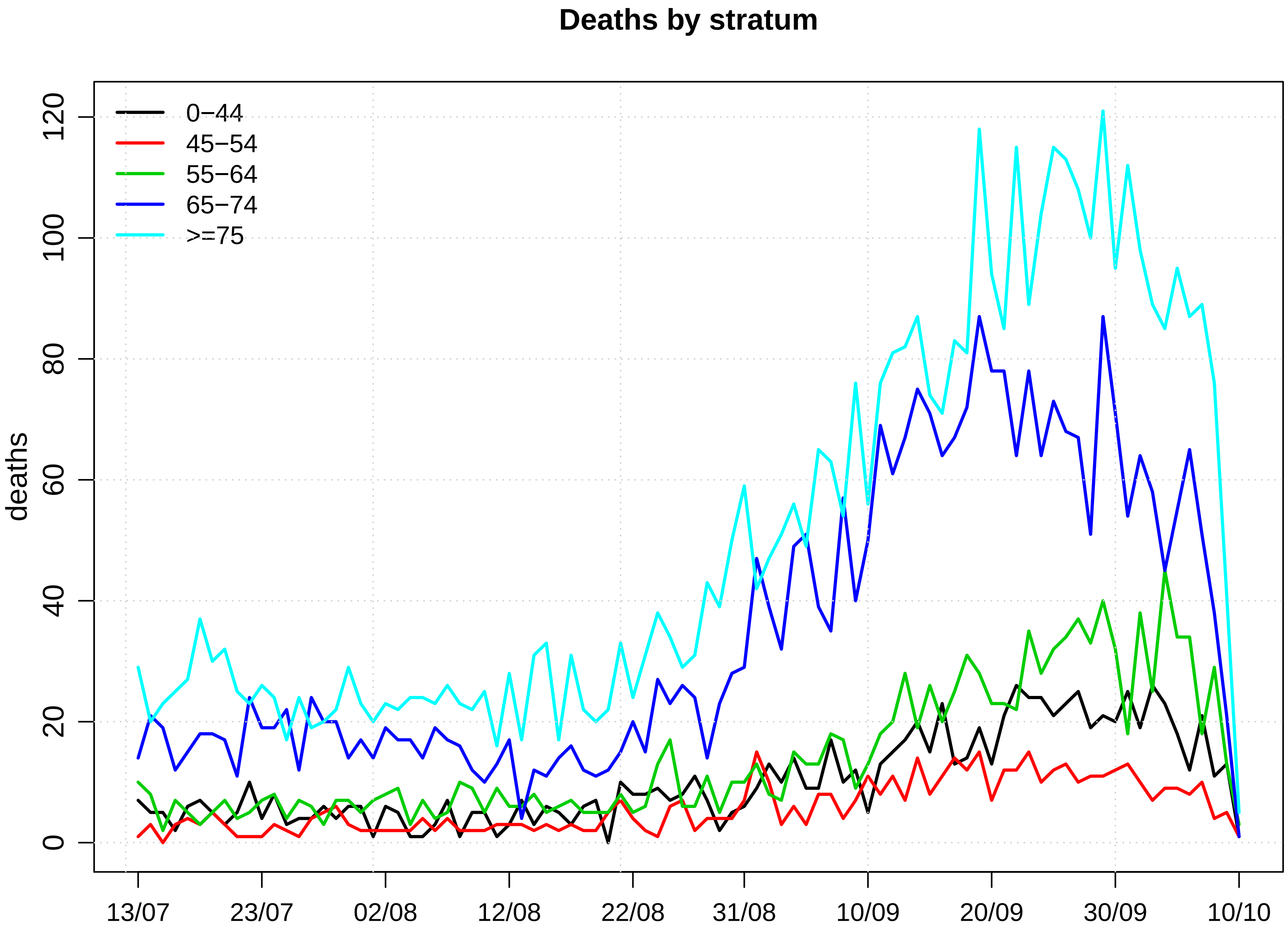

Deaths by stratum
0−44
45−54
55−64
65−74
>=75
deaths
0
20
40
60
80
100
120
13/07
23/07
02/08
12/08
22/08
31/08
10/09
20/09
30/09
10/10

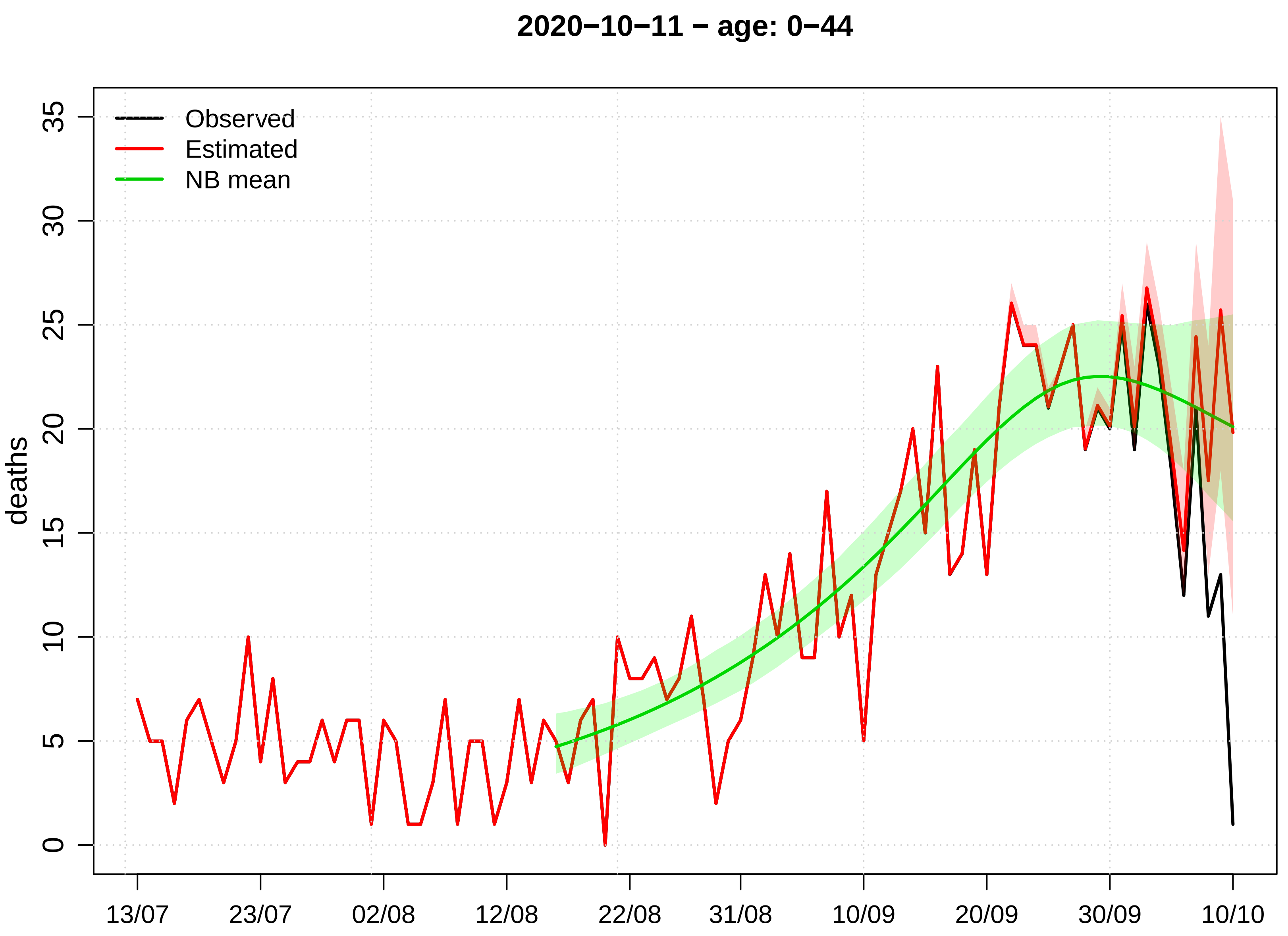
2020−10−11 − age: 0−44
Observed
Estimated
NB mean
deaths
0
5
10
15
20
25
30
35
13/07
23/07
02/08
12/08
22/08
31/08
10/09
20/09
30/09
10/10

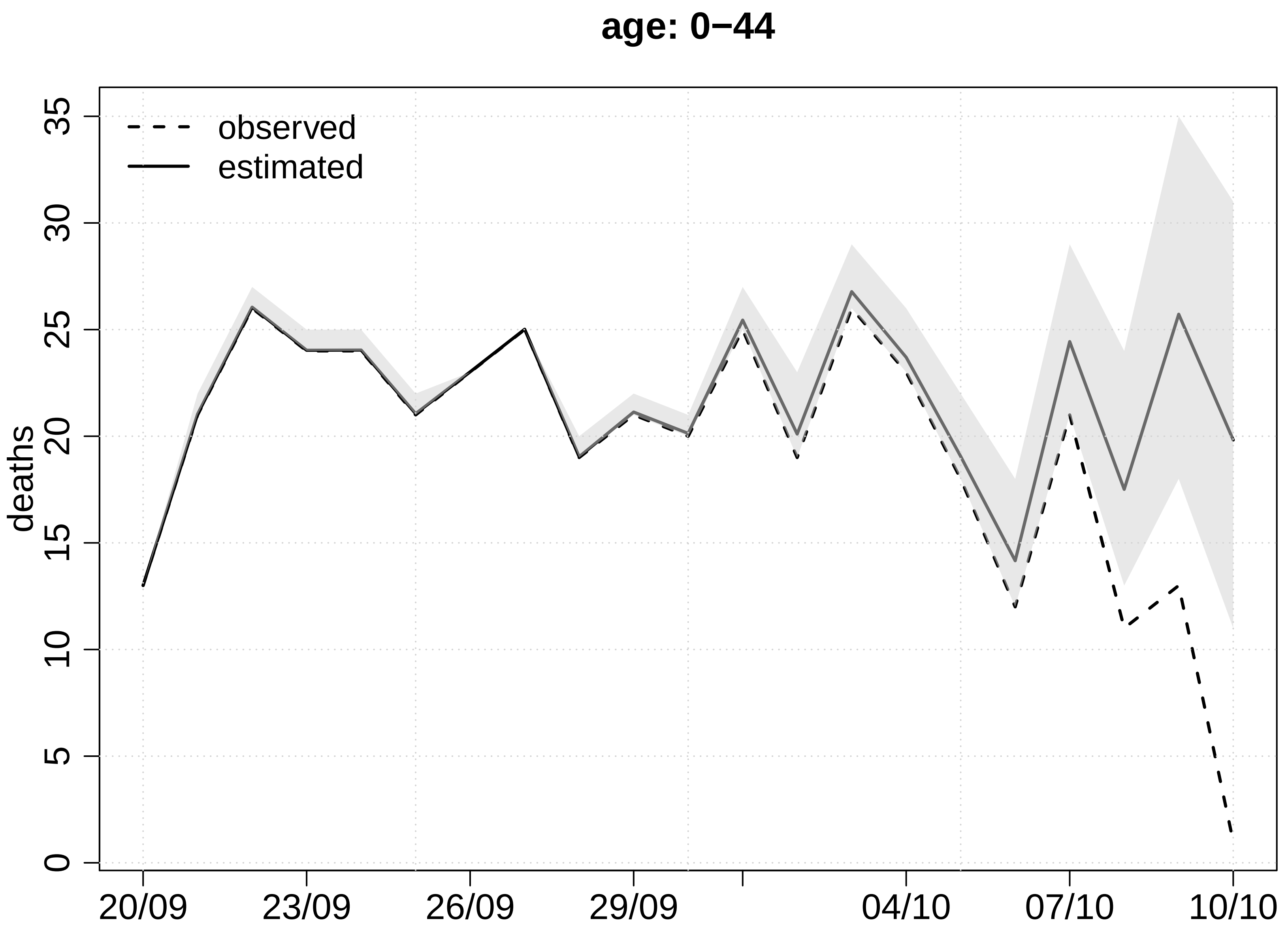
age: 0−44
observed
estimated
deaths
0
5
10
15
20
25
30
35
20/09
23/09
26/09
29/09
04/10
07/10
10/10

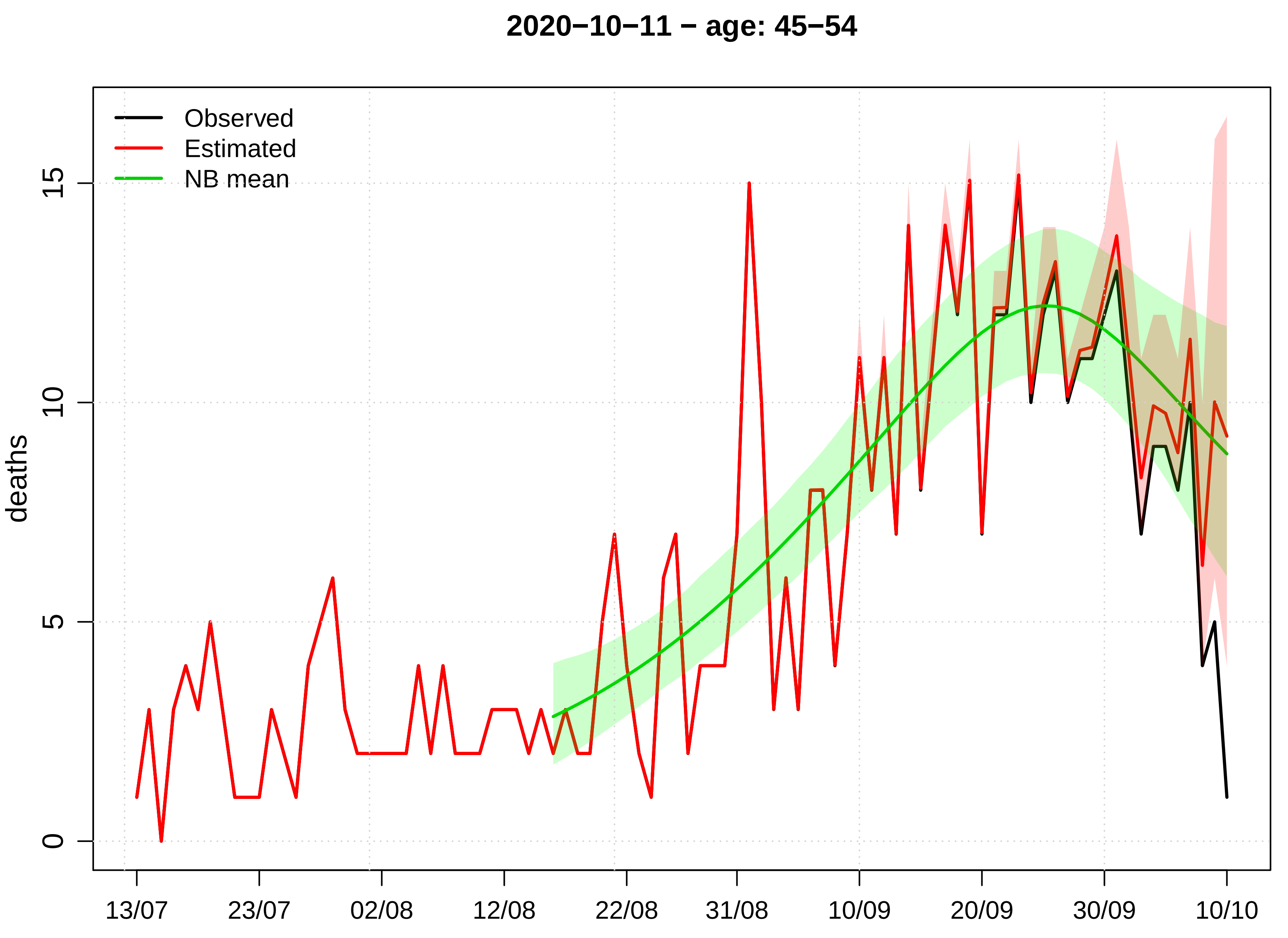

2020−10−11 − age: 45−54
Observed
Estimated
NB mean
deaths
0
5
10
15
13/07
23/07
02/08
12/08
22/08
31/08
10/09
20/09
30/09
10/10

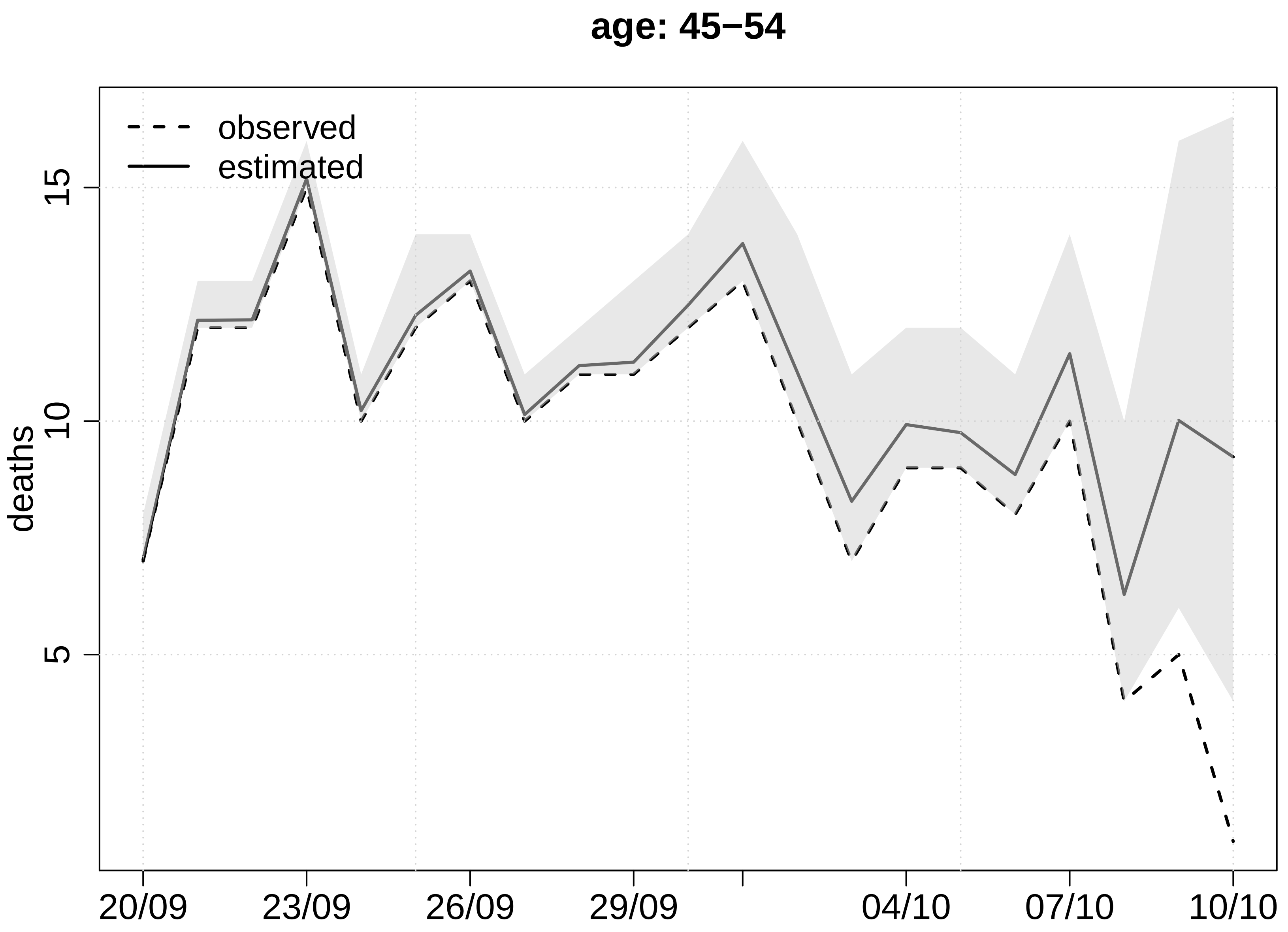

age: 45−54
observed
estimated
deaths
15
10
5
20/09
23/09
26/09
29/09
04/10
07/10
10/10

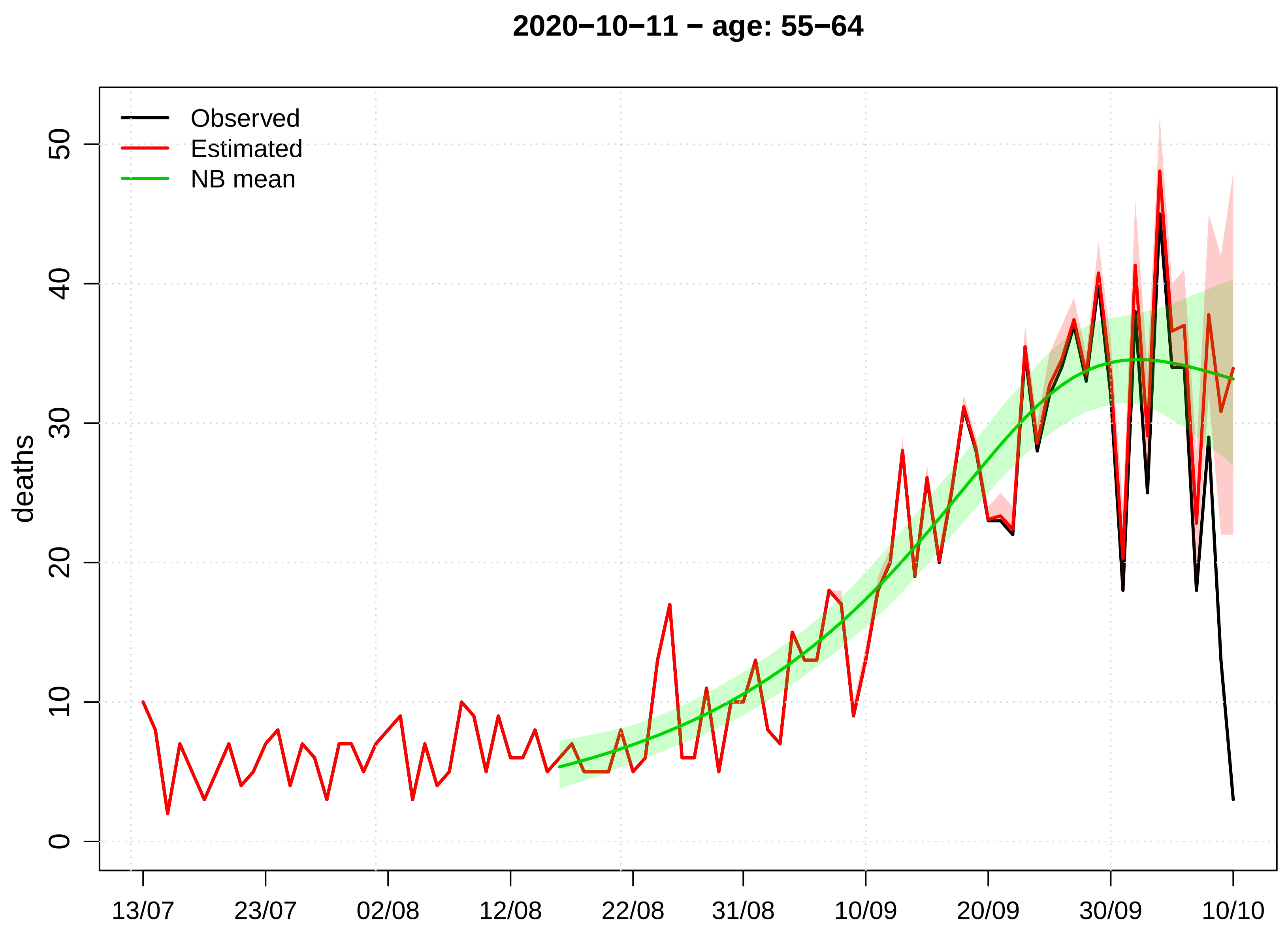
2020−10−11 − age: 55−64
Observed
Estimated
NB mean
deaths
0
10
20
30
40
50
13/07
23/07
02/08
12/08
22/08
31/08
10/09
20/09
30/09
10/10

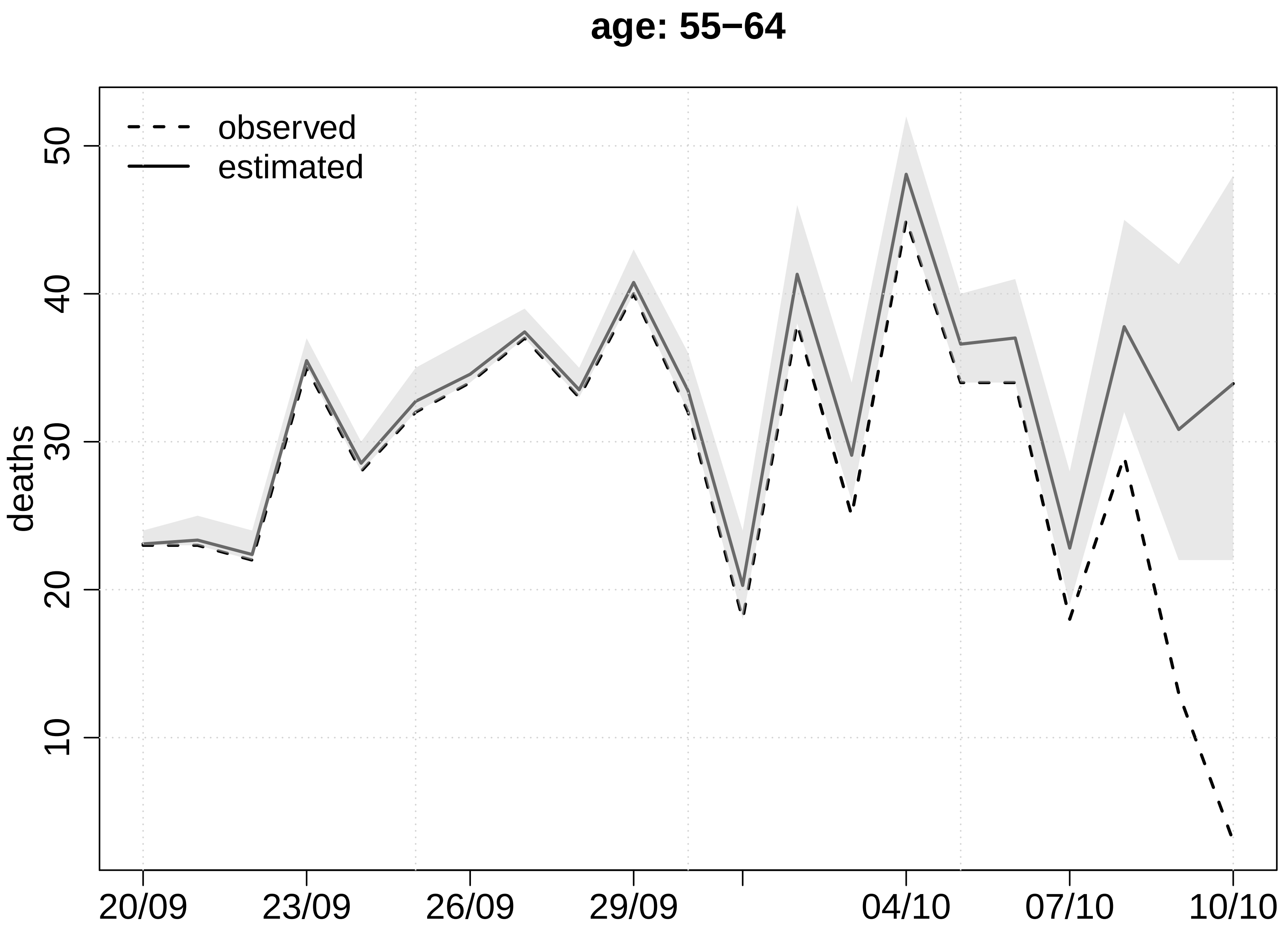
age: 55−64
observed
estimated
deaths
10
20
30
40
50
20/09
23/09
26/09
29/09
04/10
07/10
10/10

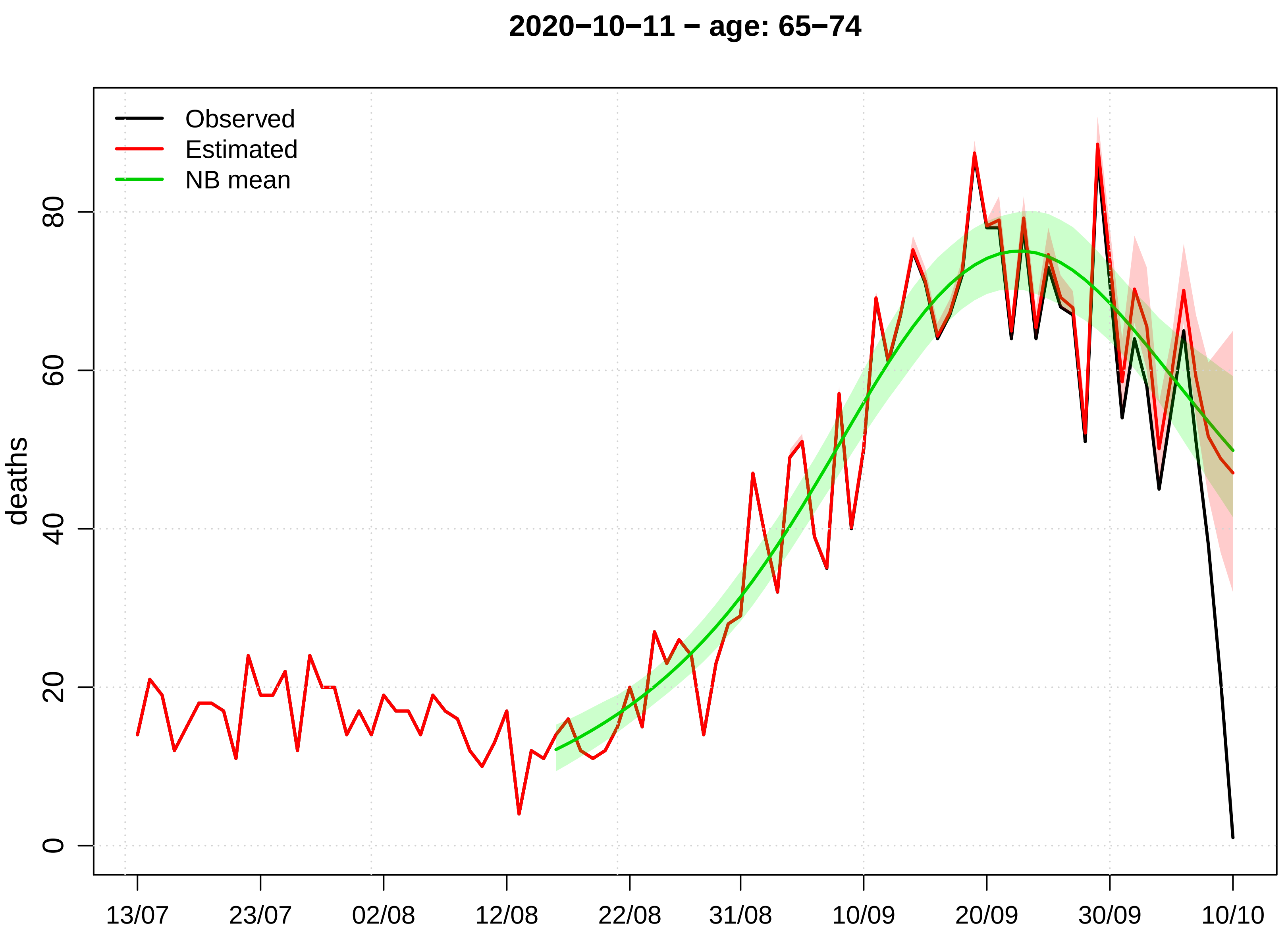
2020−10−11 − age: 65−74
Observed
Estimated
NB mean
deaths
0
20
40
60
80
13/07
23/07
02/08
12/08
22/08
31/08
10/09
20/09
30/09
10/10

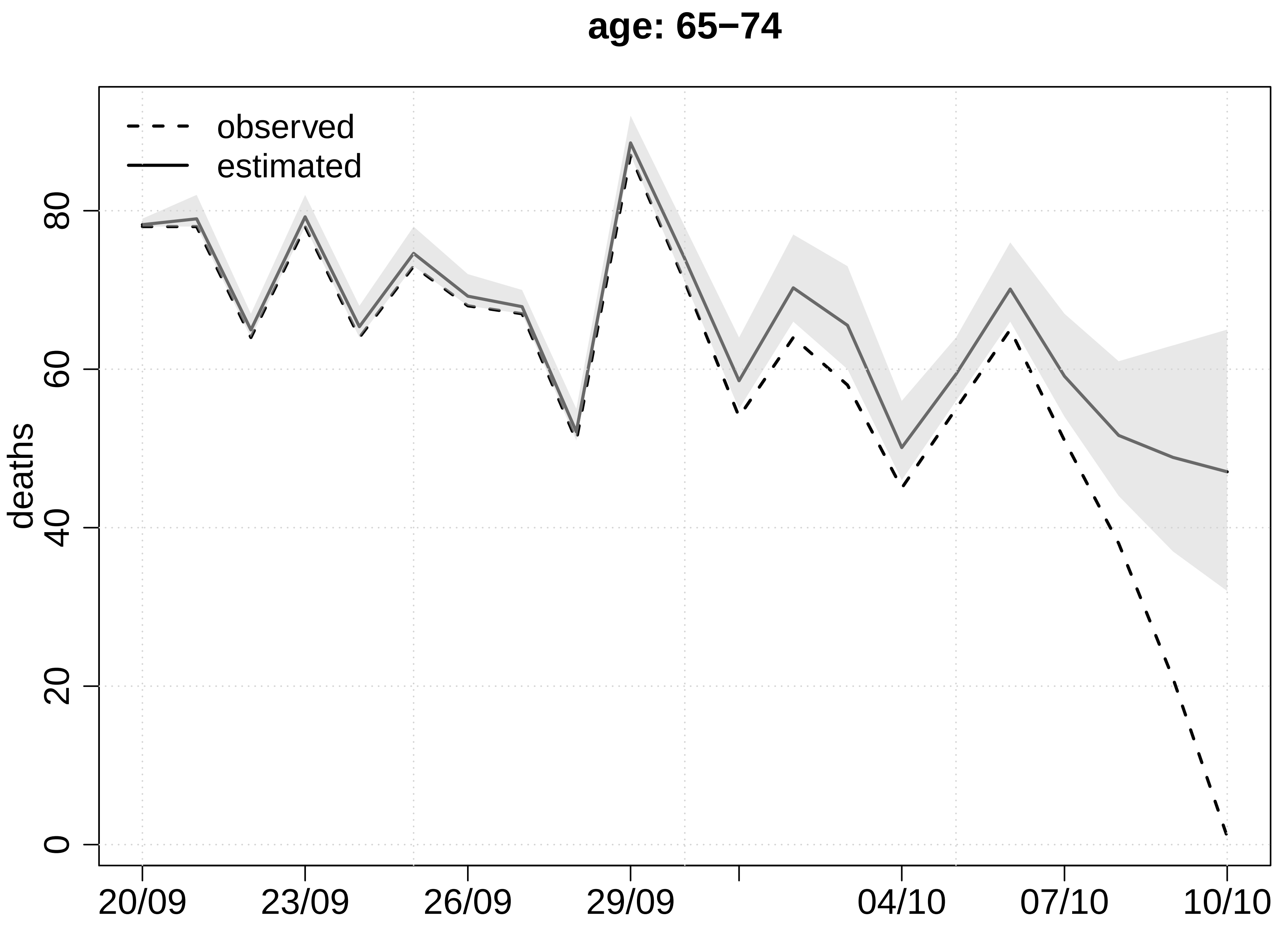
age: 65−74
observed
estimated
deaths
0
20
40
60
80
20/09
23/09
26/09
29/09
04/10
07/10
10/10

2020−10−11 − age: >=75

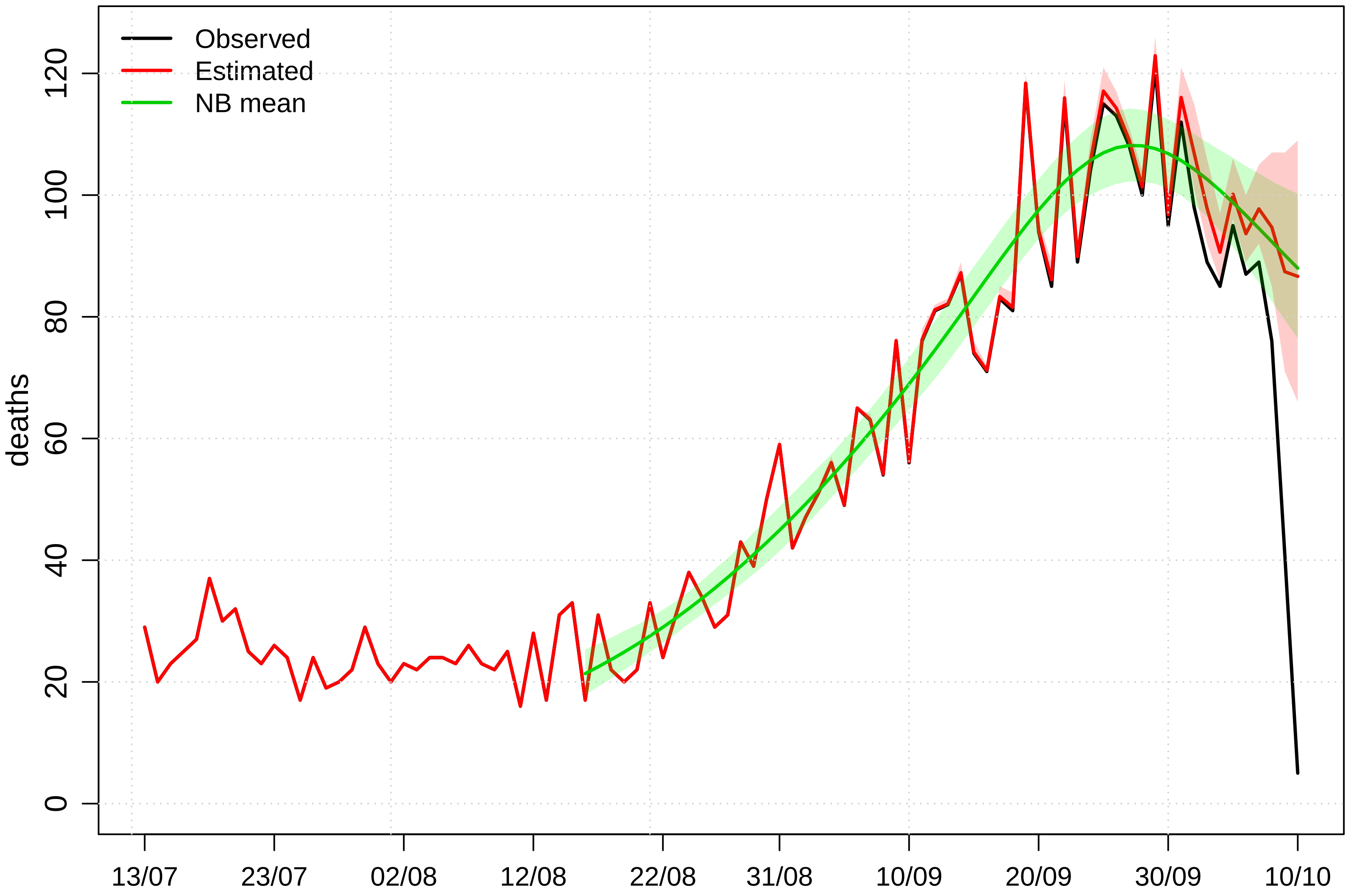

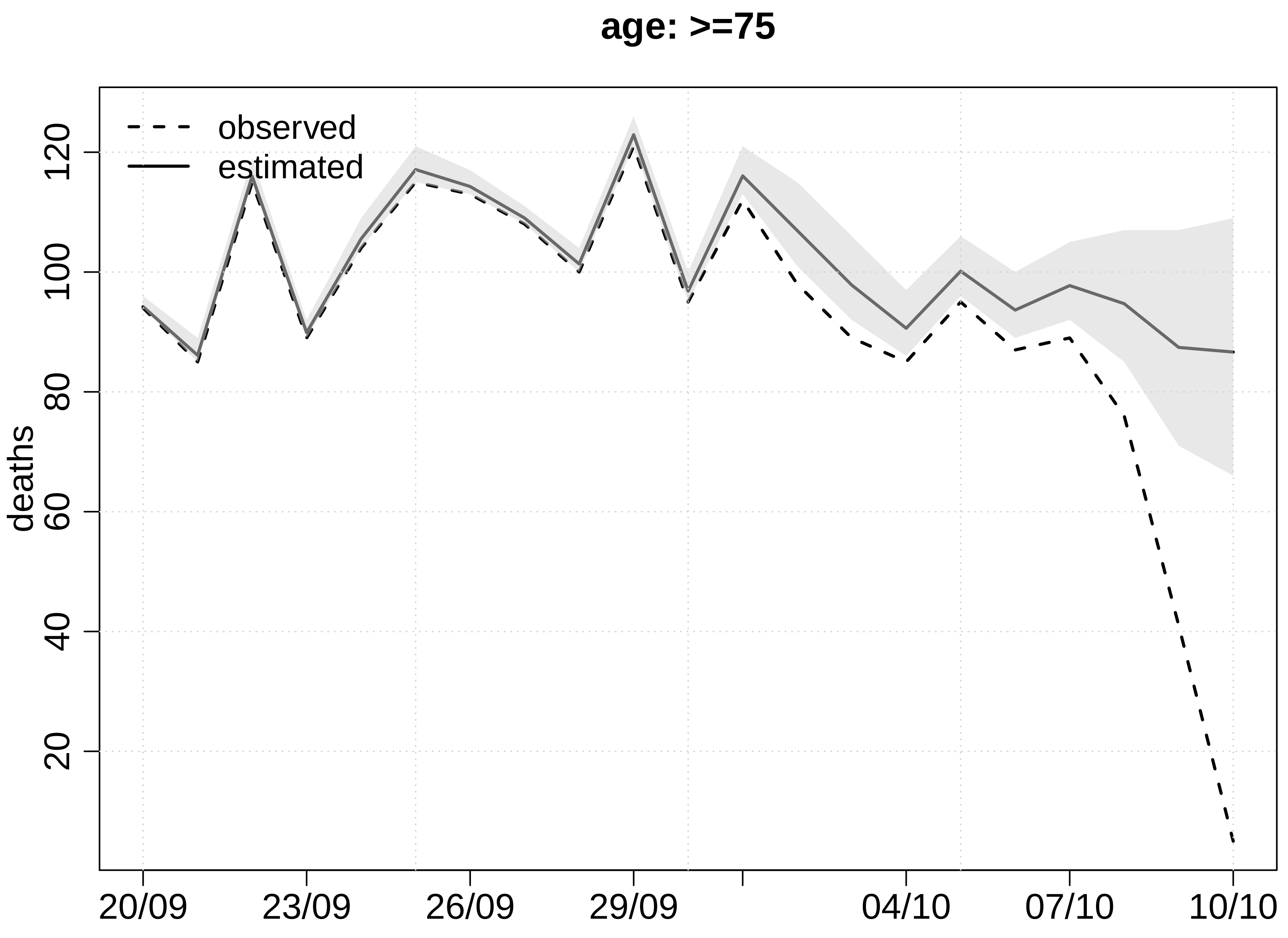
age: >=75
observed
estimated
deaths
120
100
80
60
40
20
20/09
23/09
26/09
29/09
04/10
07/10
10/10

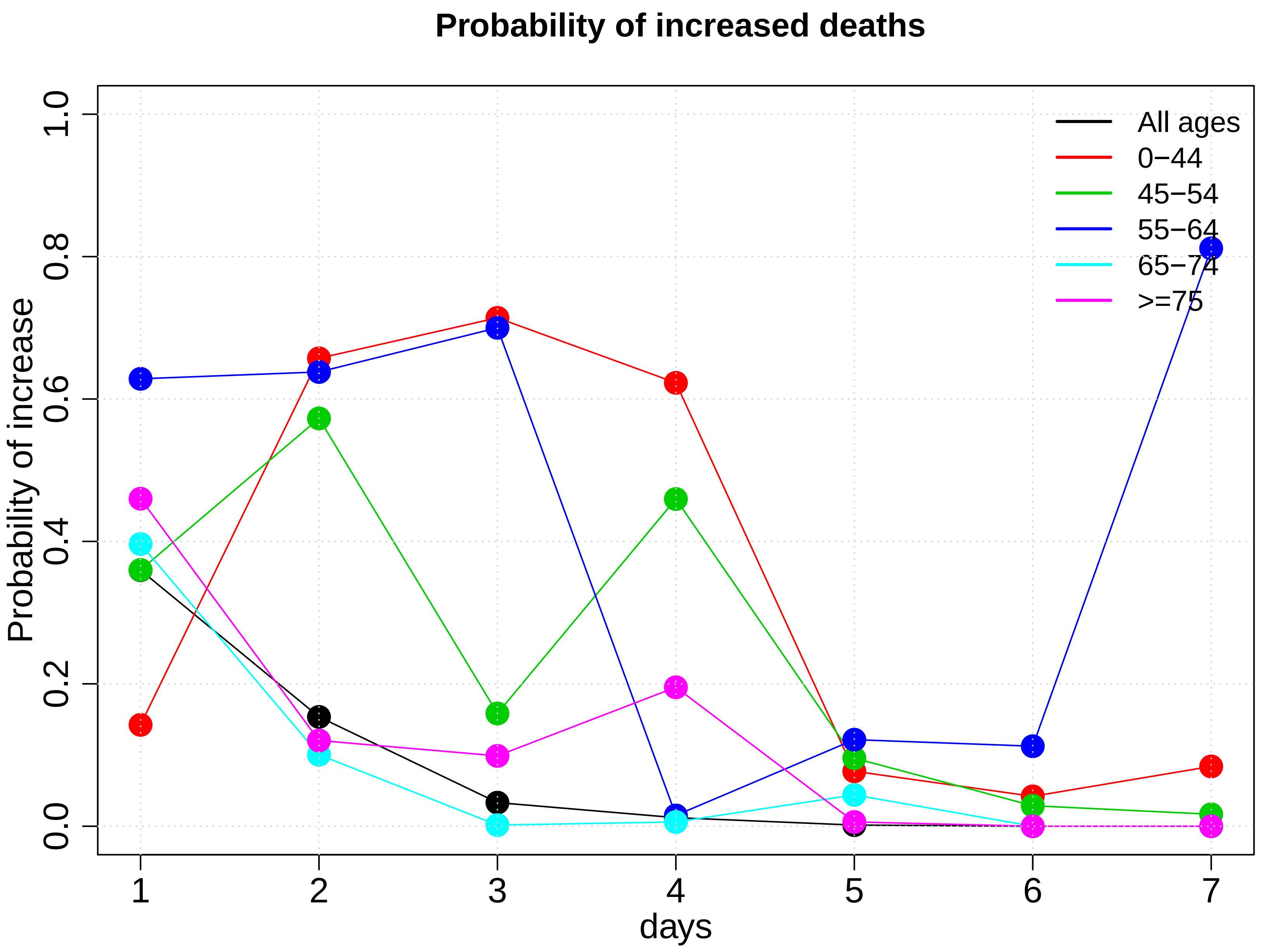
Probability of increased deaths
Probability of increase
days
All ages
0−44
45−54
55−64
65−74
>=75
0.0
0.2
0.4
0.6
0.8
1.0
1
2
3
4
5
6
7

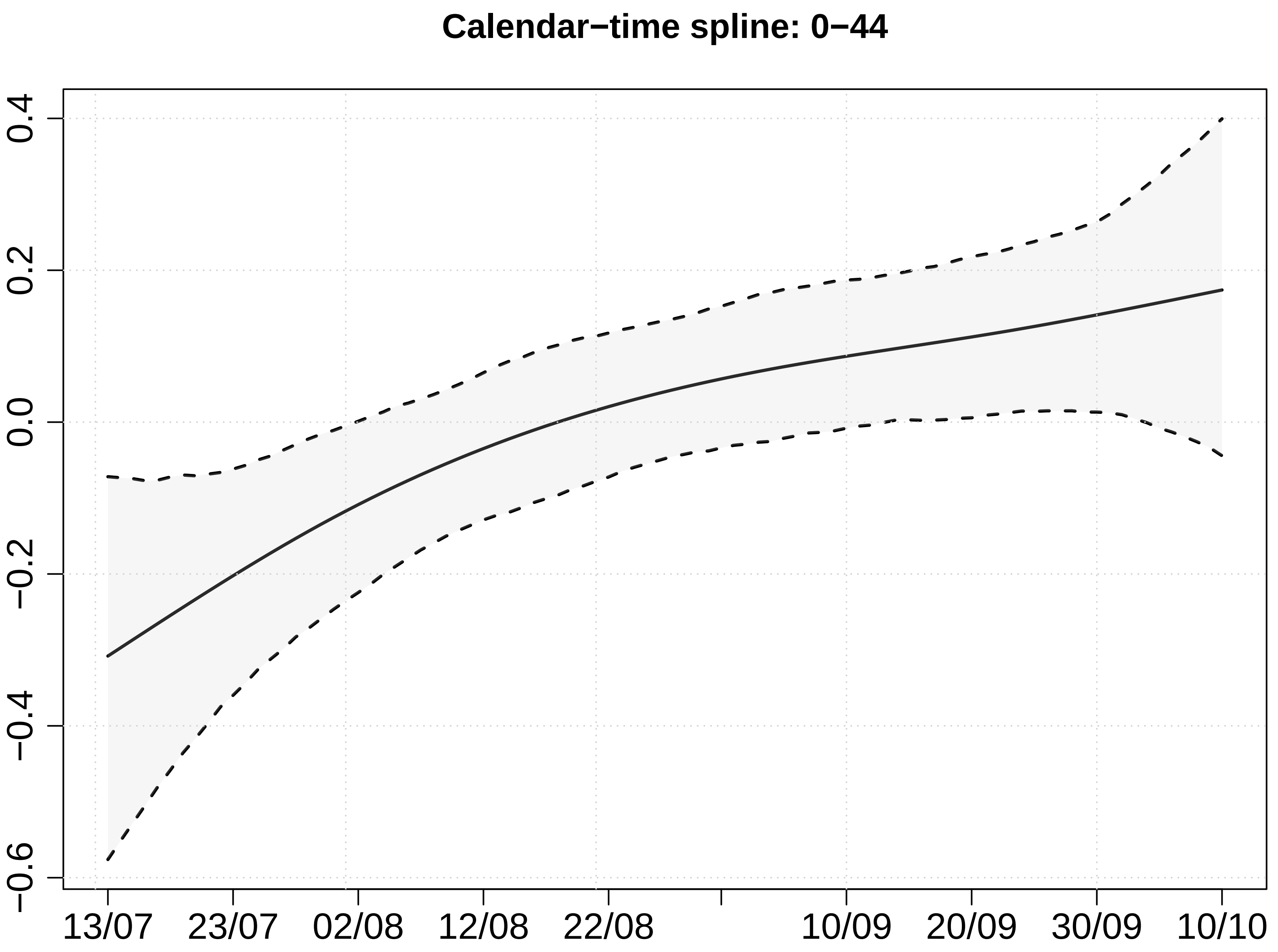

Calendar−time spline: 0−44
0.4
0.2
0.0
−0.2
−0.4
−0.6
13/07
23/07
02/08
12/08
22/08
10/09
20/09
30/09
10/10

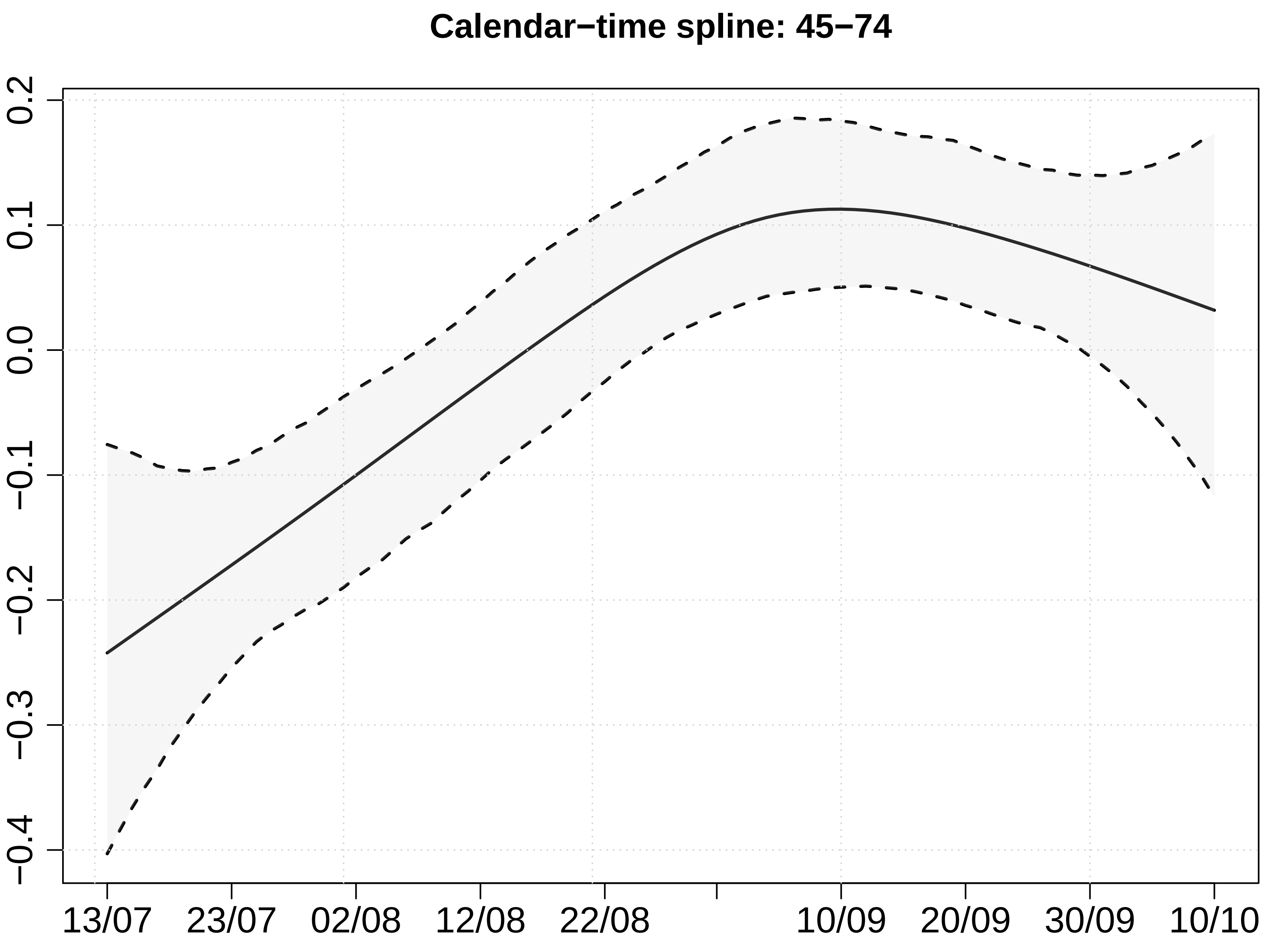

Calendar−time spline: 45−74
0.2
0.1
0.0
−0.1
−0.2
−0.3
−0.4
13/07
23/07
02/08
12/08
22/08
10/09
20/09
30/09
10/10

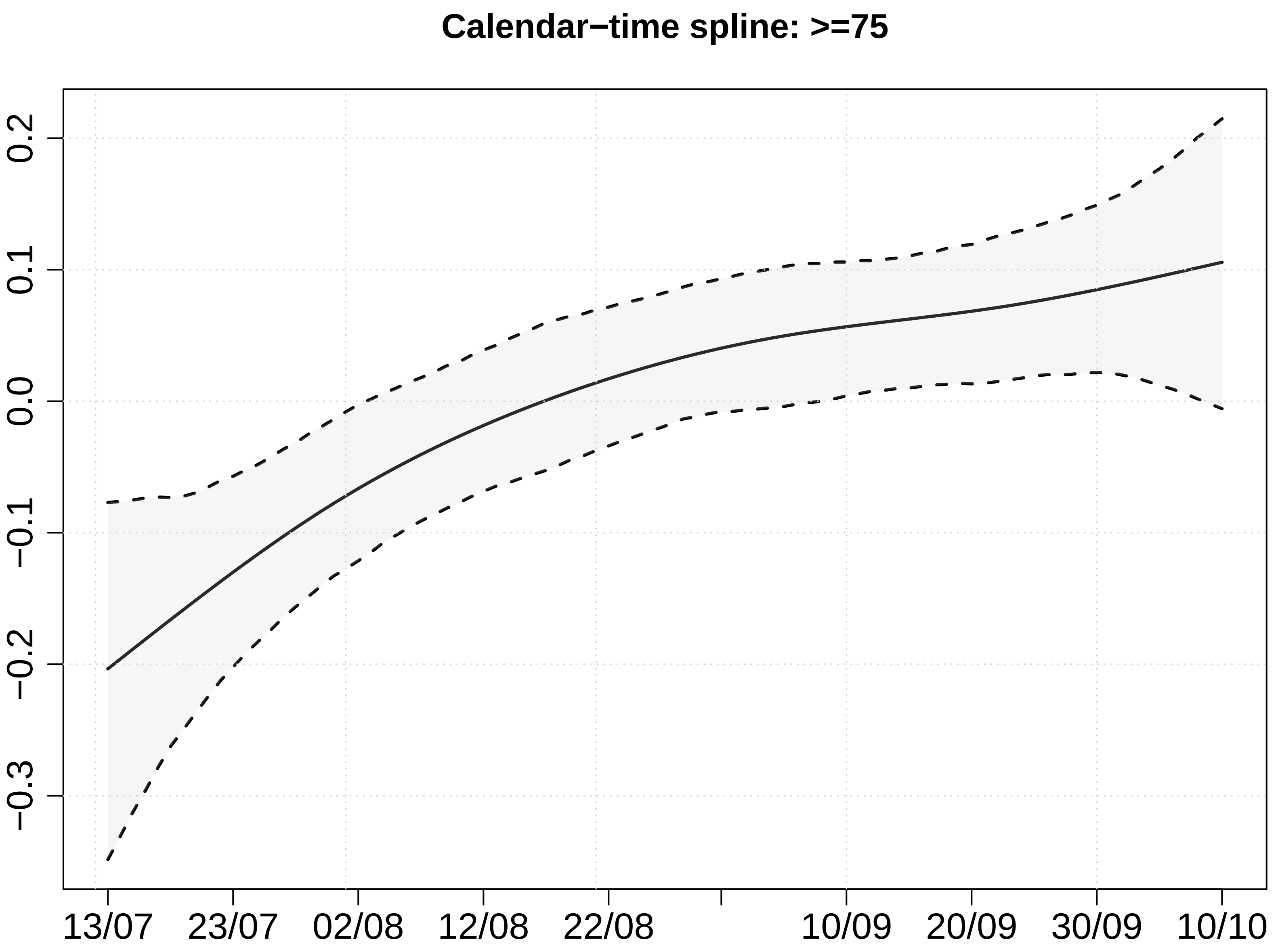
Calendar−time spline: >=75
0.2
0.1
0.0
−0.1
−0.2
−0.3
13/07
23/07
02/08
12/08
22/08
10/09
20/09
30/09
10/10

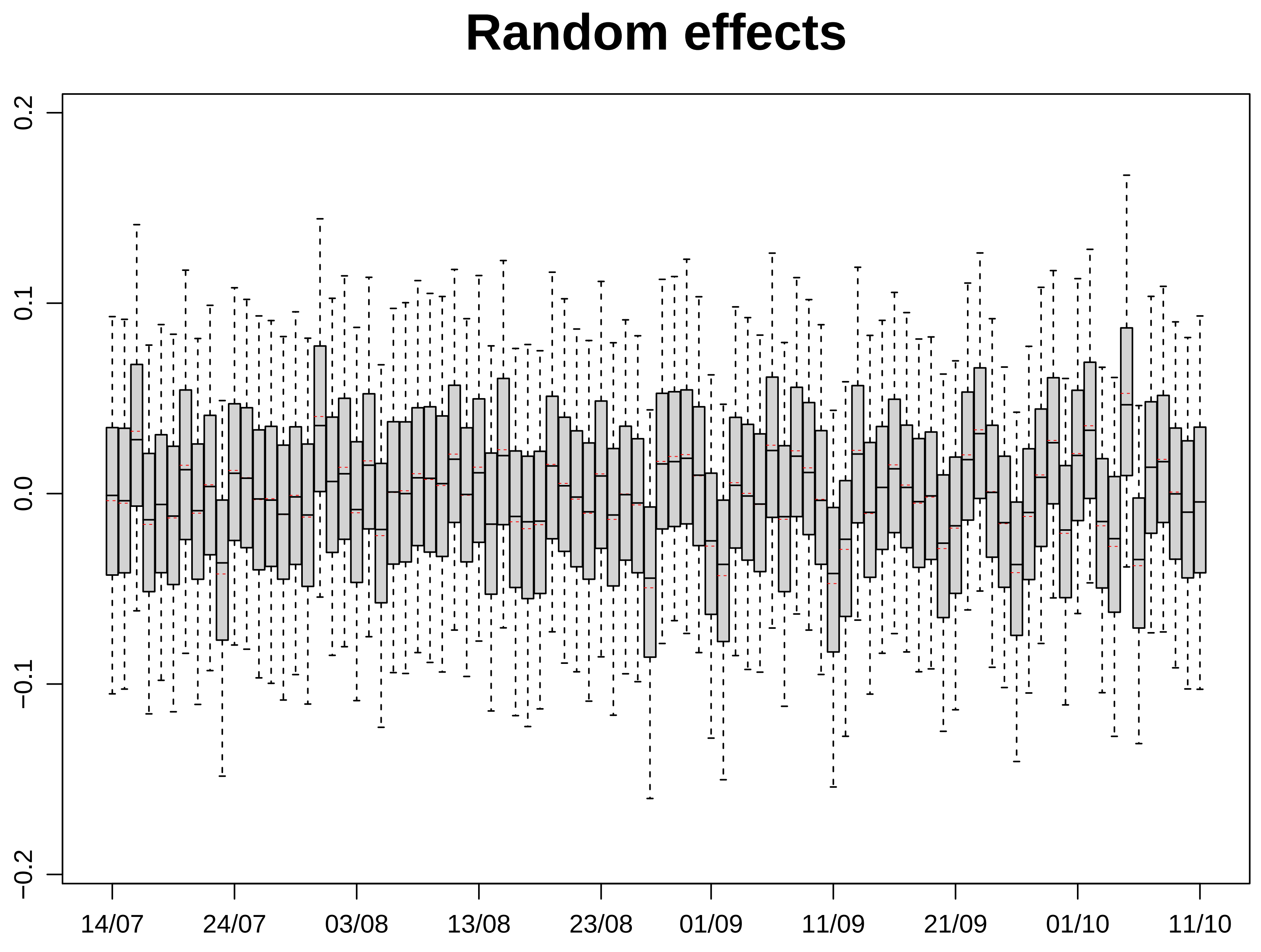

Random effects
0.2
0.1
0.0
−0.1
−0.2
14/07
24/07
03/08
13/08
23/08
01/09
11/09
21/09
01/10
11/10

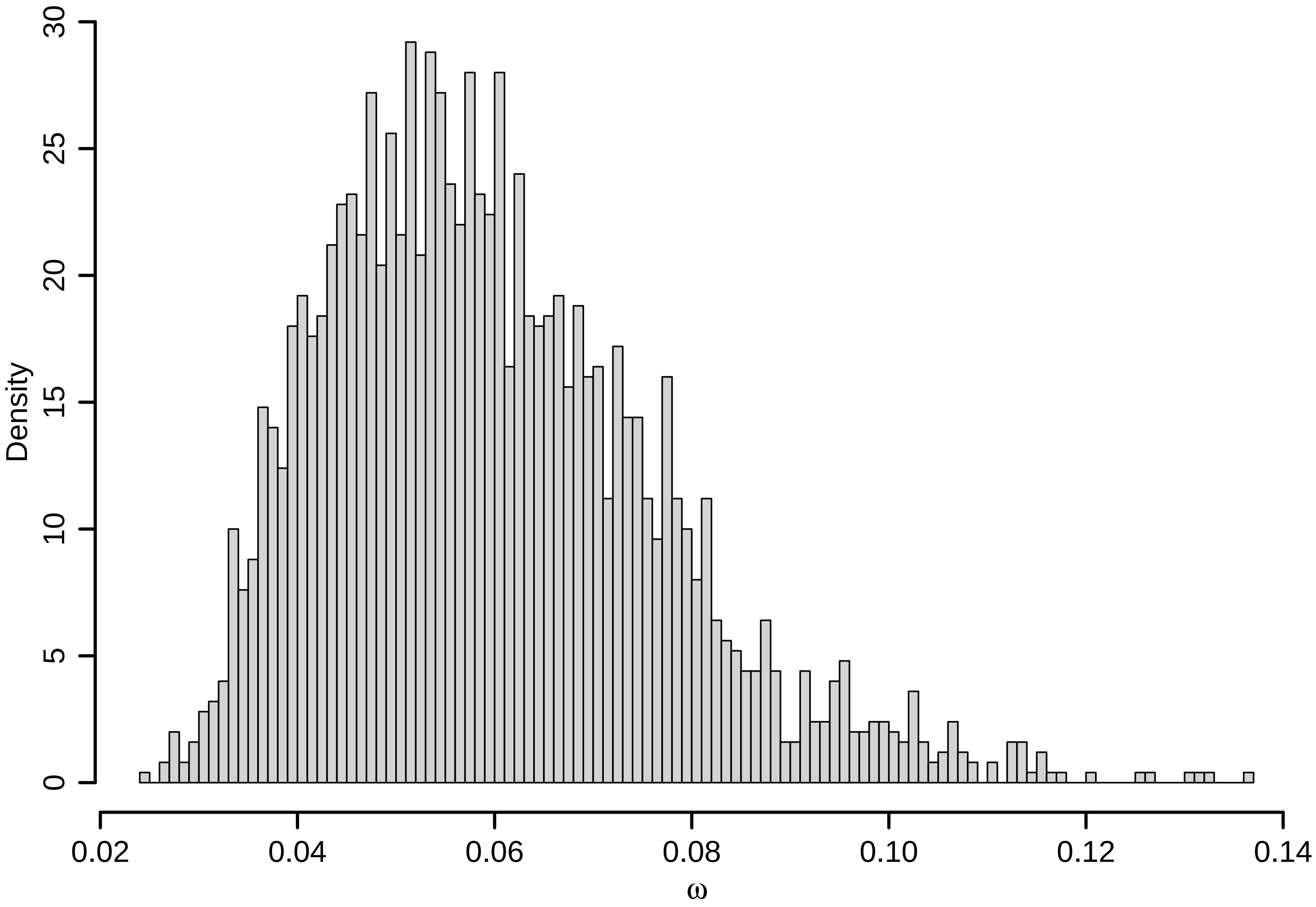

Density
30
25
20
15
10
5
0
0.02
0.04
0.06
0.08
0.10
0.12
0.14
ω

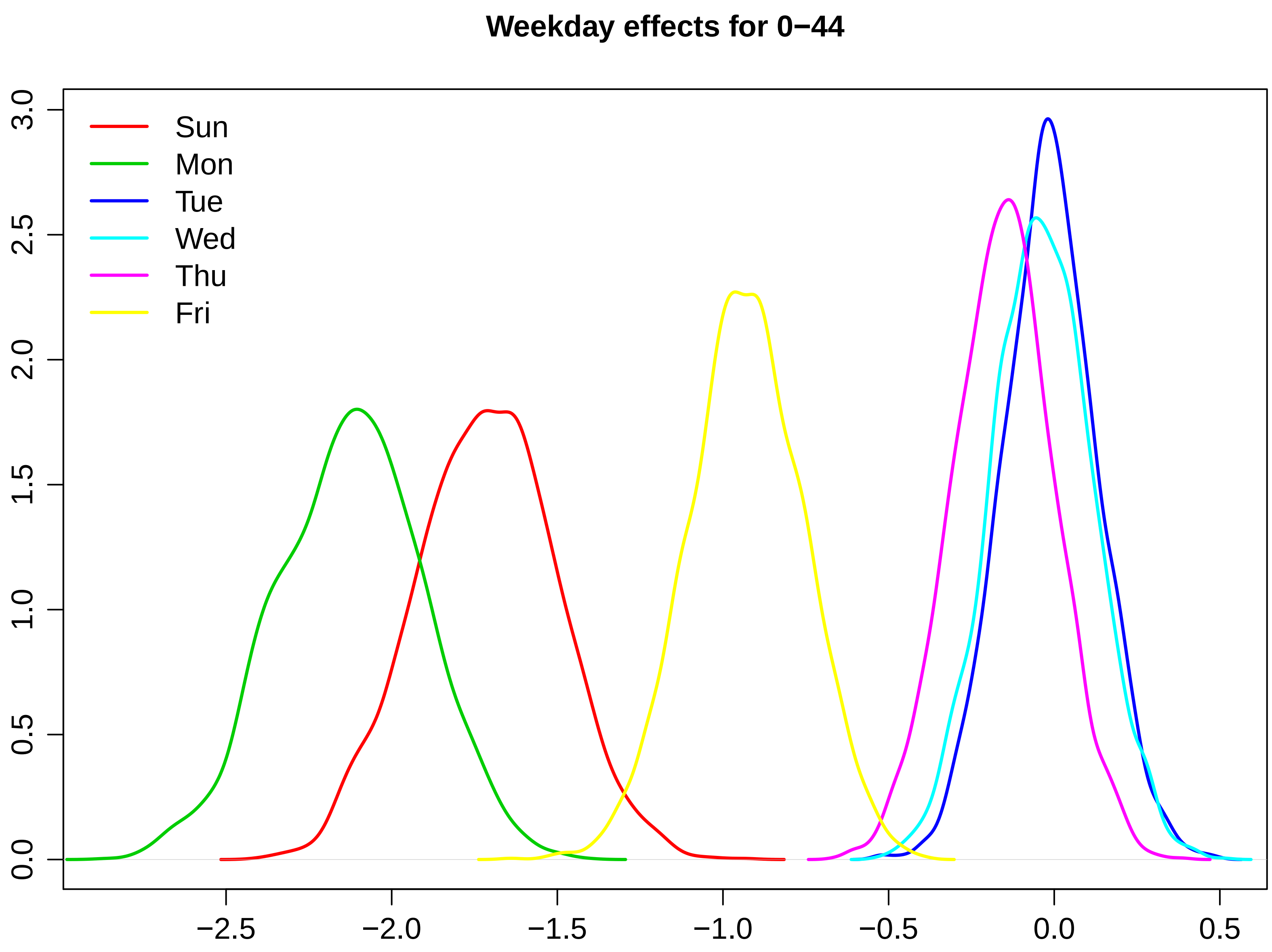

Weekday effects for 0−44
Sun
Mon
Tue
Wed
Thu
Fri
0.0
0.5
1.0
1.5
2.0
2.5
3.0
−2.5
−2.0
−1.5
−1.0
−0.5
0.0
0.5

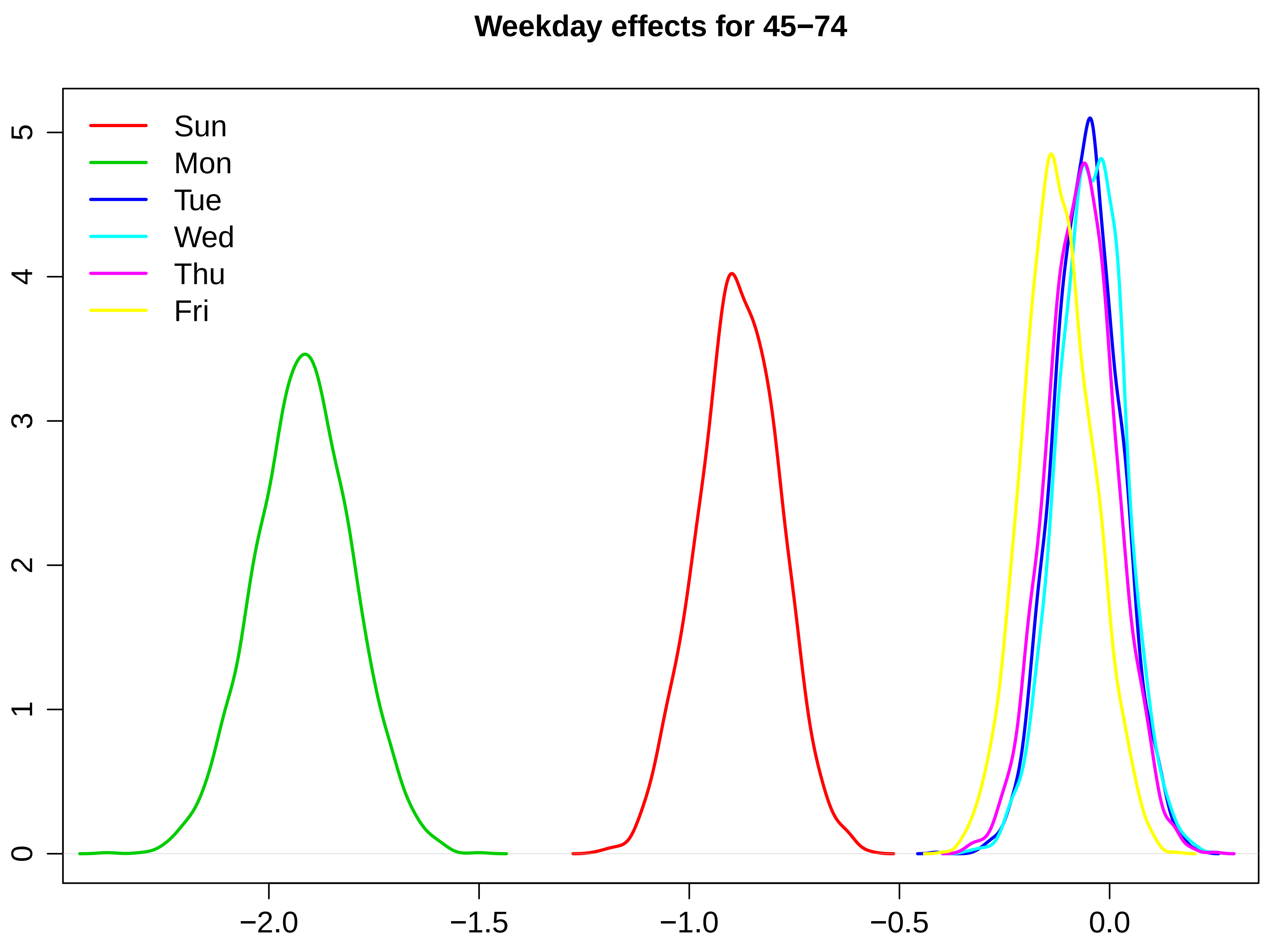
Weekday effects for 45−74
Sun
Mon
Tue
Wed
Thu
Fri
0
1
2
3
4
5
−2.0
−1.5
−1.0
−0.5
0.0

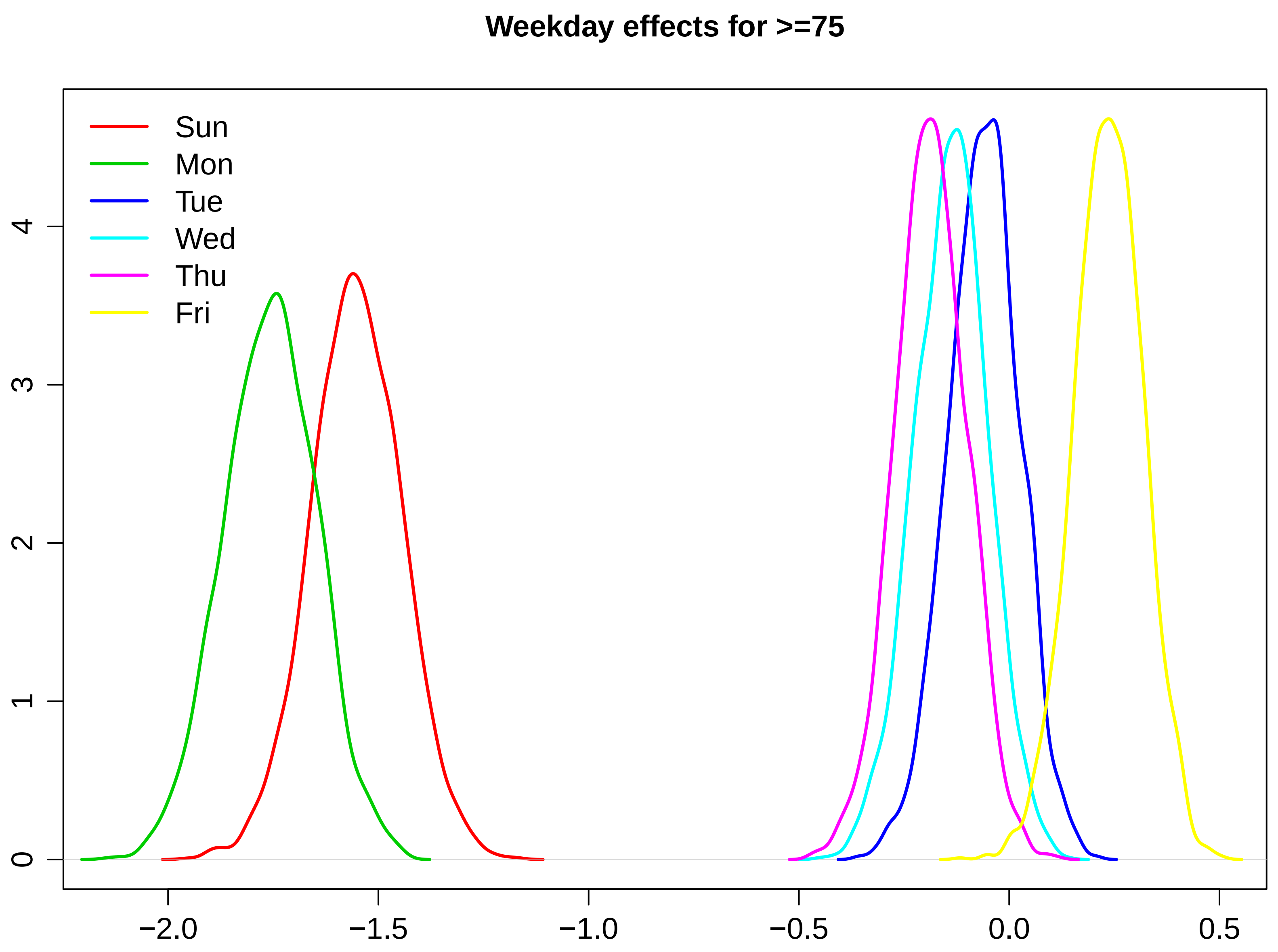
Weekday effects for >=75
Sun
Mon
Tue
Wed
Thu
Fri
0
1
2
3
4
−2.0
−1.5
−1.0
−0.5
0.0
0.5

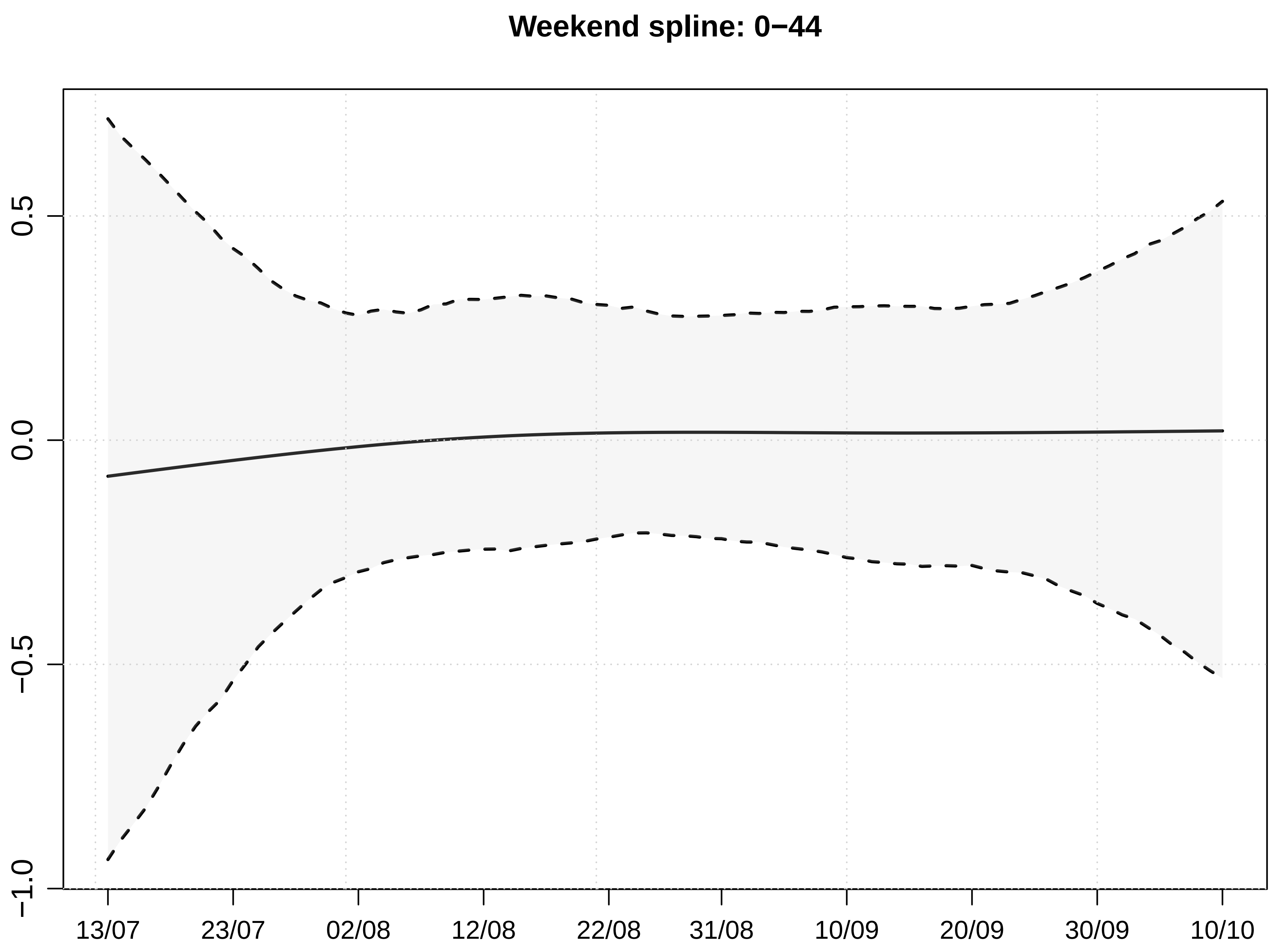
Weekend spline: 0−44
0.5
0.0
−0.5
−1.0
13/07
23/07
02/08
12/08
22/08
31/08
10/09
20/09
30/09
10/10

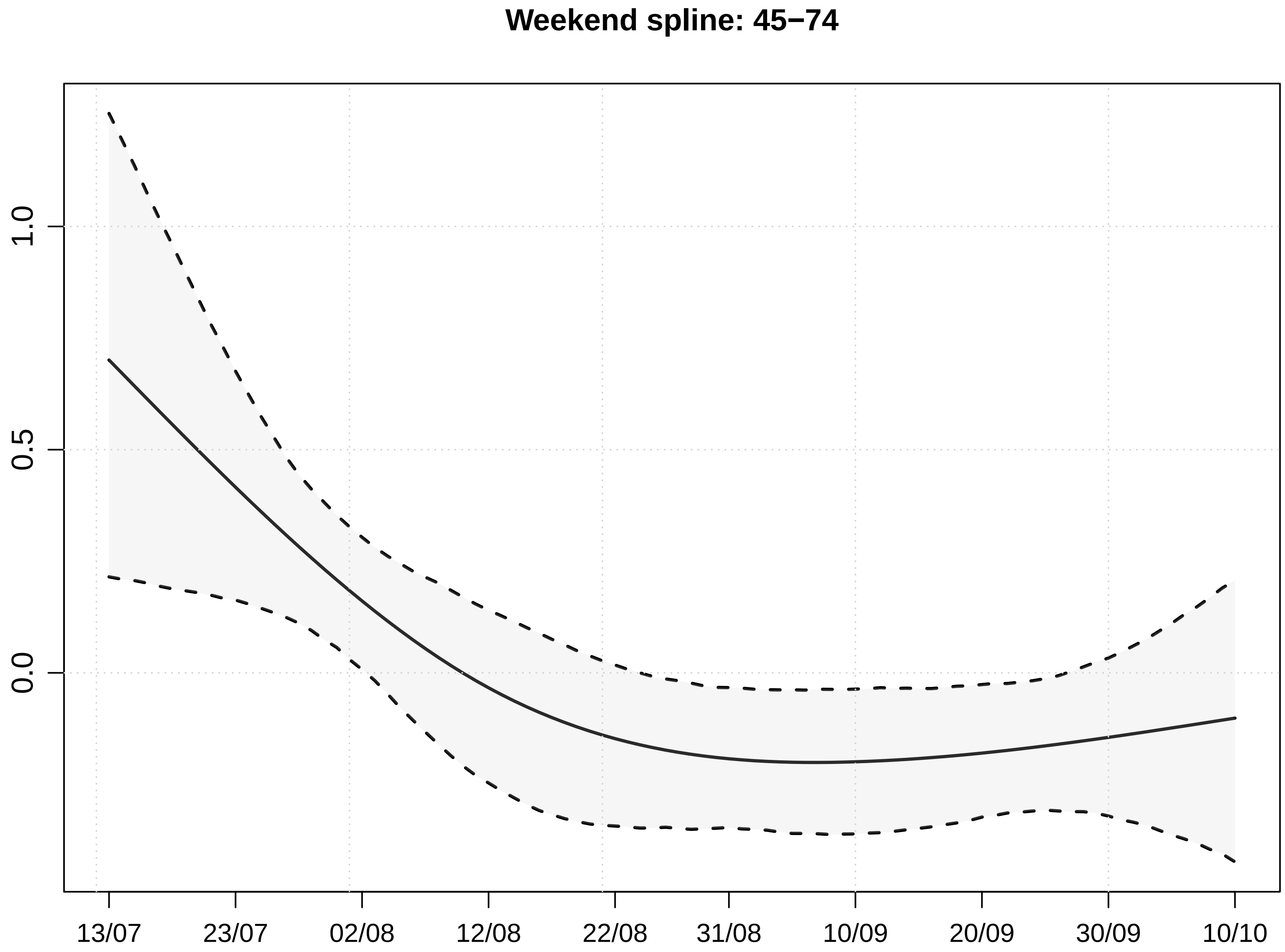

Weekend spline: 45−74
1.0
0.5
0.0
13/07
23/07
02/08
12/08
22/08
31/08
10/09
20/09
30/09
10/10

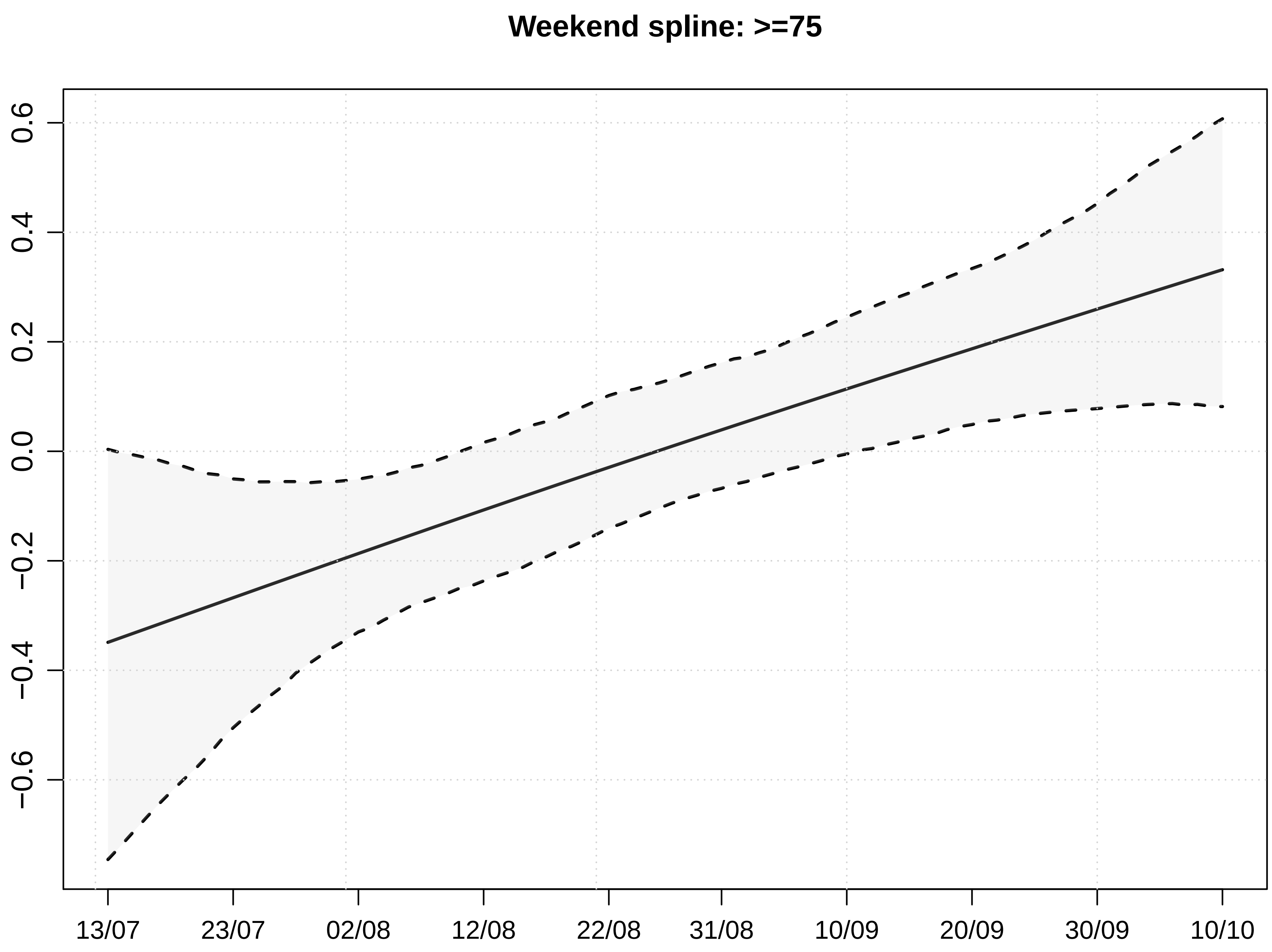

Weekend spline: >=75
0.6
0.4
0.2
0.0
−0.2
−0.4
−0.6
13/07
23/07
02/08
12/08
22/08
31/08
10/09
20/09
30/09
10/10

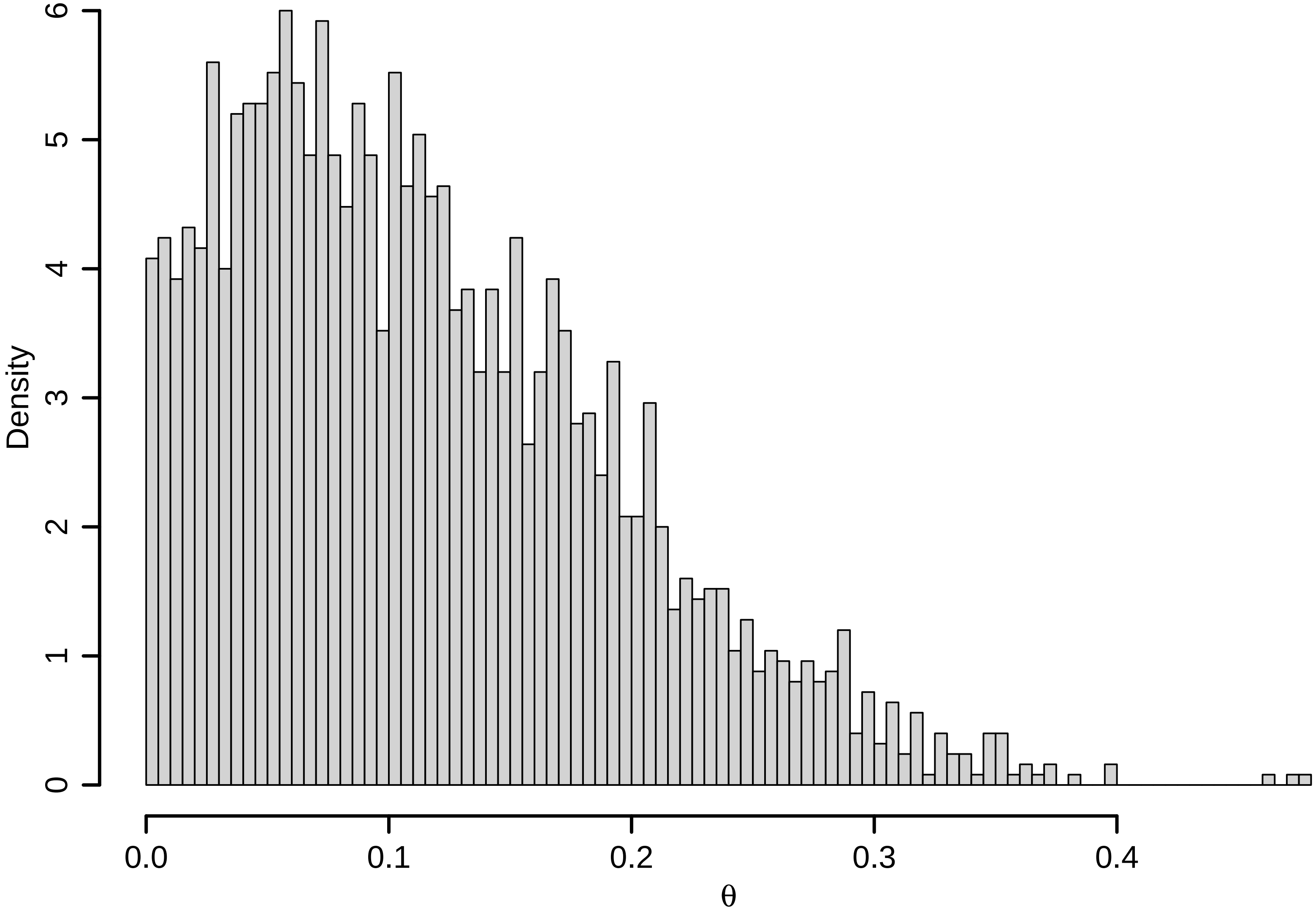

Density
0
1
2
3
4
5
6
0.0
0.1
0.2
0.3
0.4
θ

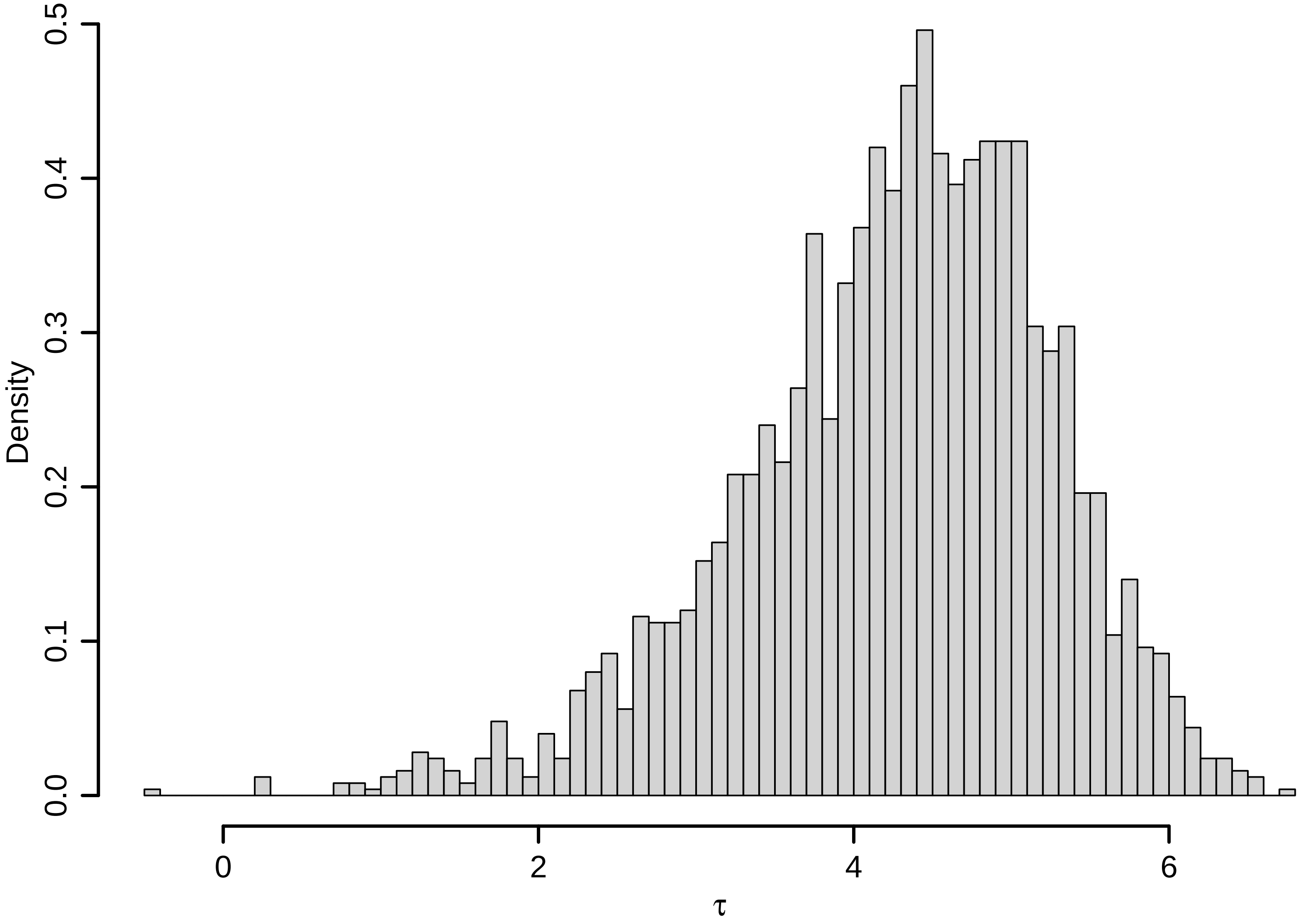

Density
0.0
0.1
0.2
0.3
0.4
0.5
0
2
4
6
τ

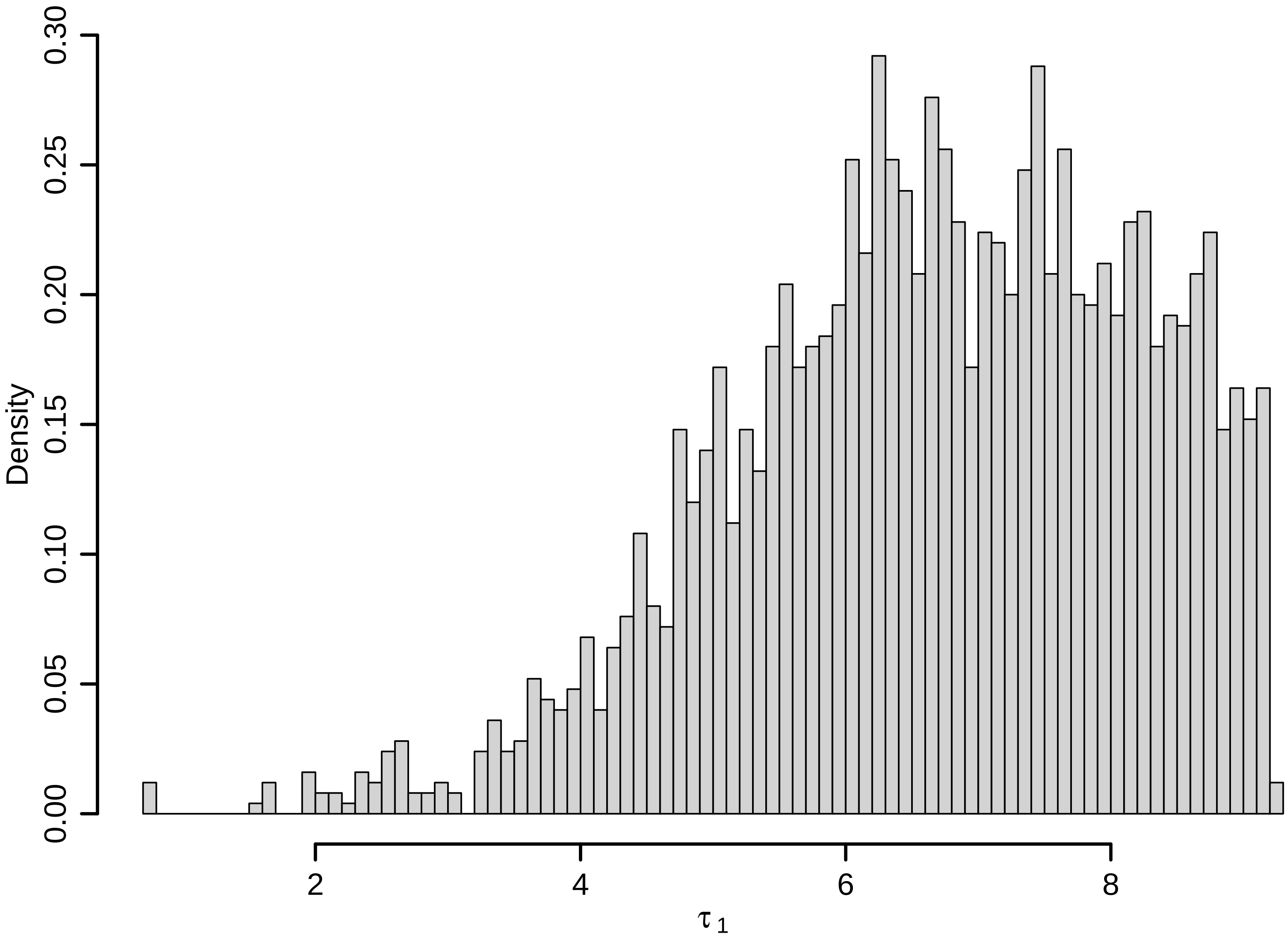
Density
0.00
0.05
0.10
0.15
0.20
0.25
0.30
2
4
6
8
τ_1

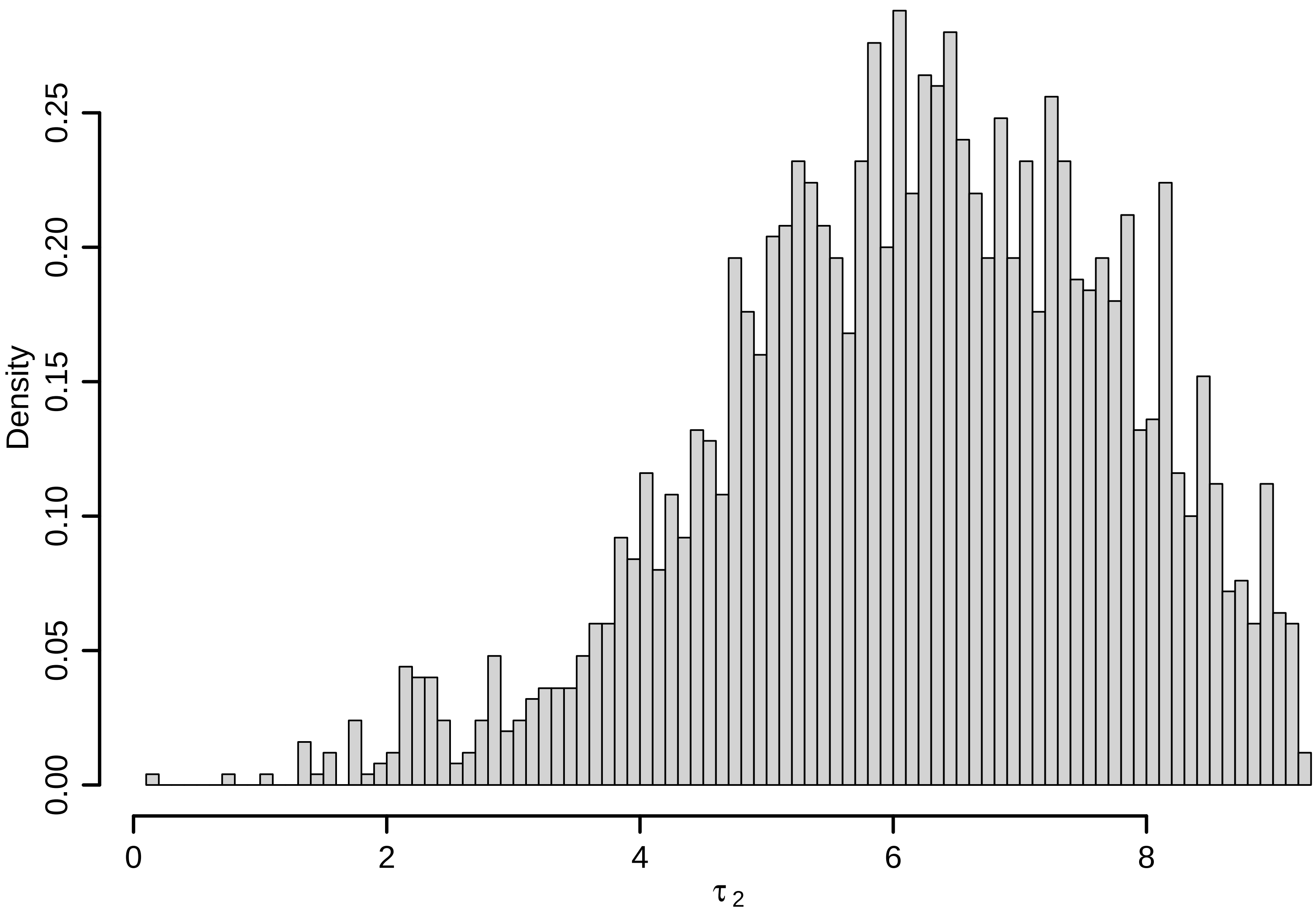

Density
0.00
0.05
0.10
0.15
0.20
0.25
0
2
4
6
8
τ 2

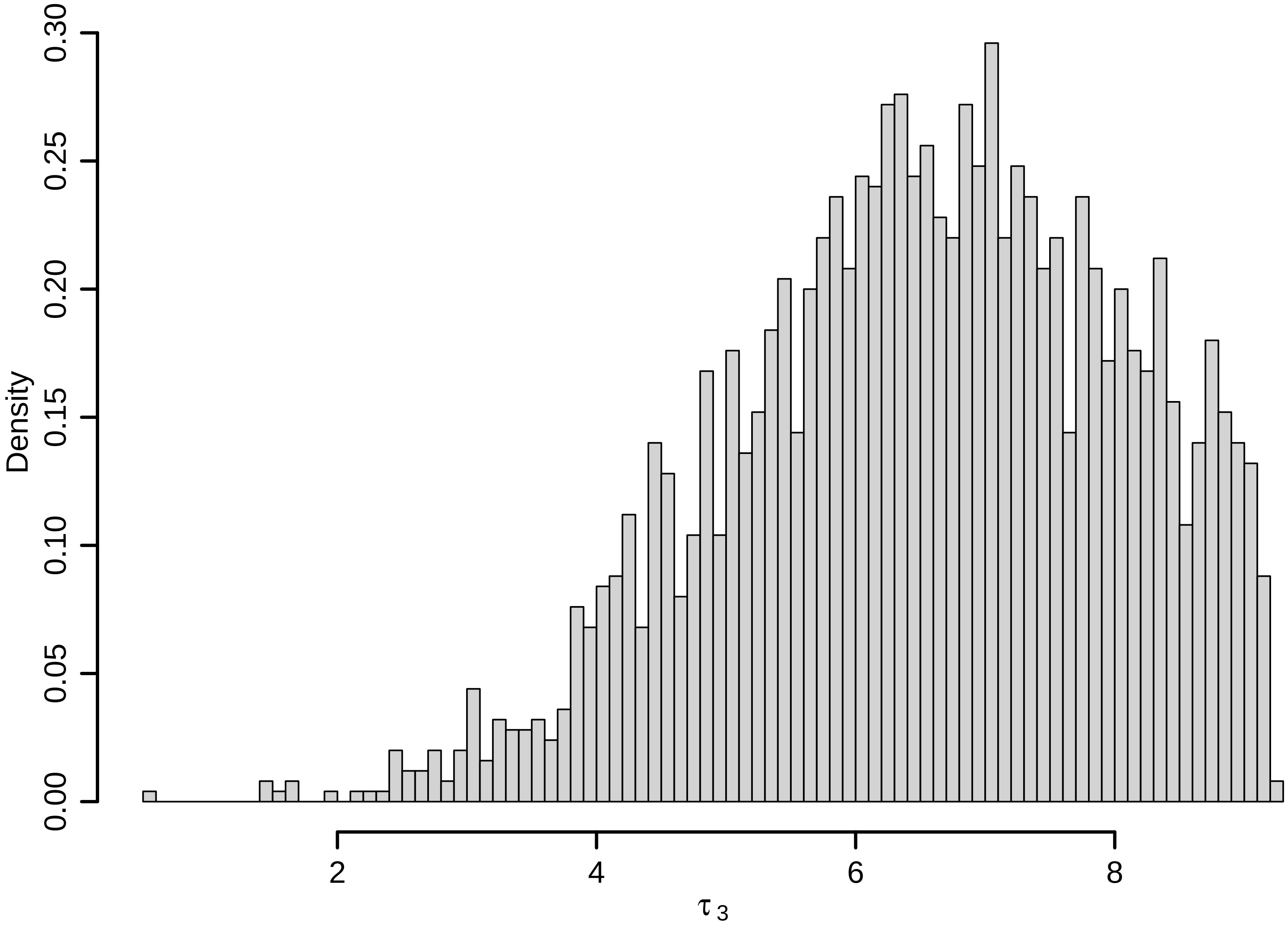

Density
0.00
0.05
0.10
0.15
0.20
0.25
0.30
2
4
6
8
τ_3

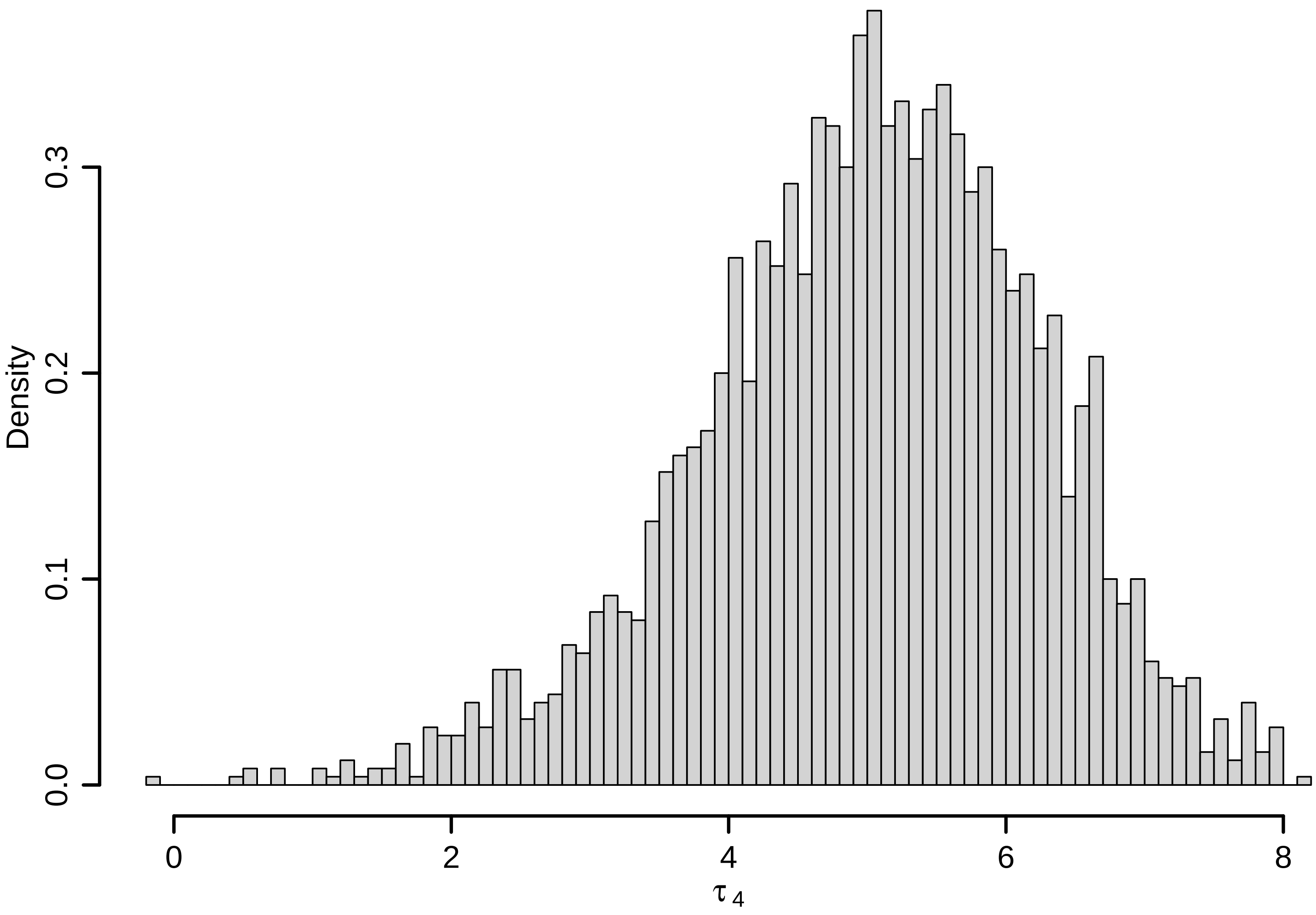

Density
0.0
0.1
0.2
0.3
0
2
4
6
8
τ_4

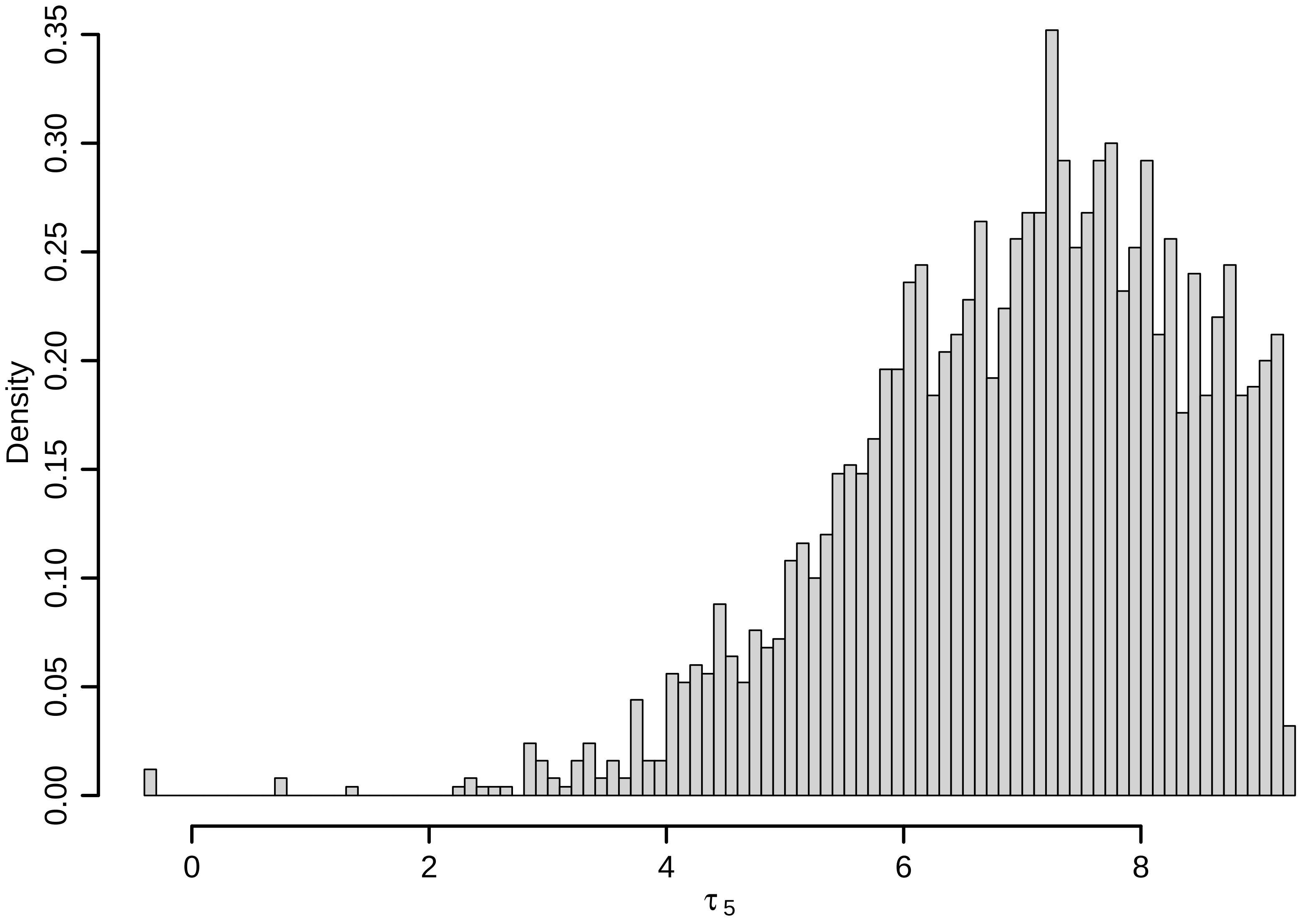
Density
0.00
0.05
0.10
0.15
0.20
0.25
0.30
0.35
0
2
4
6
8
τ_5

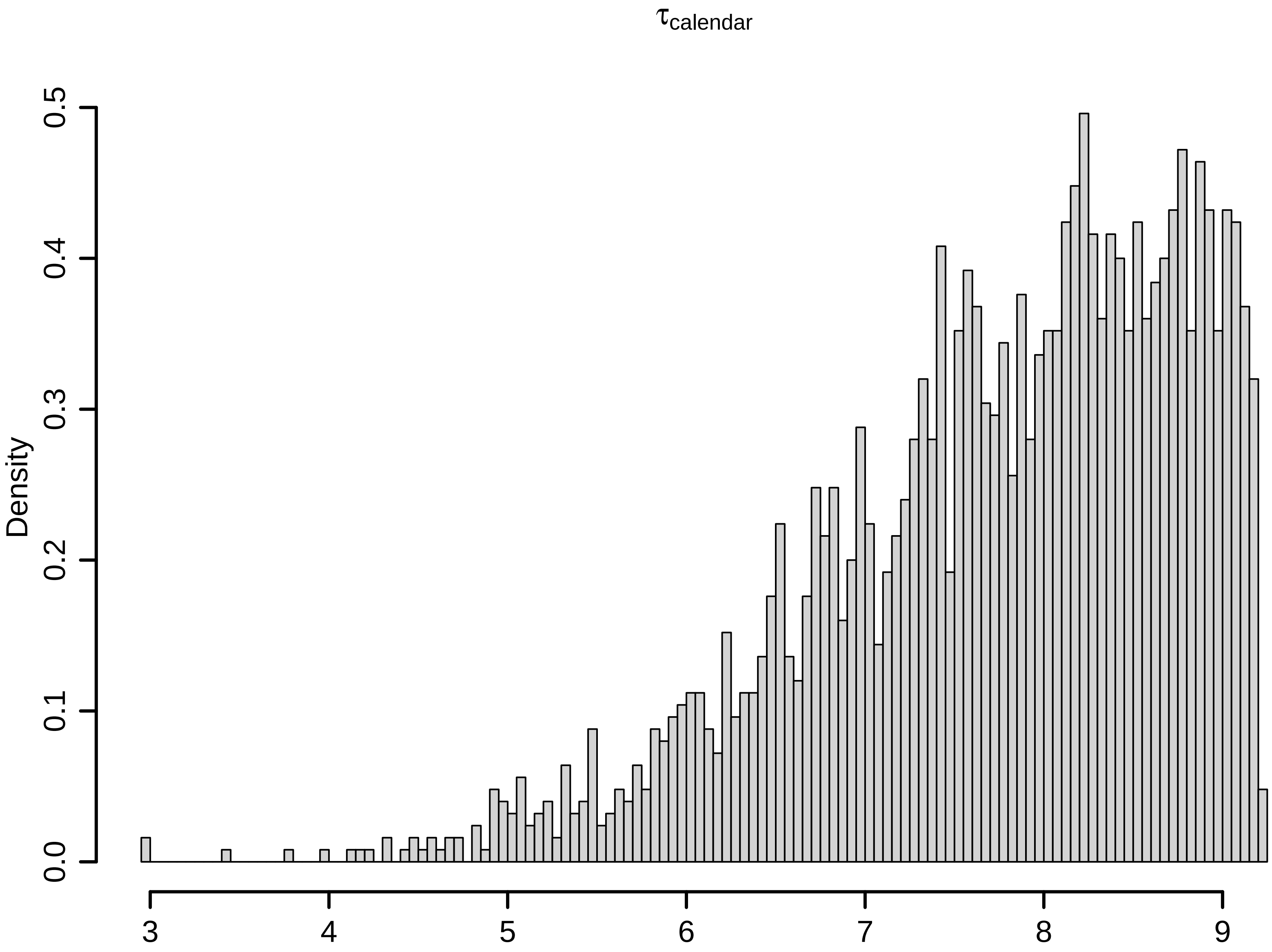
$\tau_{calendar}$
Density
0.0
0.1
0.2
0.3
0.4
0.5
3
4
5
6
7
8
9

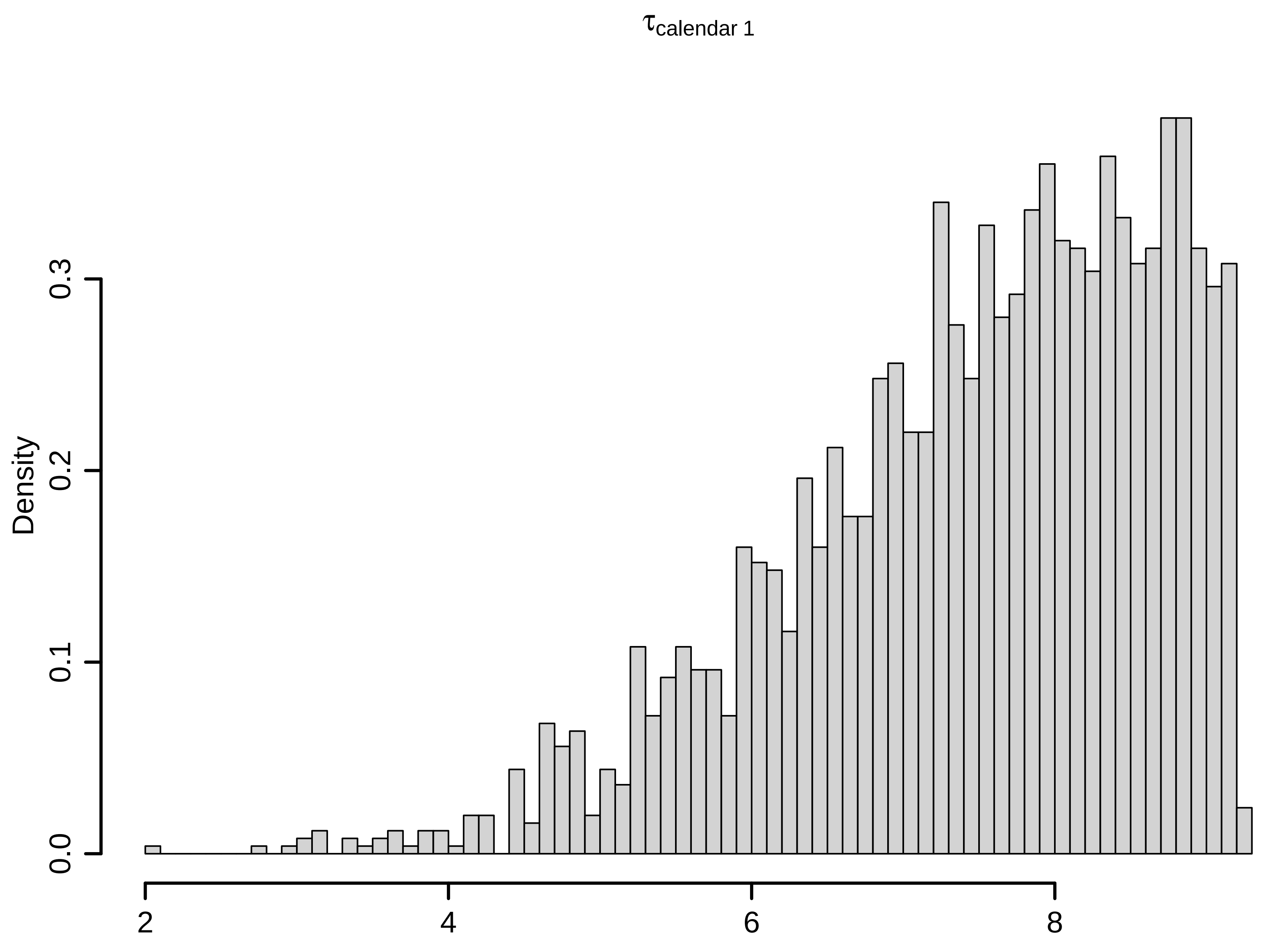

τcalendar 1
Density
0.0
0.1
0.2
0.3
2
4
6
8

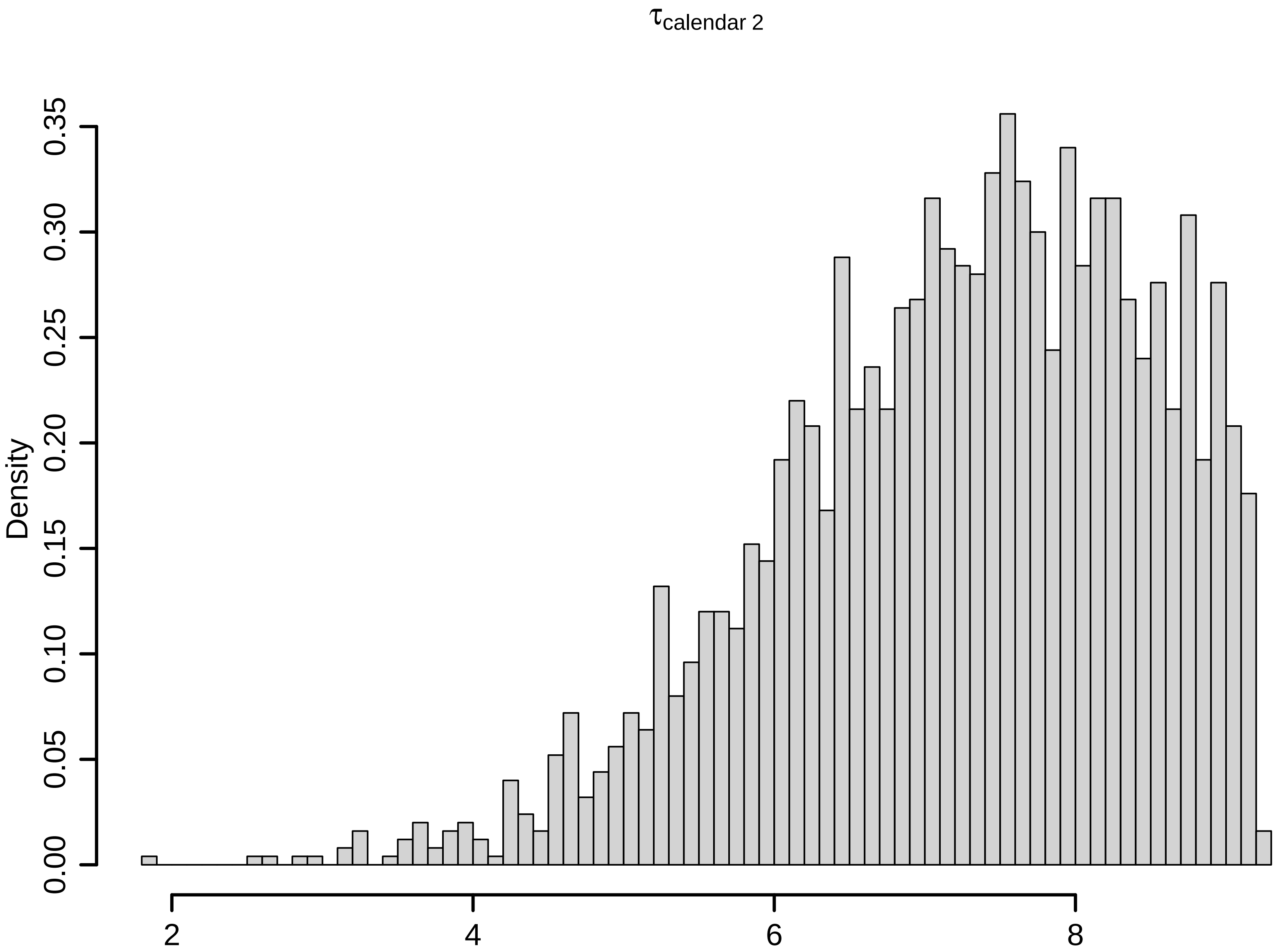
$\tau_{calendar\ 2}$
Density
0.00
0.05
0.10
0.15
0.20
0.25
0.30
0.35
2
4
6
8

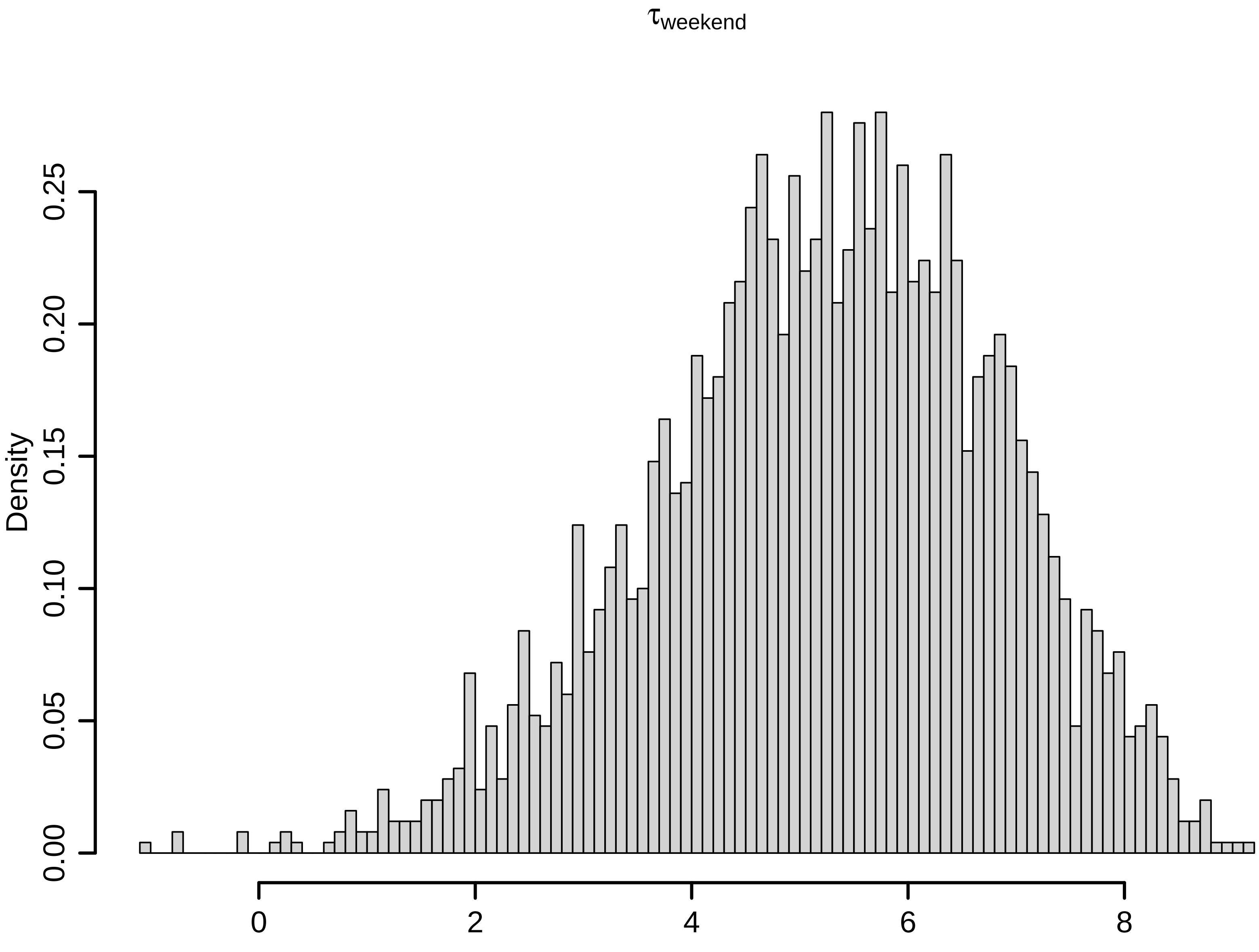

$\tau_{weekend}$
Density
0.00
0.05
0.10
0.15
0.20
0.25
0
2
4
6
8

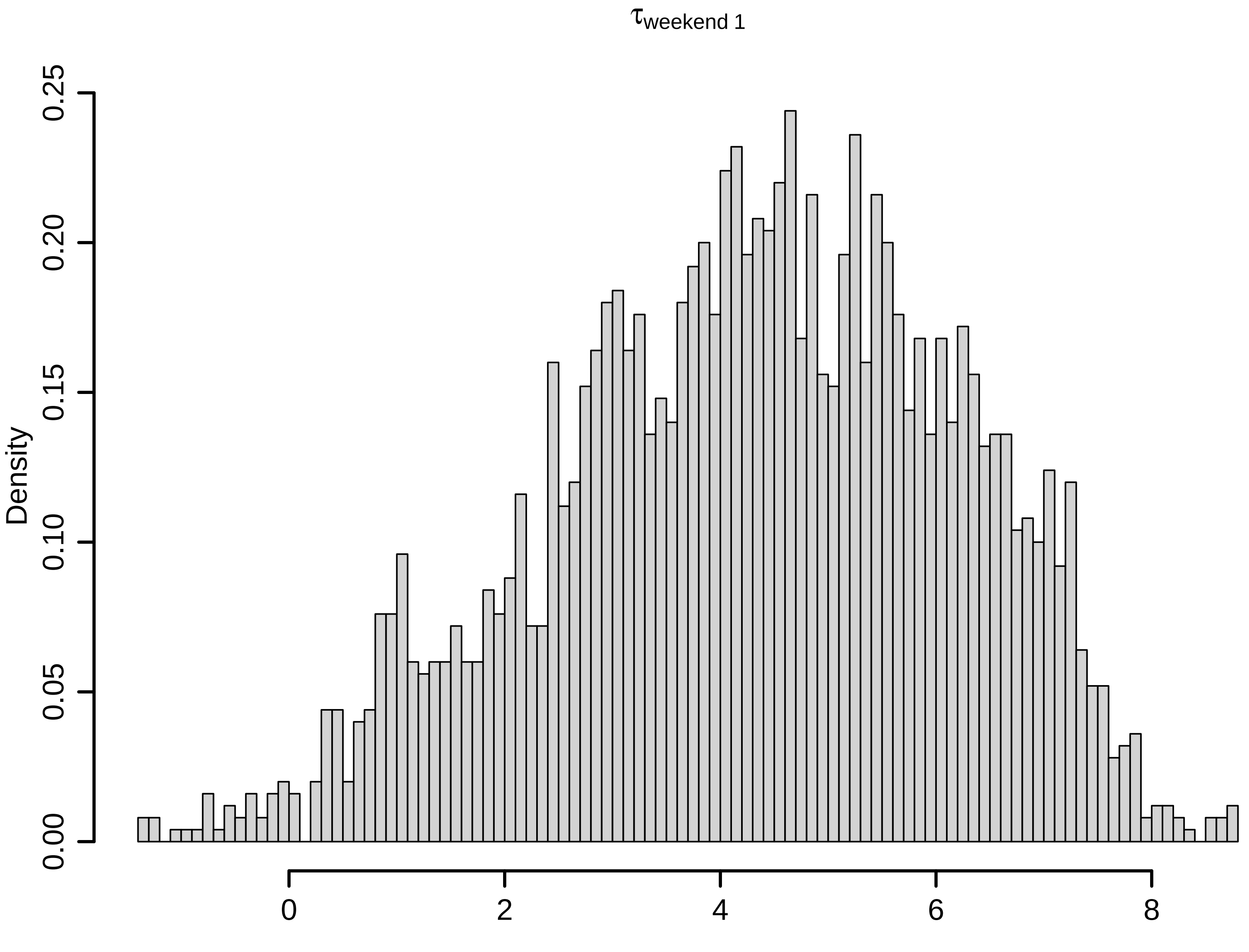

τweekend 1
Density
0.00
0.05
0.10
0.15
0.20
0.25
0
2
4
6
8

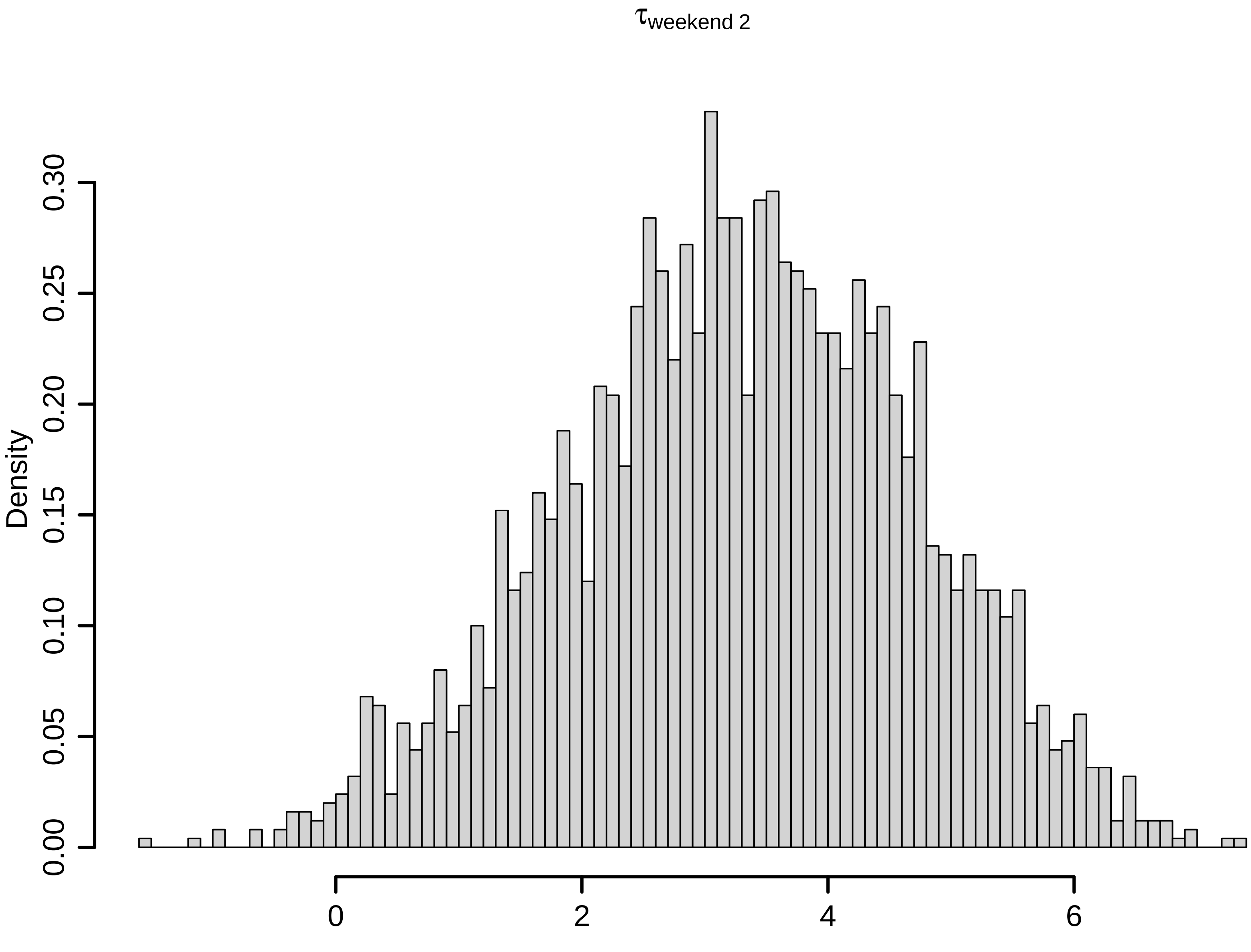

$\tau_{weekend\ 2}$
Density
0.00
0.05
0.10
0.15
0.20
0.25
0.30
0
2
4
6

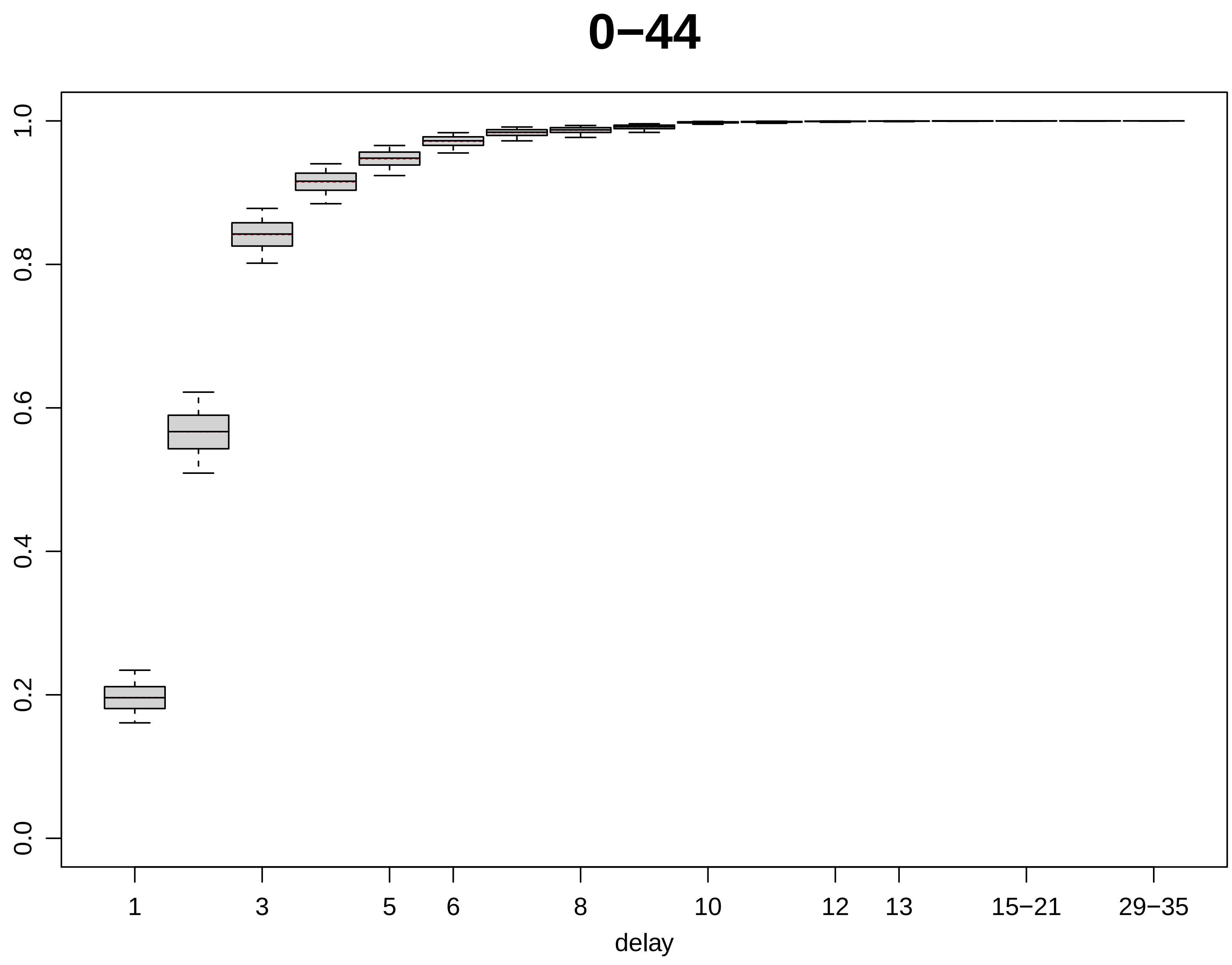

0−44
1.0
0.8
0.6
0.4
0.2
0.0
1
3
5
6
8
10
12
13
15−21
29−35
delay

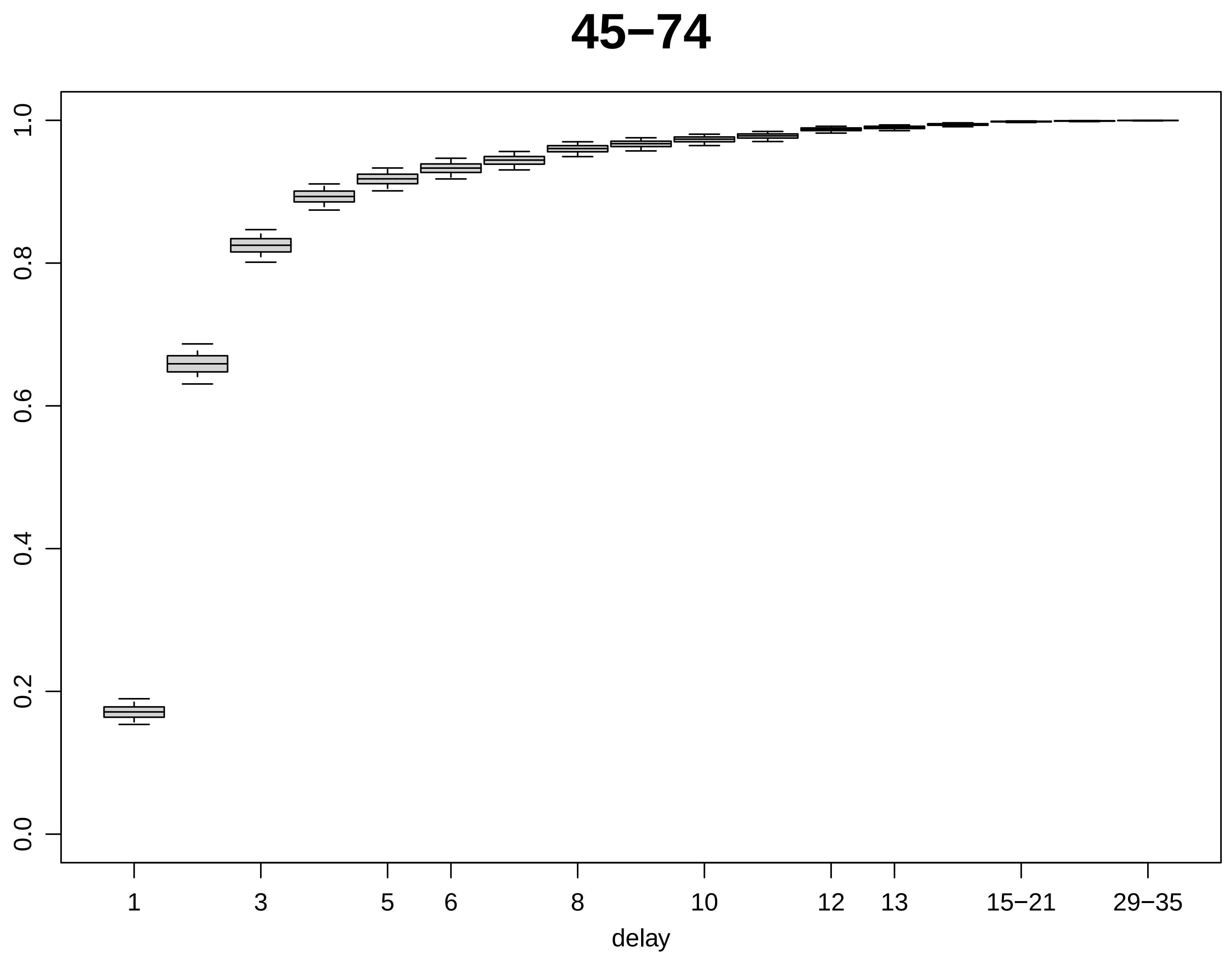
45−74
1.0
0.8
0.6
0.4
0.2
0.0
1
3
5
6
8
10
12
13
15−21
29−35
delay

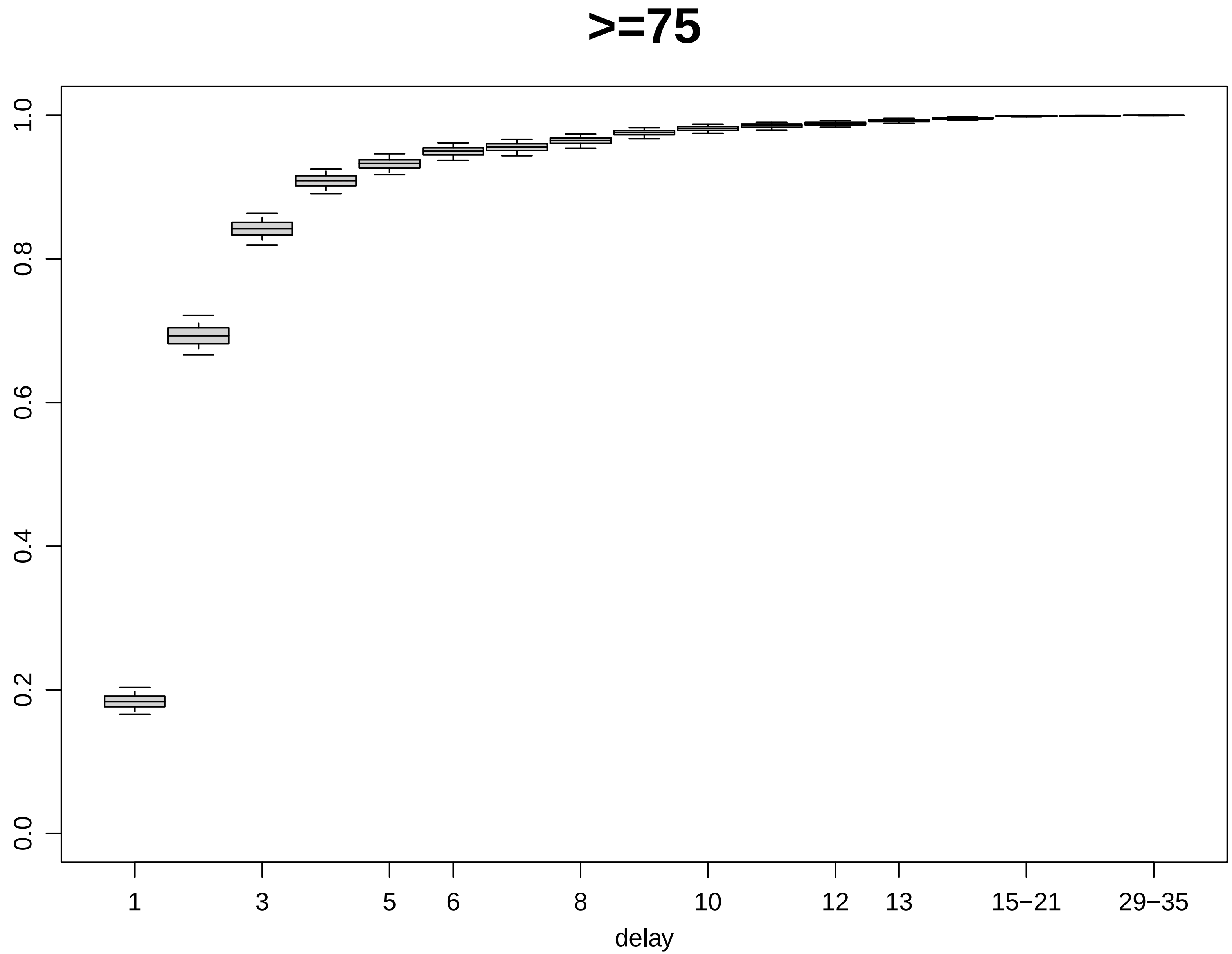

>=75
1.0
0.8
0.6
0.4
0.2
0.0
1
3
5
6
8
10
12
13
15−21
29−35
delay

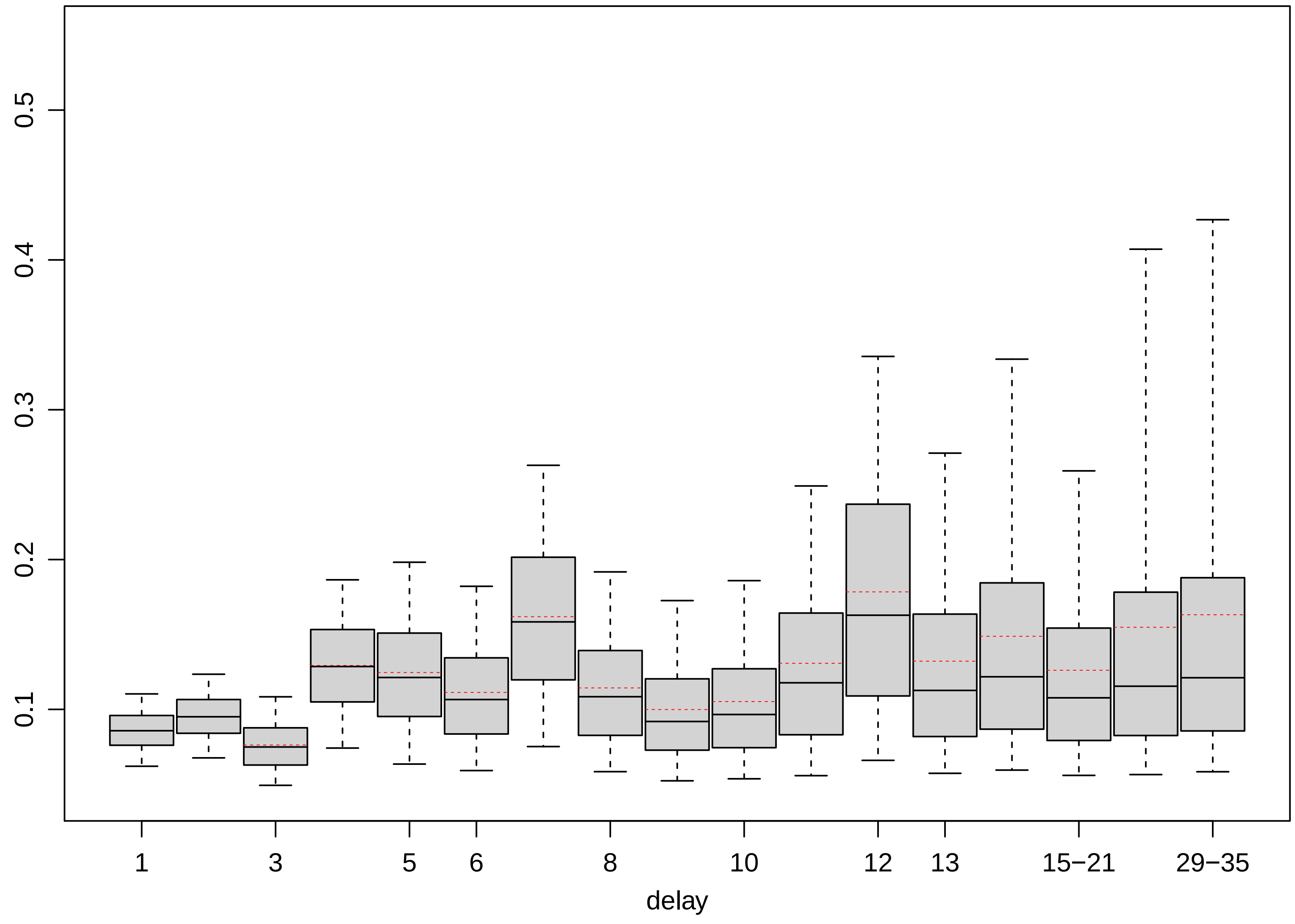
0.1
0.2
0.3
0.4
0.5
1
3
5
6
8
10
12
13
15−21
29−35
delay
