# Supplementary material for: Nowcasting COVID‐19 deaths in England by age and region
Source: J R Stat Soc Ser C Appl Stat. 2022 Jun 15:10.1111/rssc.12576. Online ahead of print. doi: 10.1111/rssc.12576 (PMC9349735; doi:10.1111/rssc.12576)
Supplement: Supplementary file 2 [file RSSC-9999-0-s001.pdf]

Supplementary Materials for "Nowcasting CoVID-19 Deaths in England by Age and Region"

Shaun Seaman, Pantelis Samartsidis, Meaghan Kall and Daniela De Angelis

S1 Likelihood of second adapted model in Section 2.3.4

$$
\begin{aligned}
& p\left(\{Z_{std} : s \in \mathcal{S}, 0 \leq t \leq D\} \mid \{Y_{st} : s \in \mathcal{S}\}, \alpha_{\bullet t}, \beta_{\bullet t}\right) \\
& \propto p\left(\{Z_{std} : s \in \mathcal{S}, 0 \leq t \leq D_t\} \mid \{Y_{st} : s \in \mathcal{S}\}, \alpha_{\bullet t}, \beta_{\bullet t}\right) \quad (1) \\
& = p\left(\{Z_{std} : s \in \mathcal{S}, 0 \leq t \leq D_t\} \mid \{Y_{st}, W_{st} : s \in \mathcal{S}\}, \{Z_{\bullet td} : 0 \leq d \leq D_t\}, \alpha_{\bullet t}, \beta_{\bullet t}\right) \\
& \quad \times p\left(\{W_{st} : s \in \mathcal{S}\} \mid \{Y_{st} : s \in \mathcal{S}\}, \{Z_{\bullet td} : 0 \leq d \leq D_t\}, \alpha_{\bullet t}, \beta_{\bullet t}\right) \\
& \quad \times p\left(\{Z_{\bullet td} : d = 0, \ldots, D_t\} \mid \{Y_{st} : s \in \mathcal{S}\}, \alpha_{\bullet t}, \beta_{\bullet t}\right) \\
& = p\left(\{Z_{std} : s \in \mathcal{S}, 0 \leq t \leq D_t\} \mid \{W_{st} : s \in \mathcal{S}\}, \{Z_{\bullet td} : 0 \leq d \leq D_t\}\right) \\
& \quad \times p\left(\{W_{st} : s \in \mathcal{S}\} \mid \{Y_{st} : s \in \mathcal{S}\}, W_{\bullet t}\right) \\
& \quad \times p\left(\{Z_{\bullet td} : d = 0, \ldots, D_t\} \mid Y_{\bullet t}, \alpha_{\bullet t}, \beta_{\bullet t}\right) \quad (2)
\end{aligned}
$$

The proportionality sign in expression (1) reflects the fact that Z_{std} is unobserved when $d > D_t$, and so can be integrated out (as mentioned in Section 2.2). The first term in expression (2) is the likelihood of a generalised hypergeometric distribution (Mehra and Patel, 1983). It is a function only of observed variables, and hence can be ignored when calculating full-conditional distributions in the MCMC algorithm.

Reference

Mehra, CR and Patel, NR (1983). A Network Algorithm for Performing Fisher's Exact Test in $r \times c$ Contingency Tables. *Journal of the American Statistical Association*; **78**: 427–434.

S2 Additional figures referred to in our article

This section contains supplementary figures referred to in our article. These include a directed acyclic graph (DAG) for the (unadapted) model described in Section 2; graphs relating to the analysis of data from the whole of England data available on 29th June, described in Section 3; and an estimate of the delay distribution by weekday of death, described in Section 4.

Note that 'weekend' in the phrases 'weekend effect' and 'weekend spline' refers to Sunday and Monday.

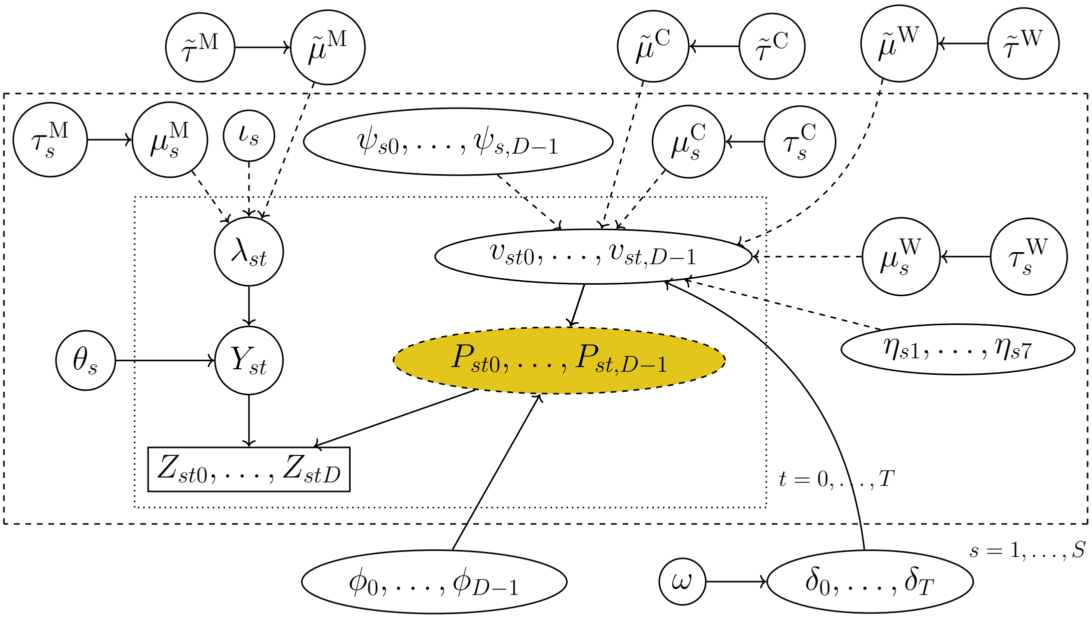

Figure S1: Directed acyclic graph for the model described in Sections 2.1–2.4. Circles and ellipses indicated unobserved variables. Solid and broken arrows indicate stochastic and deterministic links, respectively. Solid rectangles indicate observed variables. The yellow ellipse shows random variables that are integrated out in the likelihood function. Broken rectangles indicate replication, either over $t = 0, \ldots, T$ or over $s = 1, \ldots S$. Superscripts M, W and C on spline parameters $\tilde{\tau}$, τ_s, $\tilde{\mu}$ and μ_s represent 'deaths' ('M' for 'mortality'), 'weekend effect' and 'calendar-time effect', respectively.

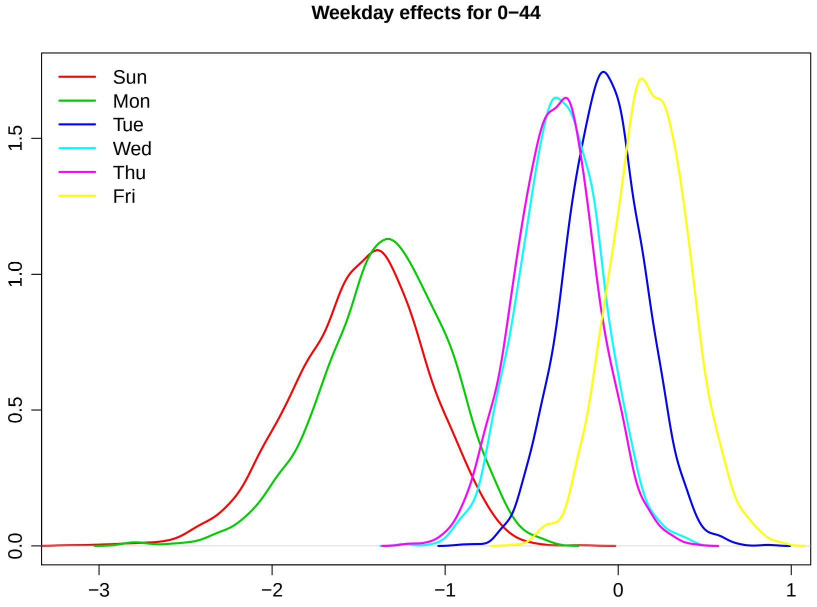

Figure S2: Posterior distributions of day of the week effects (η_j) in the 0–44 age stratum

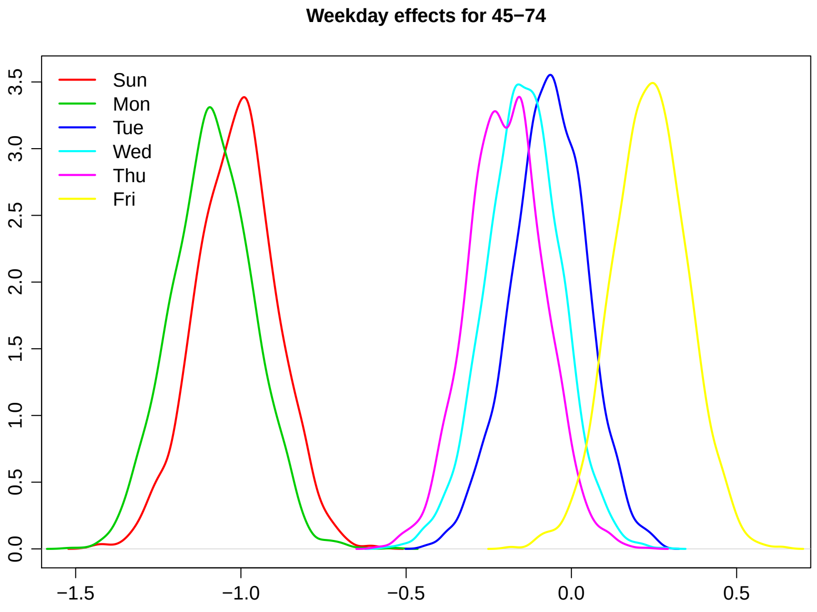

Figure S3: Posterior distributions of day of the week effects (η_j) in the three 45–74 age strata

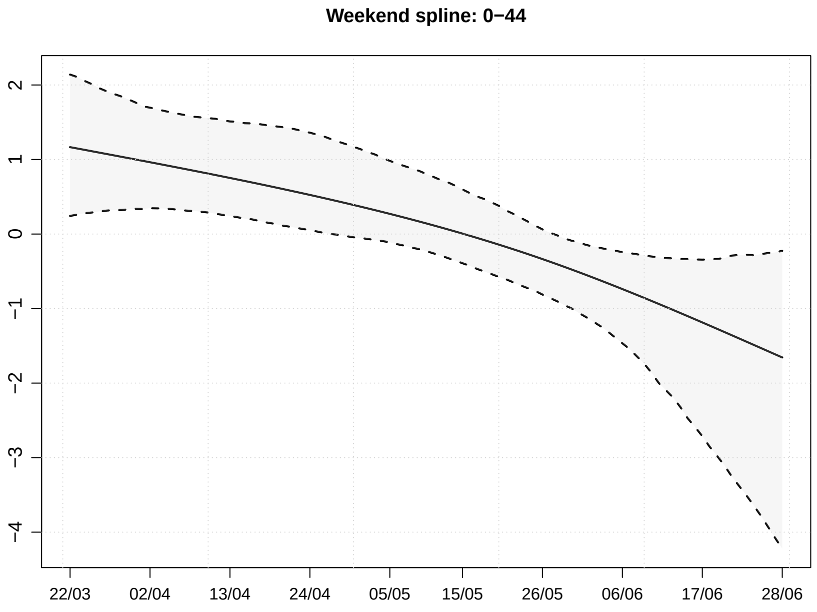

Figure S4: Posterior mean and 95% CI of the change in the weekend effect (g_{st}^W) in the 0–44 age stratum

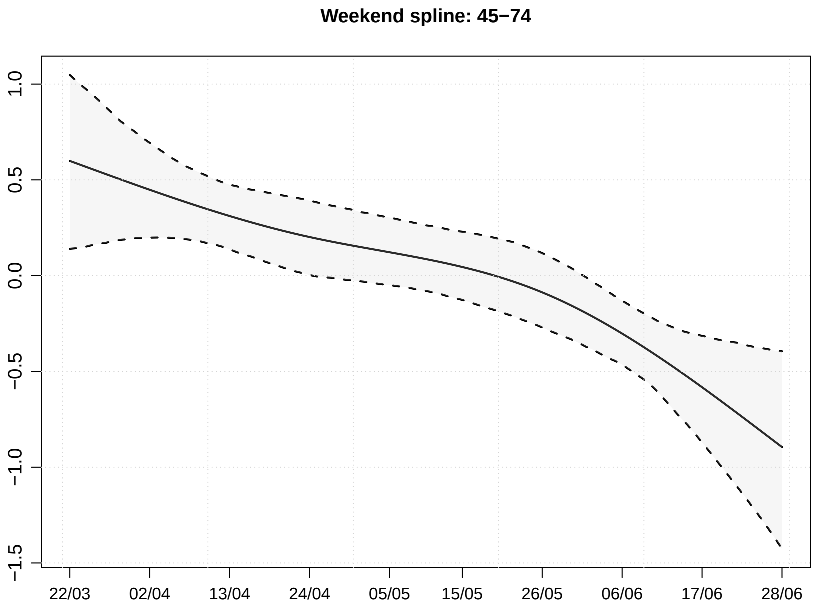

Figure S5: Posterior mean and 95% CI of the change in the weekend effect (g^W_{st}) in the three 45–74 age strata

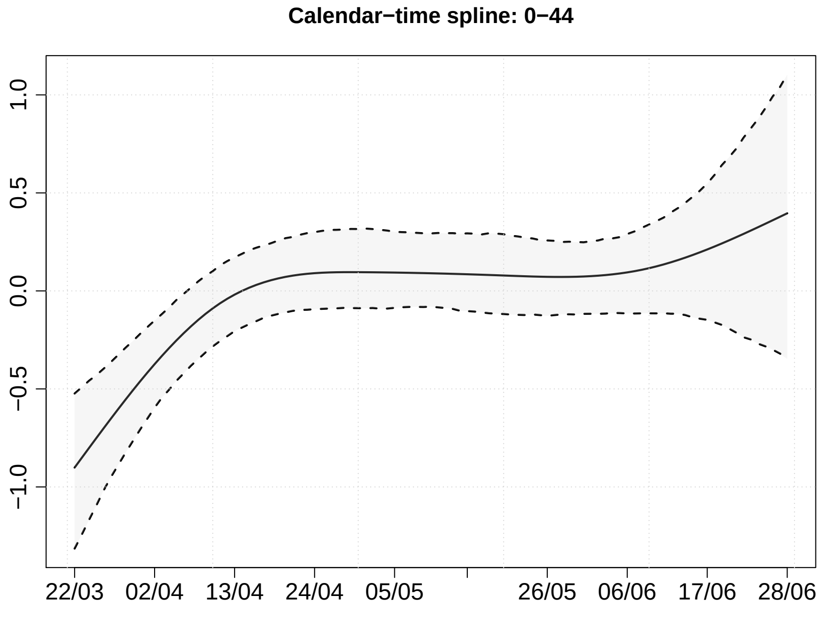

Figure S6: Posterior mean and 95% CI of the calendar-time effect (g^{C}_{st}) in the 0–44 age stratum

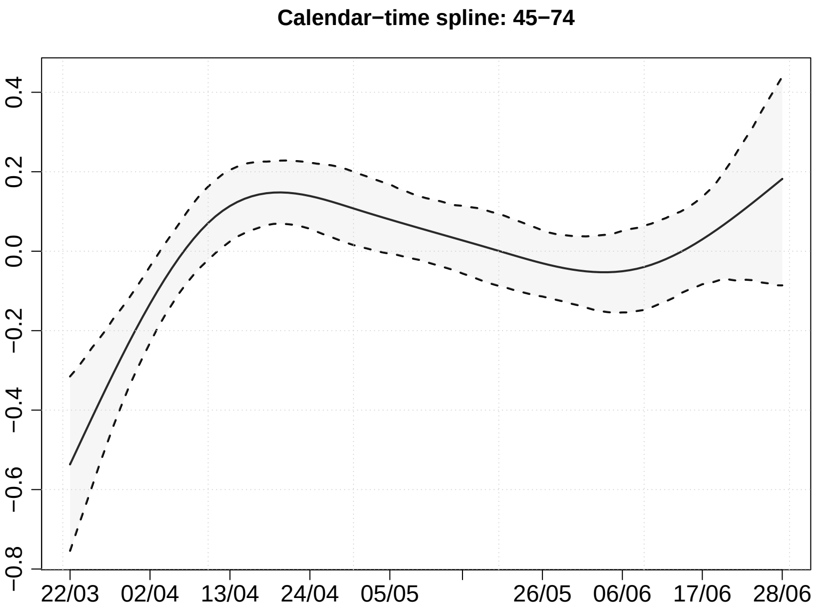

Figure S7: Posterior mean and 95% CI of the calendar-time effect (g^{C}_{st}) in the three 45–74 age strata

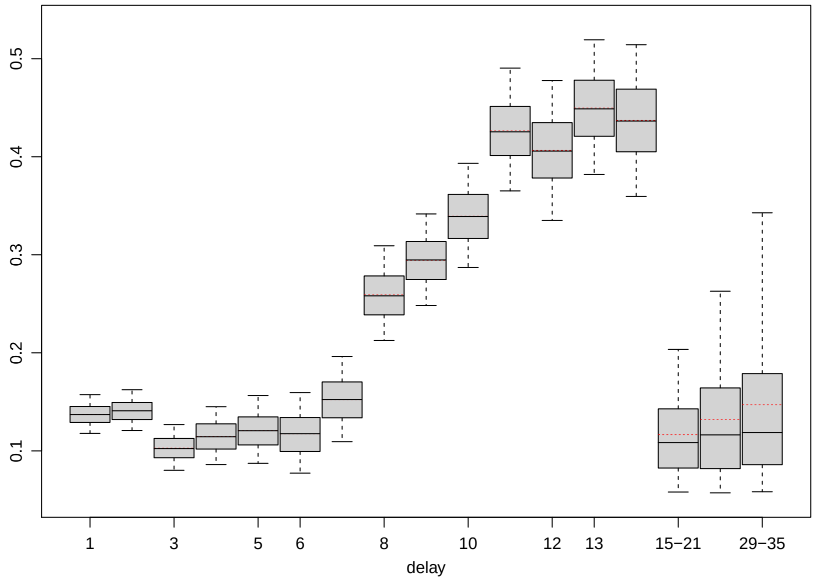

Figure S8: Posterior distributions of $(\phi_d + 1)^{-1/2}$. Boxes show posterior median and quartiles, red bars show posterior means, and whiskers show 95% credible intervals.

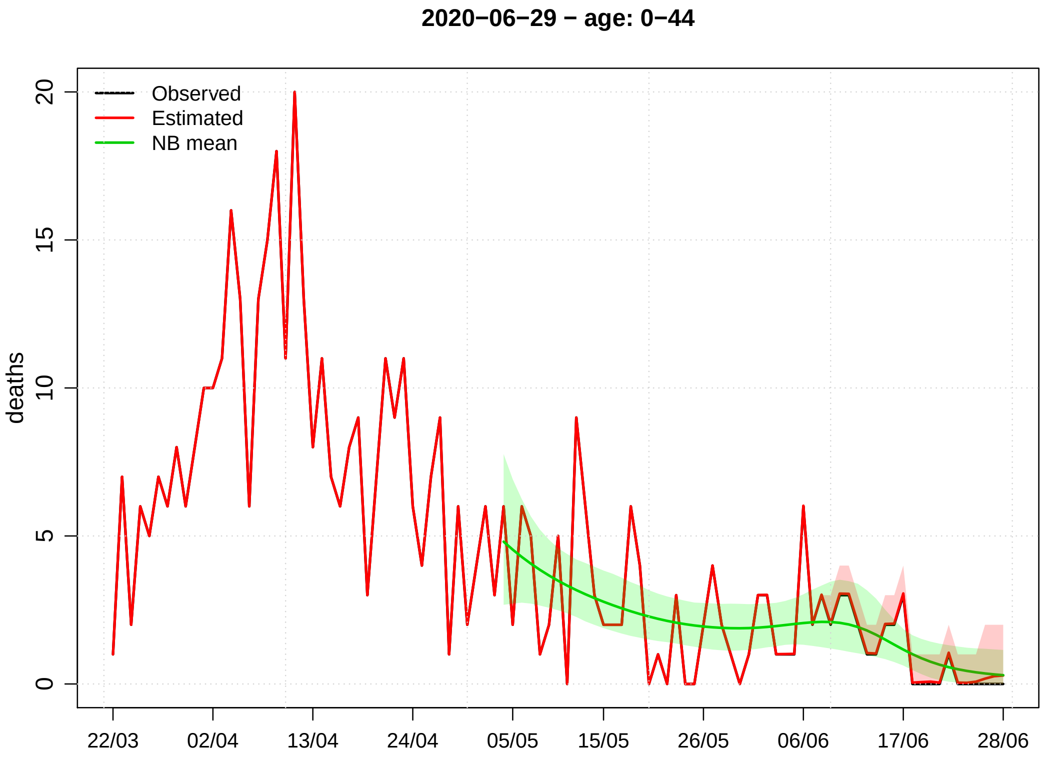

Figure S9: Estimates of the numbers of deaths occurring on each day (solid red line) in the 0–44 stratum, with the numbers of deaths on each day reported by 29th June (black line). Posterior 95% CIs are shown by shaded red region. The green line shows the posterior mean of λ_{st}, the expected number of deaths. Posterior 95% CIs for this expected number are shown by shaded green region.

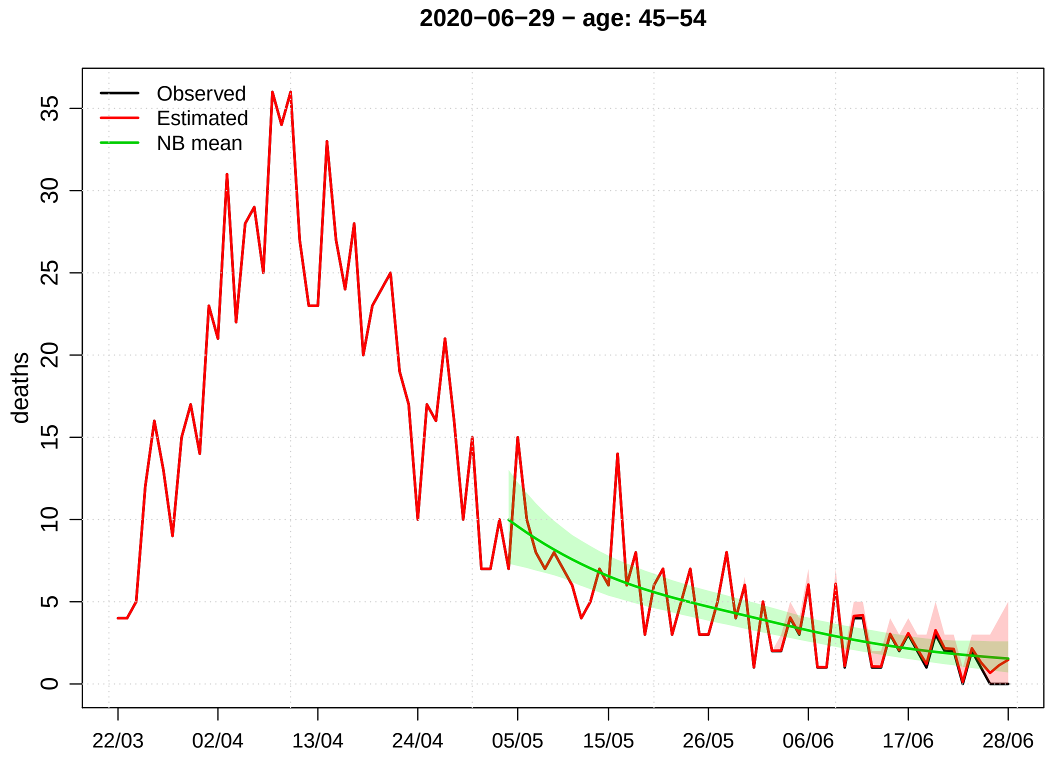

Figure S10: Estimates of the numbers of deaths occurring on each day (solid red line) in the 45–54 stratum, with the numbers of deaths on each day reported by 29th June (black line). Posterior 95% CIs are shown by shaded red region. The green line shows the posterior mean of λ_{st}, the expected number of deaths. Posterior 95% CIs for this expected number are shown by shaded green region.

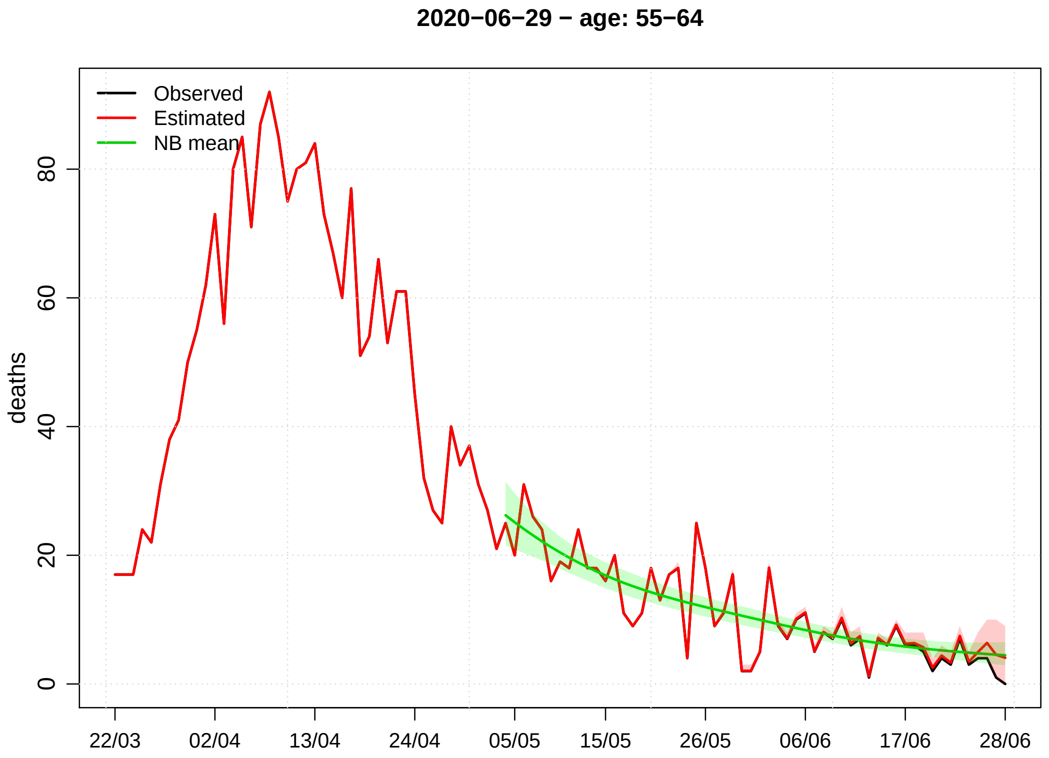

Figure S11: Estimates of the numbers of deaths occurring on each day (solid red line) in the 55–64 stratum, with the numbers of deaths on each day reported by 29th June (black line). Posterior 95% CIs are shown by shaded red region. The green line shows the posterior mean of λ_{st}, the expected number of deaths. Posterior 95% CIs for this expected number are shown by shaded green region.

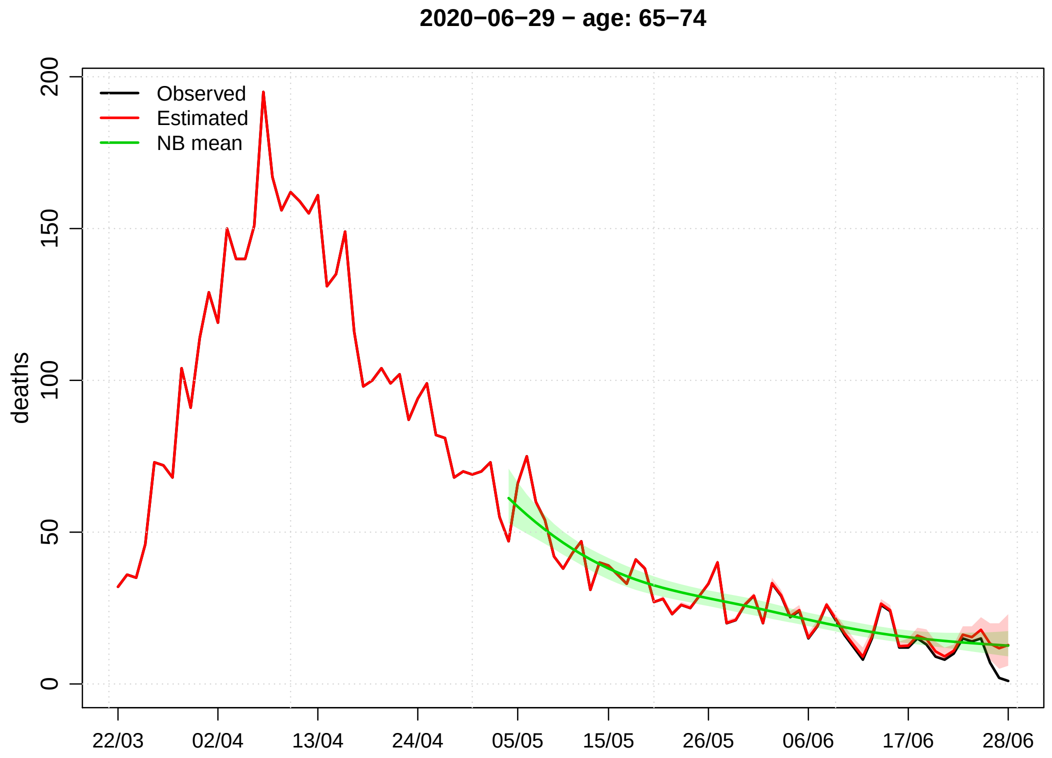

Figure S12: Estimates of the numbers of deaths occurring on each day (solid red line) in the 65–74 stratum, with the numbers of deaths on each day reported by 29th June (black line). Posterior 95% CIs are shown by shaded red region. The green line shows the posterior mean of λ_{st}, the expected number of deaths. Posterior 95% CIs for this expected number are shown by shaded green region.

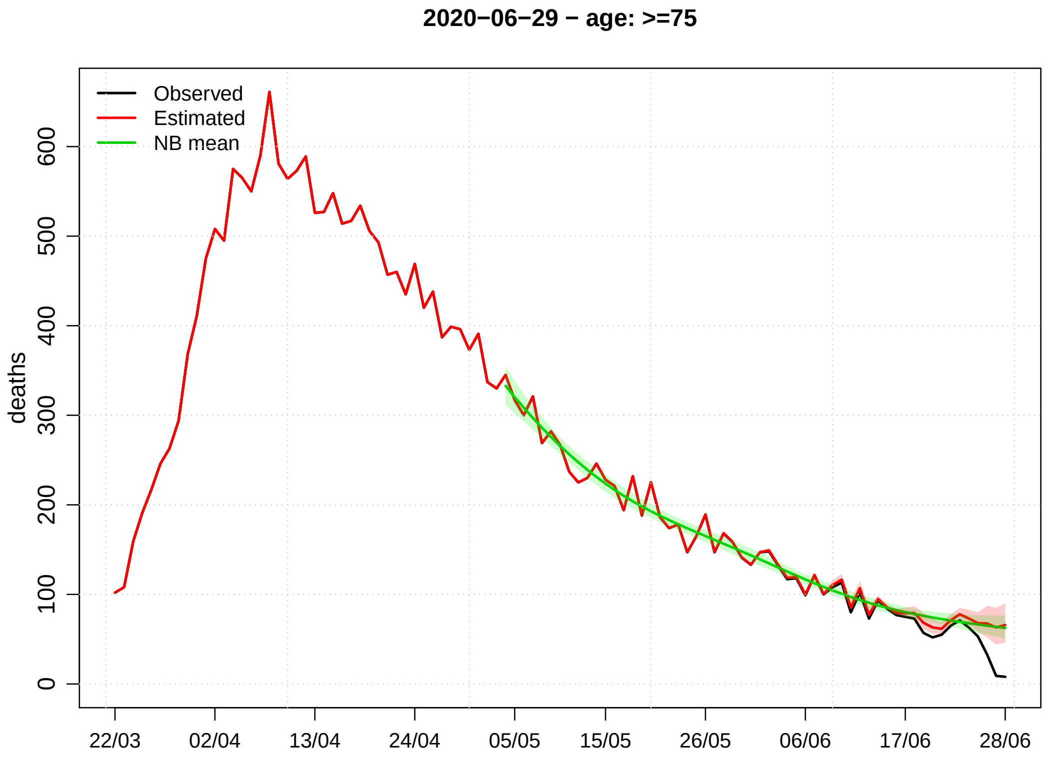

Figure S13: Estimates of the numbers of deaths occurring on each day (solid red line) in the ≥ 75 stratum, with the numbers of deaths on each day reported by 29th June (black line). Posterior 95% CIs are shown by shaded red region. The green line shows the posterior mean of λ_{st}, the expected number of deaths. Posterior 95% CIs for this expected number are shown by shaded green region.

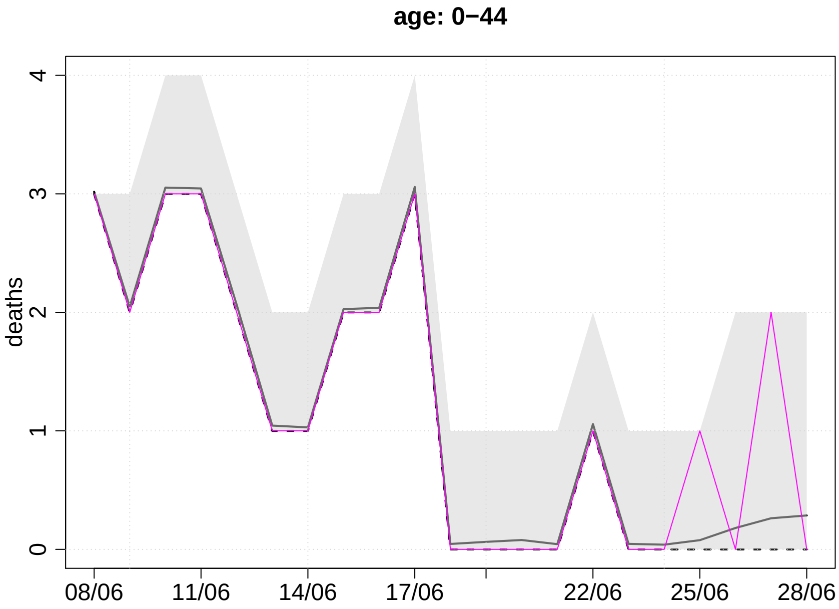

Figure S14: Estimates of the numbers of deaths occurring on each of the last 21 days (solid line) in the 0–44 stratum (black line), with numbers of deaths on each day reported by 29th June (broken line) and the true numbers (purple line). Posterior 95% CIs are shown by shaded region.

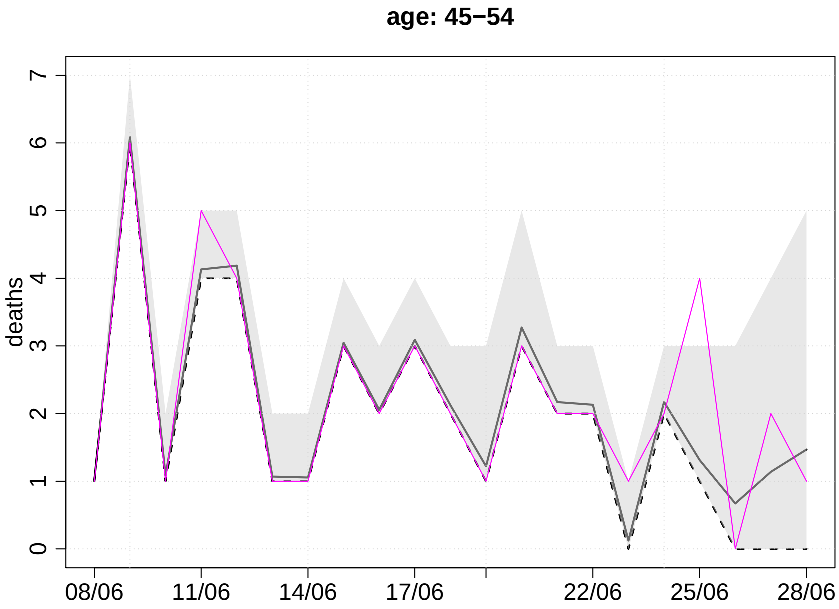

Figure S15: Estimates of the numbers of deaths occurring on each of the last 21 days (solid line) in the 45–54 stratum (black line), with numbers of deaths on each day reported by 29th June (broken line) and the true numbers (purple line). Posterior 95% CIs are shown by shaded region.

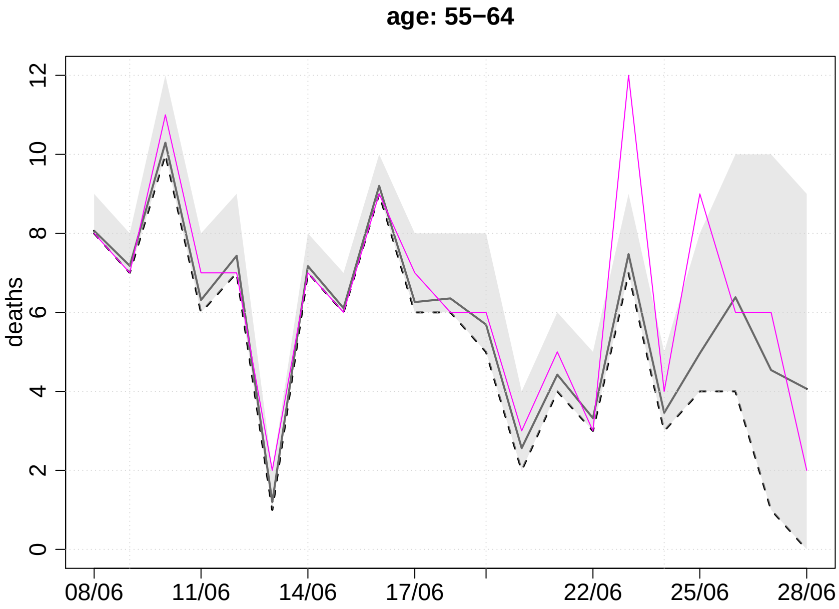

Figure S16: Estimates of the numbers of deaths occurring on each of the last 21 days (solid line) in the 55–64 stratum (black line), with numbers of deaths on each day reported by 29th June (broken line) and the true numbers (purple line). Posterior 95% CIs are shown by shaded region.

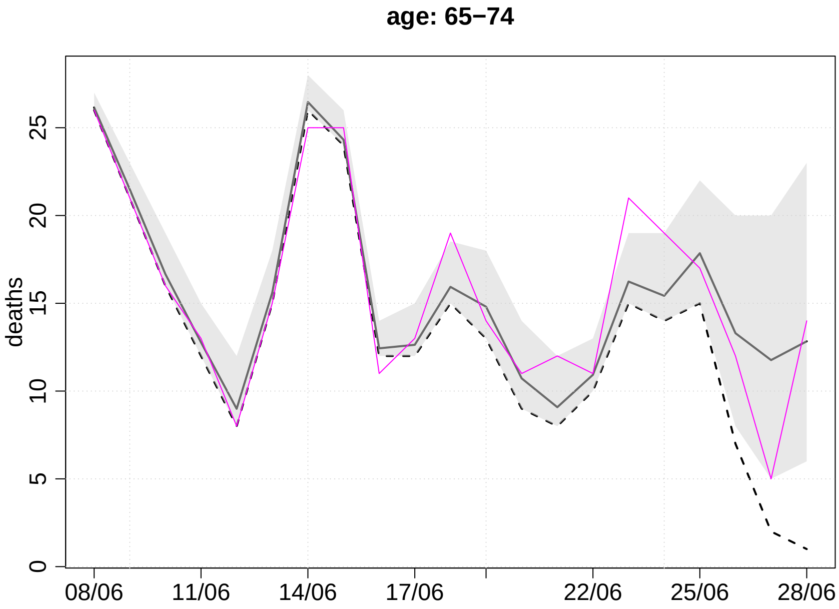

Figure S17: Estimates of the numbers of deaths occurring on each of the last 21 days (solid line) in the 65–74 stratum (black line), with numbers of deaths on each day reported by 29th June (broken line) and the true numbers (purple line). Posterior 95% CIs are shown by shaded region.

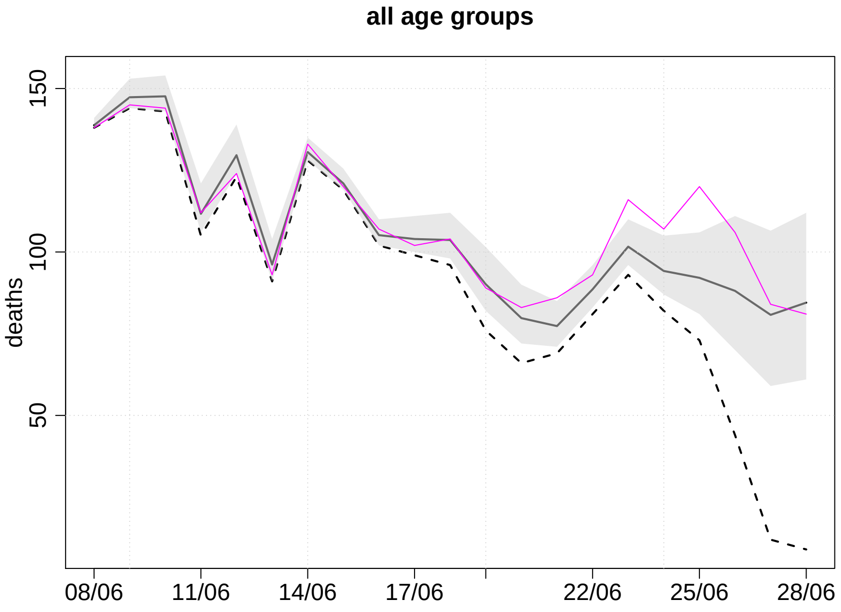

Figure S18: Estimates of the numbers of deaths occurring on each of the last 21 days (solid line) in all age strata combined (black line), with numbers of deaths on each day reported by 29th June (broken line) and the true numbers (purple line). Posterior 95% CIs are shown by shaded region.

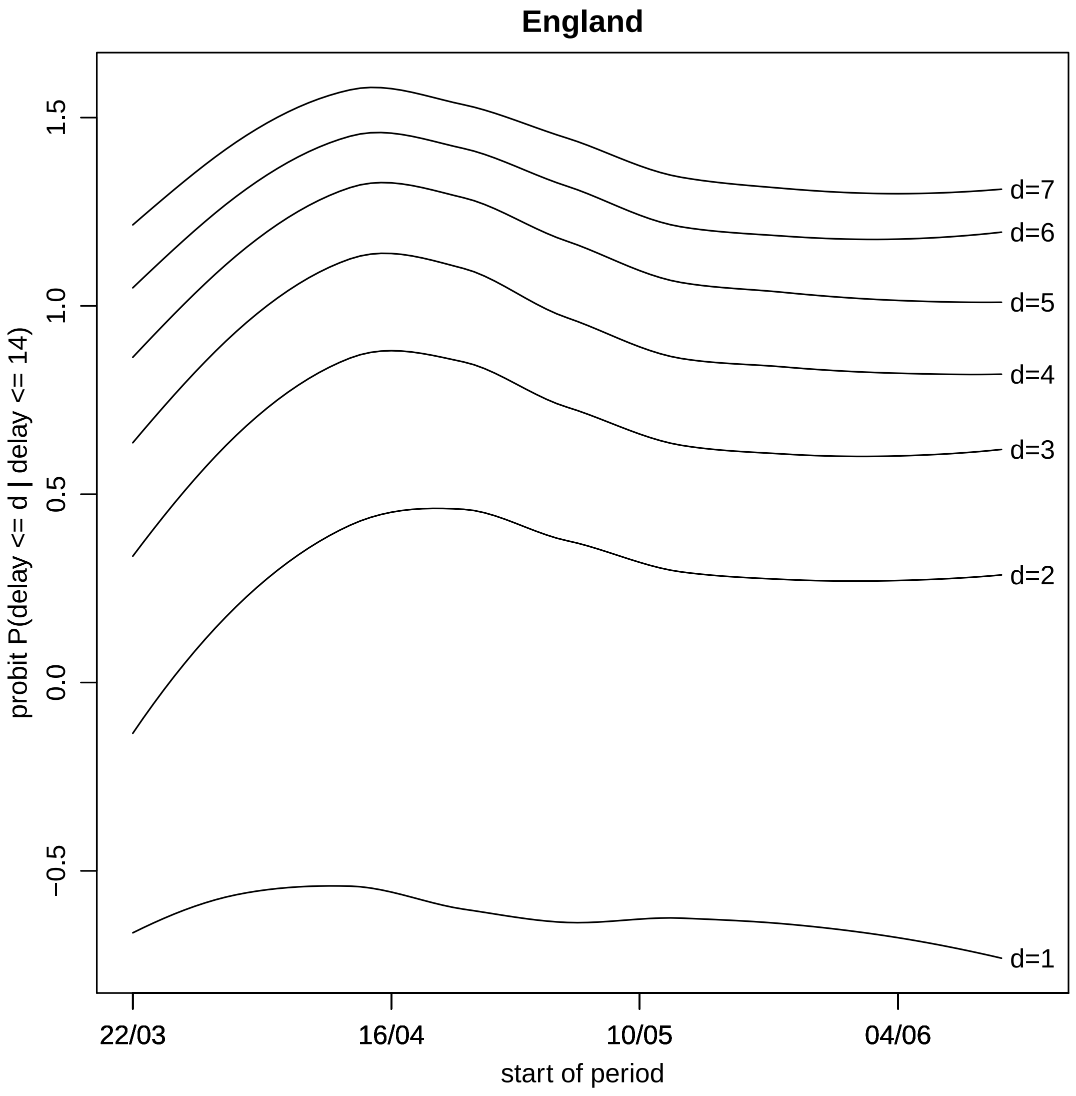

Figure S19: Estimated probit-transformed probability that delay is at most d days as a function of date of death. Only $d = 1, \ldots, 7$ are shown, probabilities are conditional on delay being at most 14 days, and estimates are given for each day up to 14 days before 29th June. (Estimate is calculated by applying loess estimator to observed proportions.)

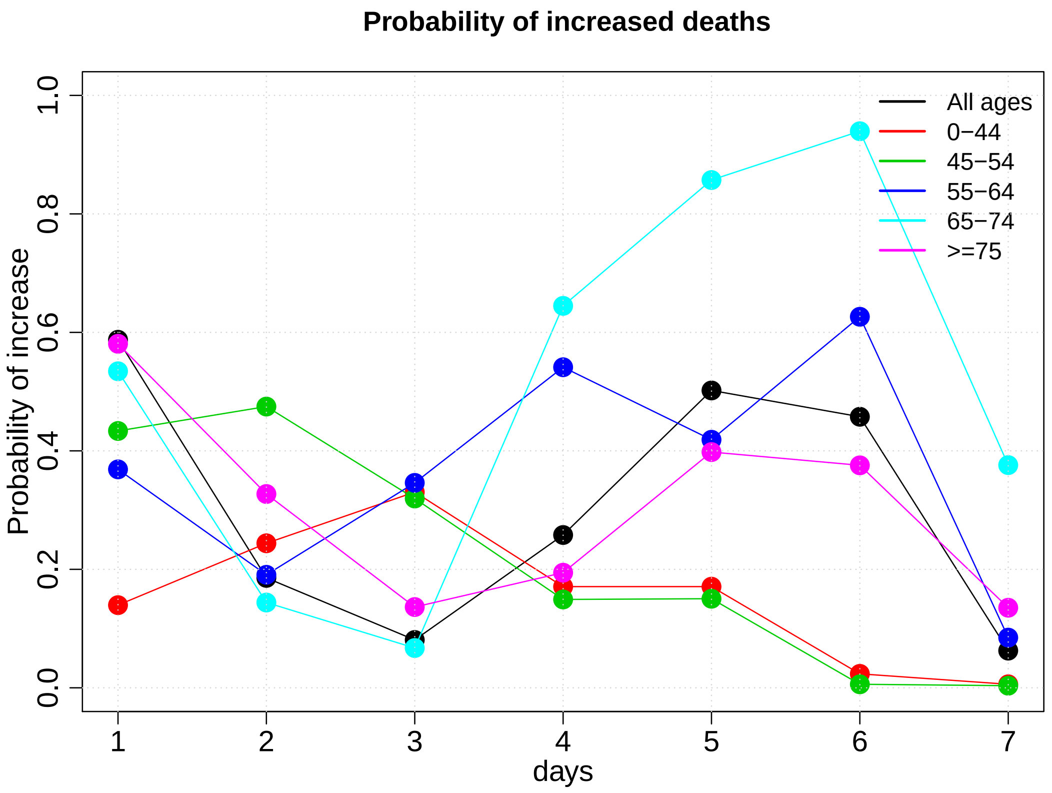

Figure S20: Posterior probabilities that the number of deaths in the most recent x days was greater than the number in the preceding x days ($x = 1, \dots 7$)

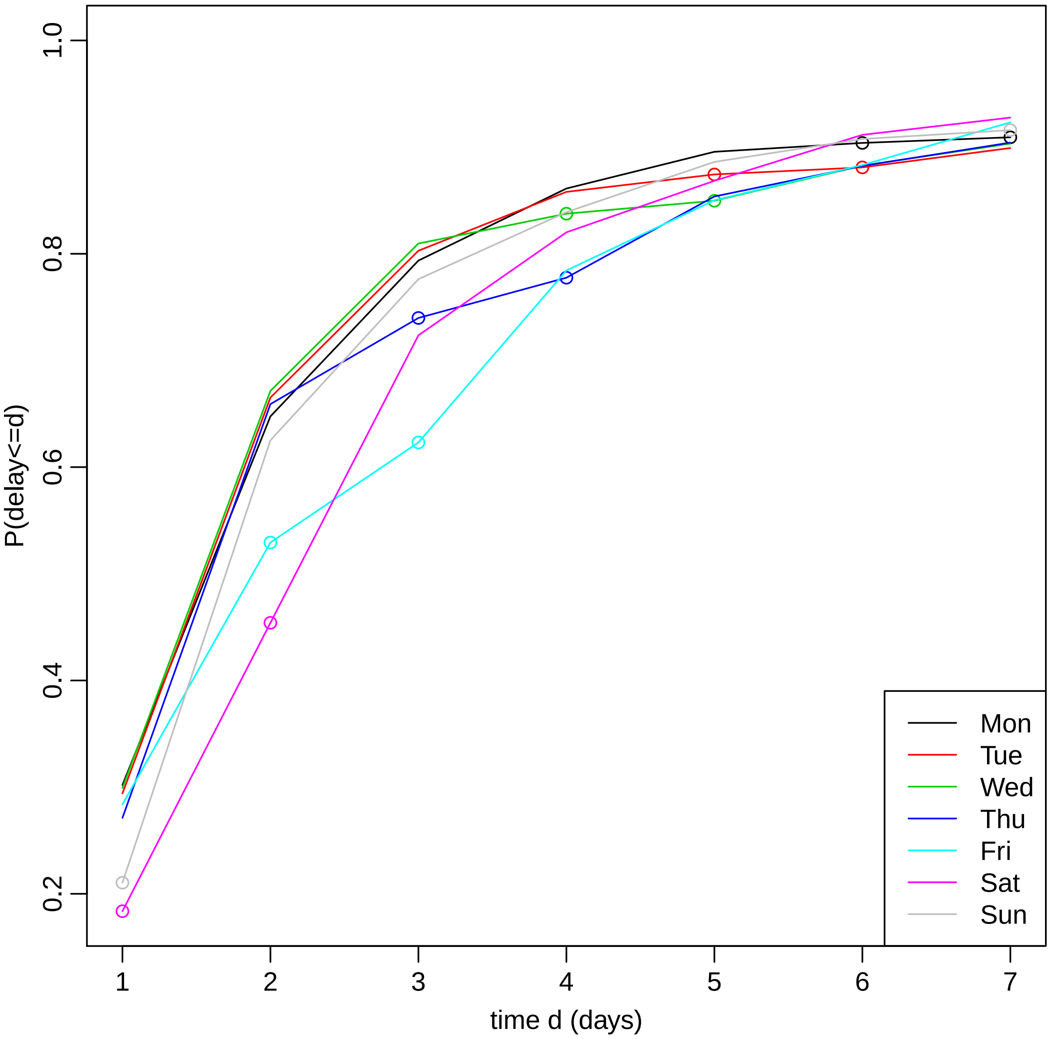

Figure S21: Estimated cumulative distribution function of delay given day of the week of death (only days 1–7 shown). The dots indicate Sundays and Mondays. (Estimate calculated using deaths that occurred at least 42 days before 29th June.)

S3 Weekday effect example in Section 4

For simplicity, we ignore g_{st}^{C} and g_{st}^{W} and omit the subscript s for stratum. Suppose deaths are never reported on Sundays but are reported on all other days of the week, and suppose day t is a Sunday. For someone who died on day t, the probability of being reported on day t equals zero, and hence q_{t0} equals zero, but their (expected) probability q_{t1} of being reported by the end of the following day would be non-zero, say $q_{t1} = c_t$. So, from S&E's equation

$$\text{probit}(q_{std}) = \text{probit}(q_{sd}^*) + g_{st}^{\mathrm{C}} + h_{st}, \tag{3}$$

we have that $\text{probit}(0) = \text{probit}(q_0^*) + h_t$ and $\text{probit}(c_t) = \text{probit}(q_1^*) + h_t$. Since $c_t > 0$, it follows that $q_1^* > q_0^*$.

On the other hand, for someone who died on day $t - 1$, which was Saturday, the (expected) probability $q_{t-1,0}$ of being reported on day $t-1$ would be non-zero, say $q_{t-1,0} = c_{t-1}$, and their probability of being reported by the end of the following day would be no greater, i.e. $q_{t-1,1} = q_{t-1,0} = c_{t-1}$. So, from equation (3) we have that $\text{probit}(c_{t-1}) = \text{probit}(q_0^*)+h_{t-1}$ and $\text{probit}(c_{t-1}) = \text{probit}(q_1^*)+h_{t-1}$. It follows from this that $q_1^* = q_0^*$, which contradicts our earlier deduction that $q_1^* > q_0^*$.

S4 Analysis of London data available on 29th June

In this section, we provide results from London using data available at 29th June.

Figures S22–S24 show the posterior distribution of the weekday effects on the delay distribution in the age strata.

Figure S25–S27 shows the posterior mean and pointwise 95% posterior credible interval (CI) of the change in the weekend effect on the delay distribution in the age strata.

Figure S28–S30 shows the posterior mean and pointwise 95% posterior CI of the calendar-time effect on the delay distribution in the age strata.

Figure S31 shows the posterior distributions of the reporting-day random effects.

Figure S32 shows the posterior distribution of $(\phi_d + 1)^{-1/2}$ for each delay d.

Figures S33– S37 show the nowcasts for the five age strata.

Figures S38– S42 are enlargements of these nowcasts, showing only the most recent 21 days.

Figure S43 shows, for each of the strata, the posterior probability that the number of deaths in the most recent x days was greater than the number in the preceding x days ($x = 1, \ldots 7$).

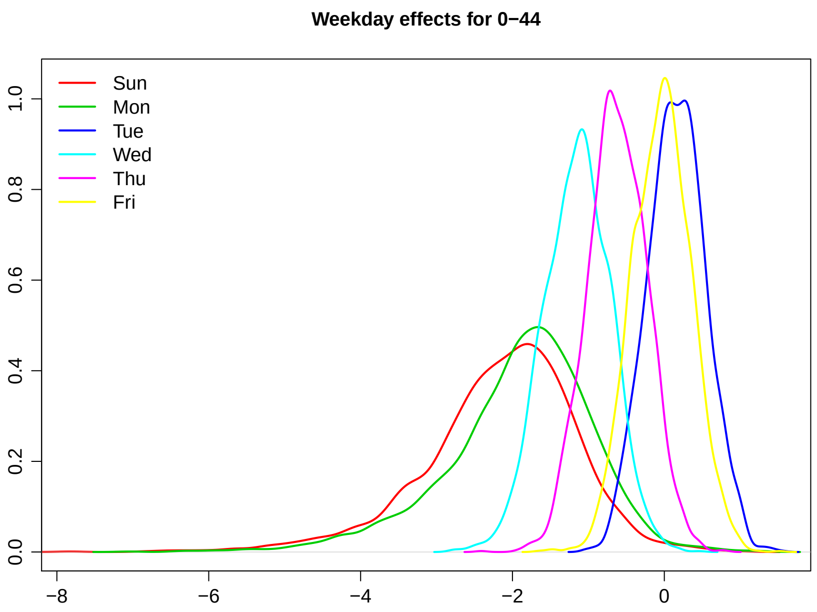

Figure S22: Posterior distributions of weekday effects (η_j) in the 0–44 age stratum in London

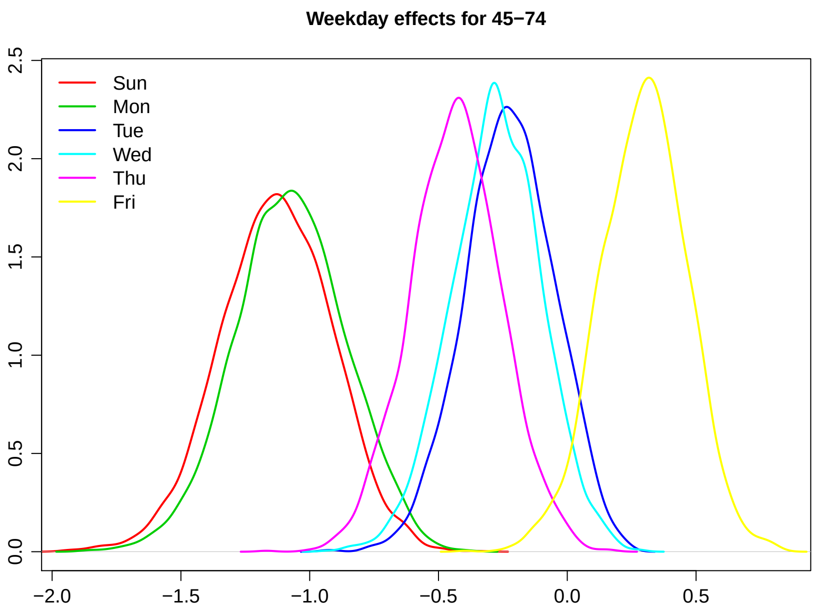

Figure S23: Posterior distributions of weekday effects (η_j) in the three 45–74 age strata in London

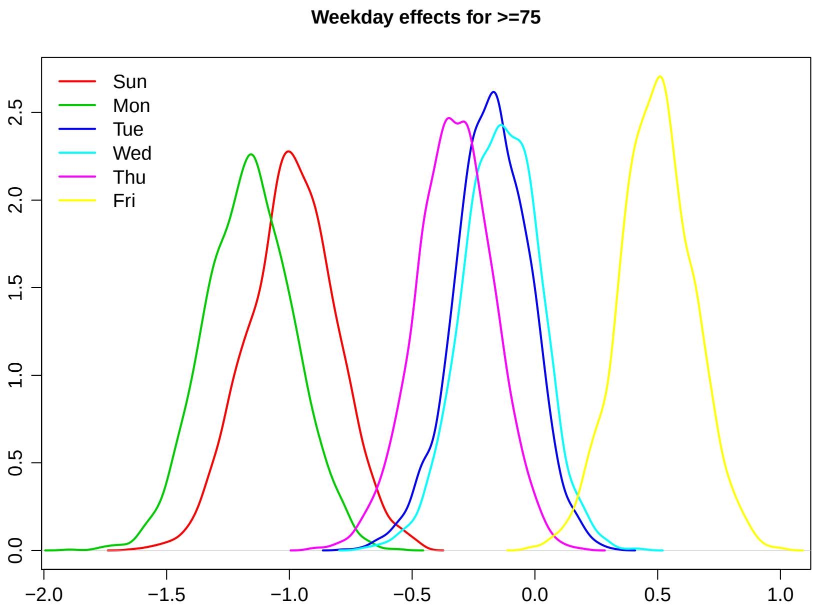

Figure S24: Posterior distributions of weekday effects (η_j) in the ≥ 75 age stratum in London

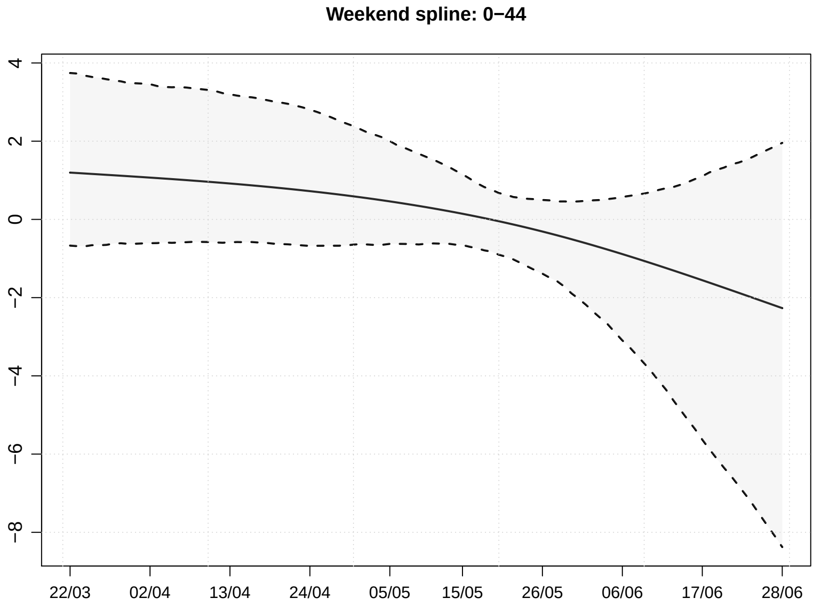

Figure S25: Posterior mean and 95% CI of the change in the weekend effect (g^{W}_{st}) in the 0–44 age stratum in London

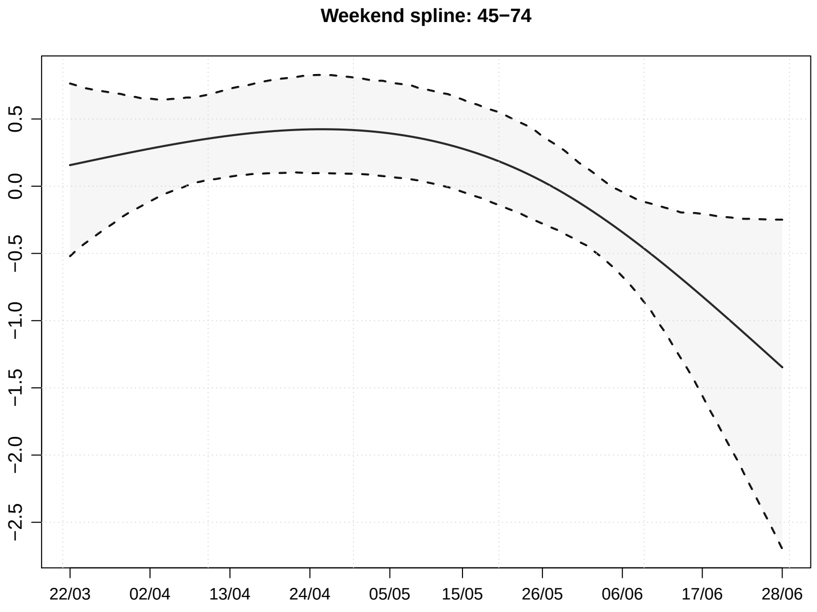

Figure S26: Posterior mean and 95% CI of the change in the weekend effect (g_{st}^{W}) in the three 45–74 age strata in London

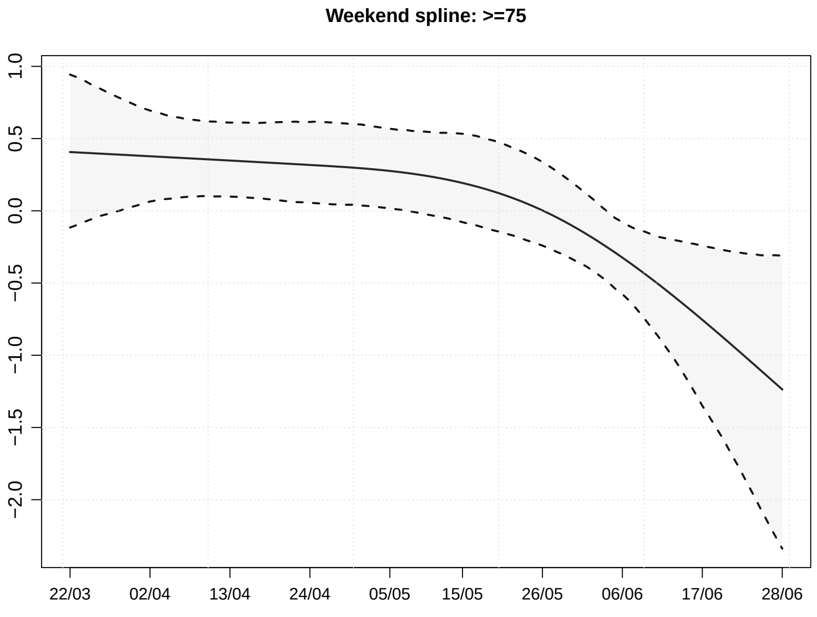

Figure S27: Posterior mean and 95% CI of the change in the weekend effect (g_{st}^{W}) in the ≥ 75 age stratum in London

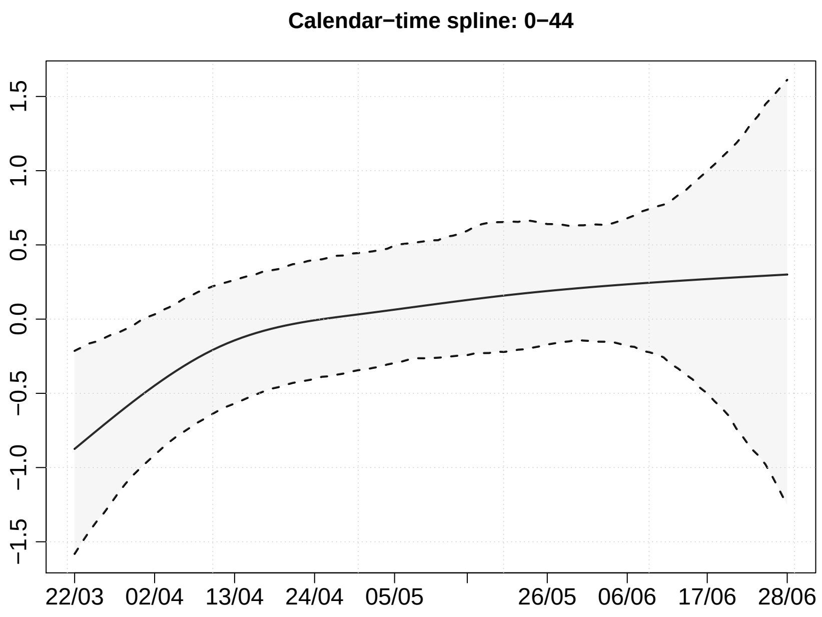

Figure S28: Posterior mean and 95% CI of the calendar-time effect (g_{st}^{C}) in the 0–44 age stratum in London

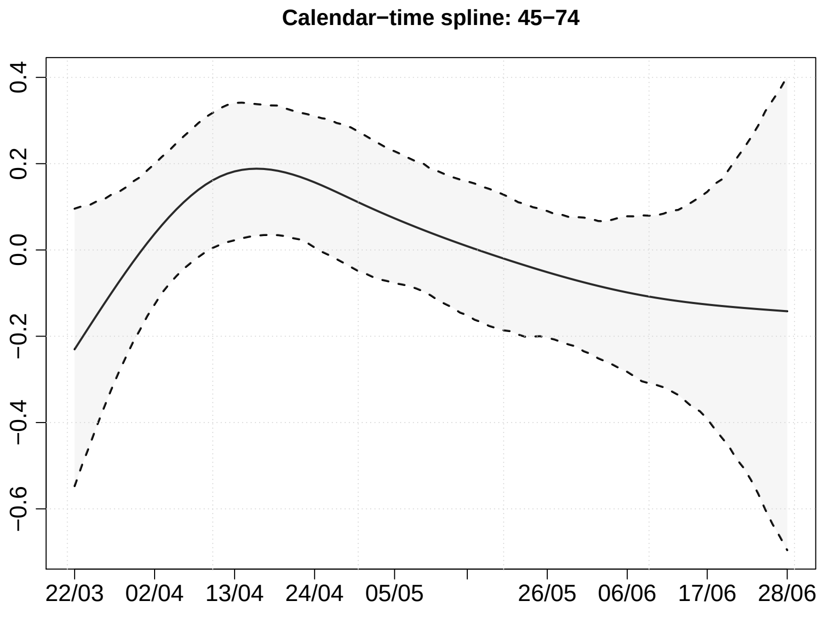

Figure S29: Posterior mean and 95% CI of the calendar-time effect (g_{st}^{C}) in the three 45–74 age strata in London

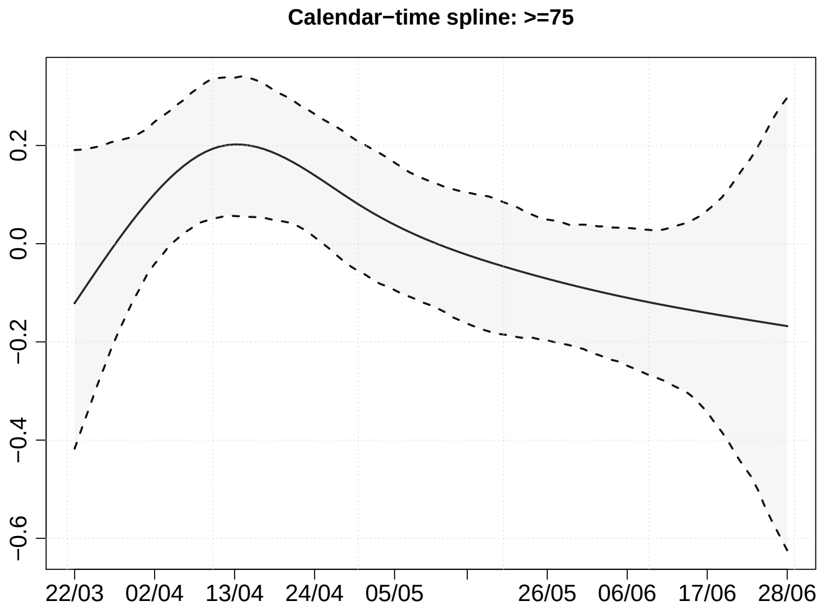

Figure S30: Posterior mean and 95% CI of the calendar-time effect (g_{st}^{C}) in the ≥ 75 age stratum in London

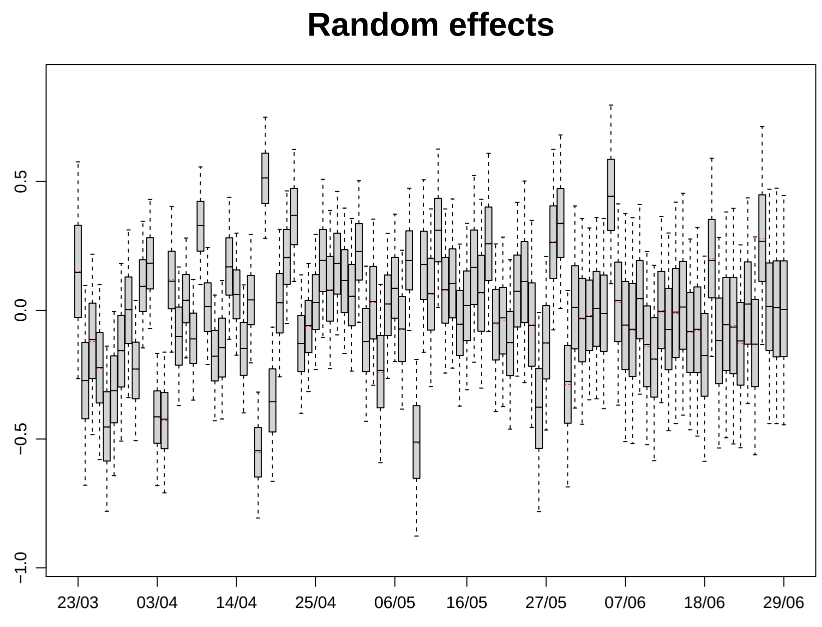

Figure S31: Posterior distributions of reporting-day random effects (δ_{t+d}) for London. Boxes show posterior median and quartiles, red bars show posterior means, and whiskers show 95% credible intervals.

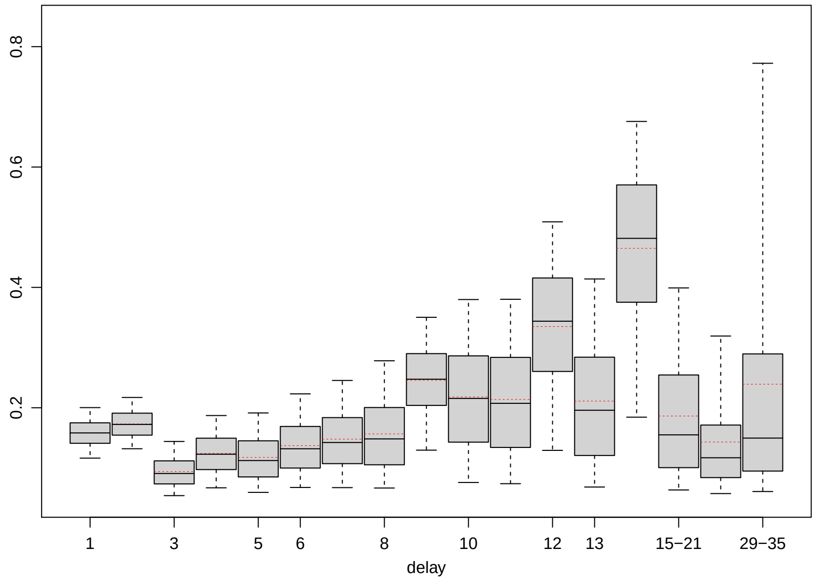

Figure S32: Posterior distributions of $(\phi_d+1)^{-1/2}$ for London. Boxes show posterior median and quartiles, red bars show posterior means, and whiskers show 95% credible intervals.

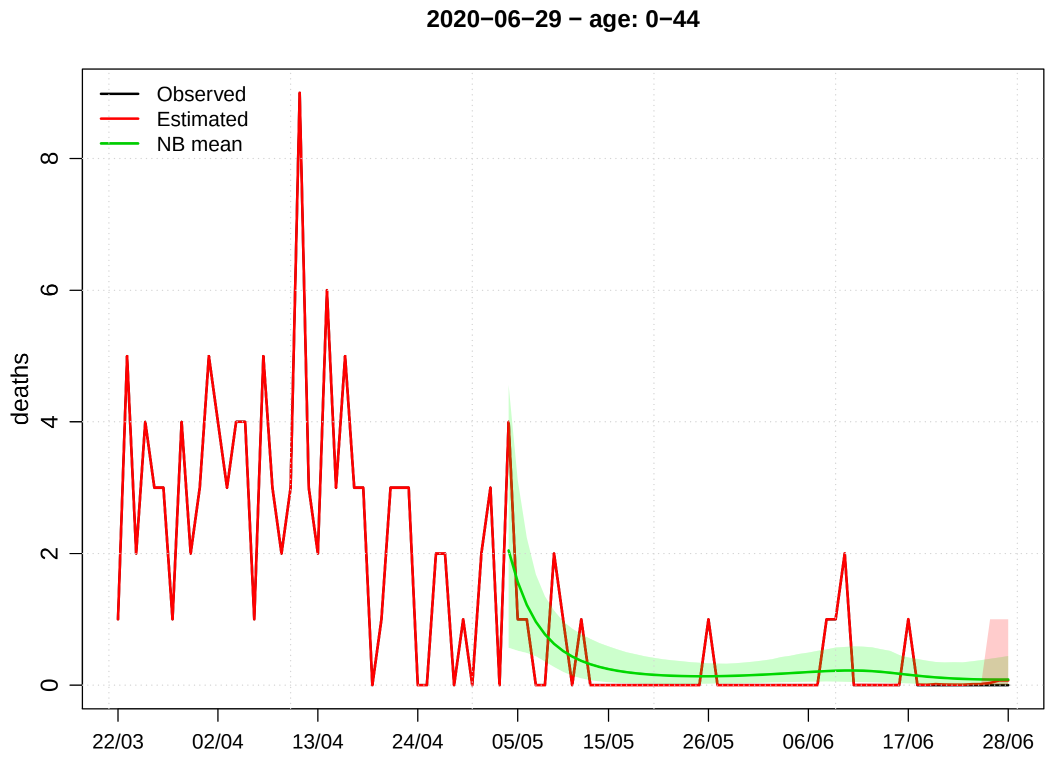

Figure S33: Estimates of the numbers of deaths occurring on each day (solid red line) in the 0–44 stratum in London, with the numbers of deaths on each day reported by 29th June (black line). Posterior 95% CIs are shown by shaded red region. The green line shows the posterior mean of λ_{st}, the expected number of deaths. Posterior 95% CIs for this expected number are shown by shaded green region.

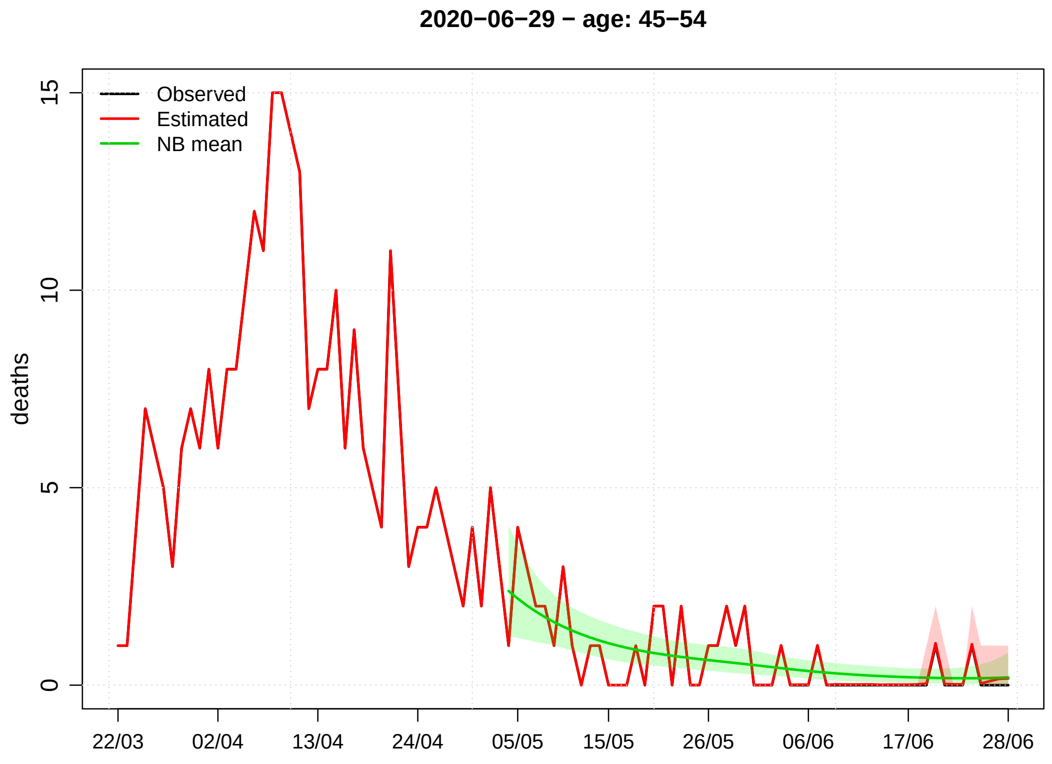

Figure S34: Estimates of the numbers of deaths occurring on each day (solid red line) in the 45–54 stratum in London, with the numbers of deaths on each day reported by 29th June (black line). Posterior 95% CIs are shown by shaded red region. The green line shows the posterior mean of λ_{st}, the expected number of deaths. Posterior 95% CIs for this expected number are shown by shaded green region.

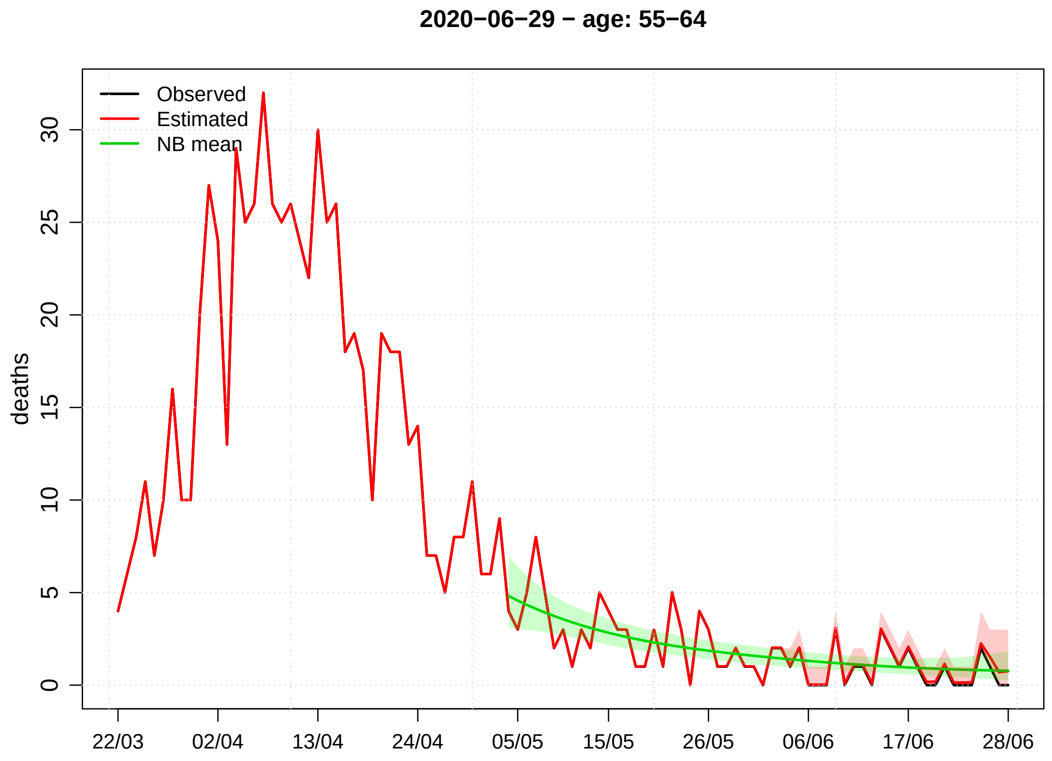

Figure S35: Estimates of the numbers of deaths occurring on each day (solid red line) in the 55–64 stratum in London, with the numbers of deaths on each day reported by 29th June (black line). Posterior 95% CIs are shown by shaded red region. The green line shows the posterior mean of λ_{st}, the expected number of deaths. Posterior 95% CIs for this expected number are shown by shaded green region.

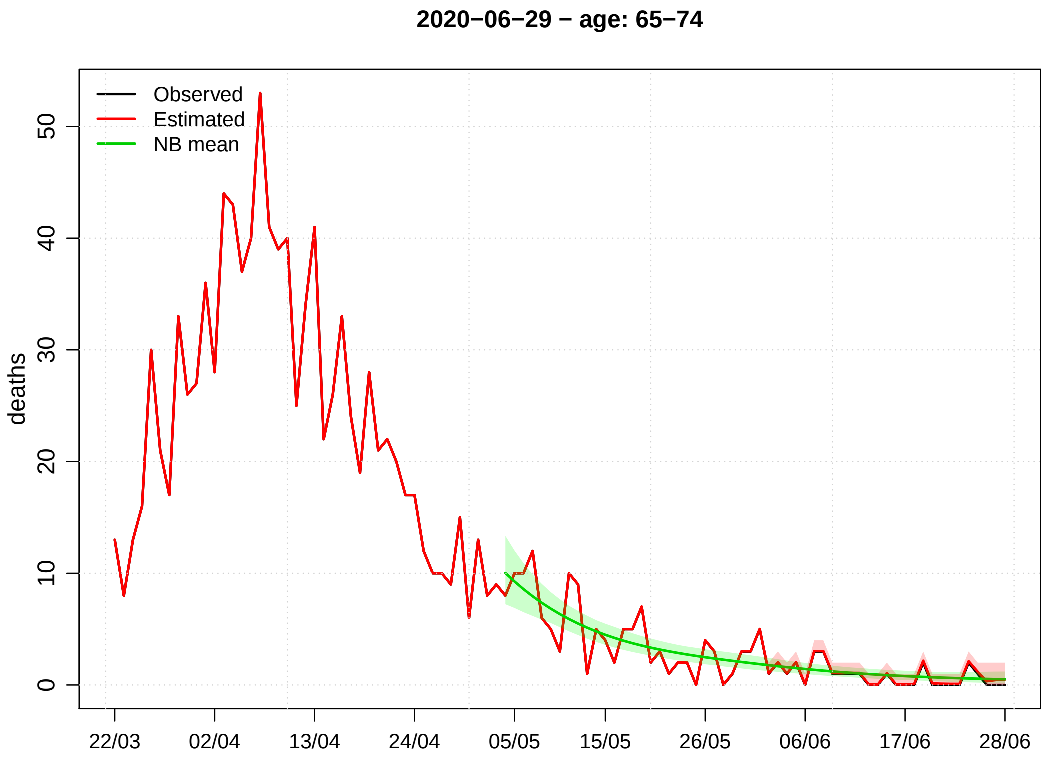

Figure S36: Estimates of the numbers of deaths occurring on each day (solid red line) in the 65–74 stratum in London, with the numbers of deaths on each day reported by 29th June (black line). Posterior 95% CIs are shown by shaded red region. The green line shows the posterior mean of λ_{st}, the expected number of deaths. Posterior 95% CIs for this expected number are shown by shaded green region.

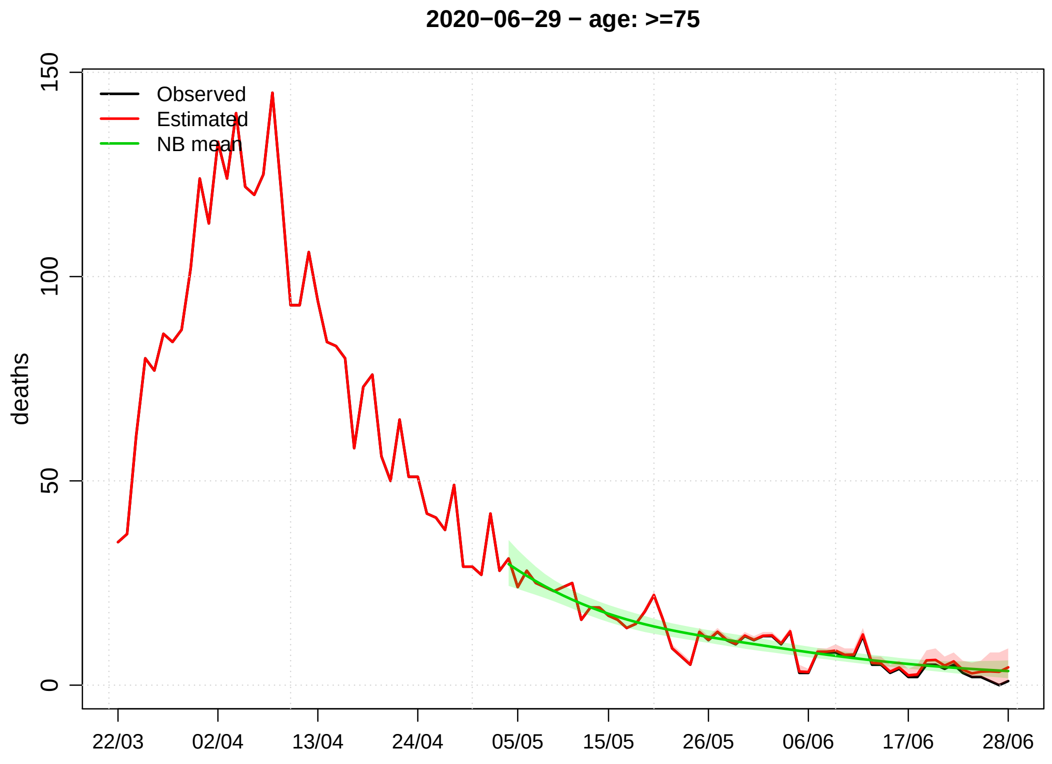

Figure S37: Estimates of the numbers of deaths occurring on each day (solid red line) in the $\geq$ 75 stratum in London, with the numbers of deaths on each day reported by 29th June (black line). Posterior 95% CIs are shown by shaded red region. The green line shows the posterior mean of λ_{st}, the expected number of deaths. Posterior 95% CIs for this expected number are shown by shaded green region.

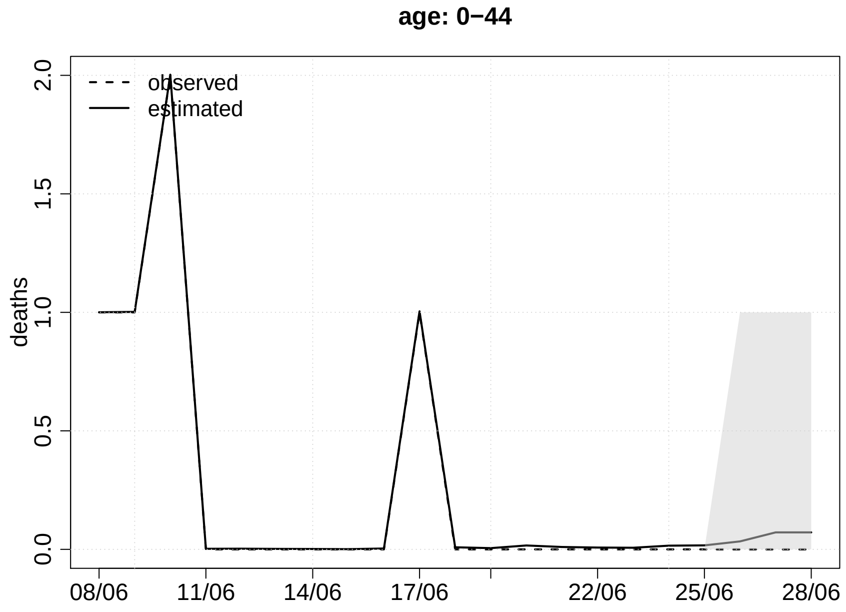

Figure S38: Estimates of the numbers of deaths occurring on each of the last 21 days (solid line) in the 0–44 stratum in London, with numbers of deaths on each day reported by 29th June (broken line). Posterior 95% CIs are shown by shaded region.

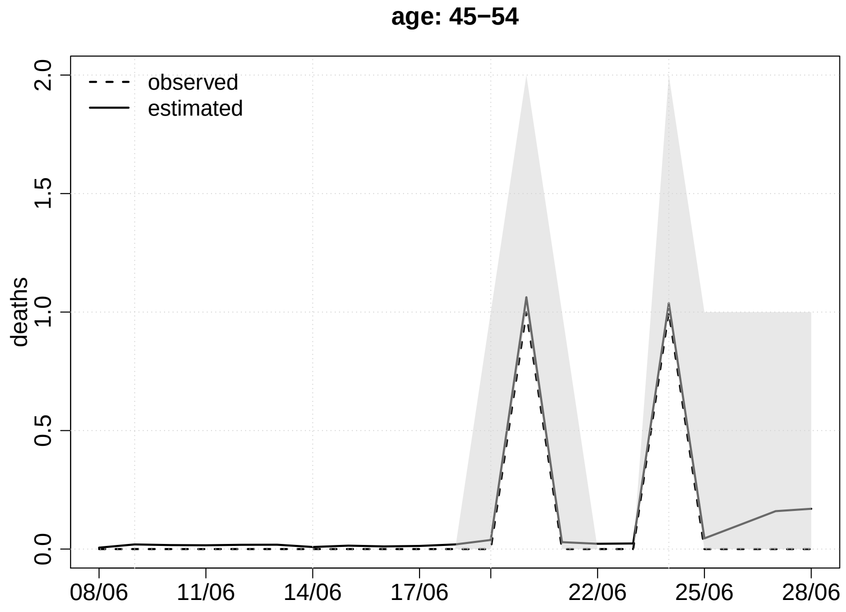

Figure S39: Estimates of the numbers of deaths occurring on each of the last 21 days (solid line) in the 45–54 stratum in London, with numbers of deaths on each day reported by 29th June (broken line). Posterior 95% CIs are shown by shaded region.

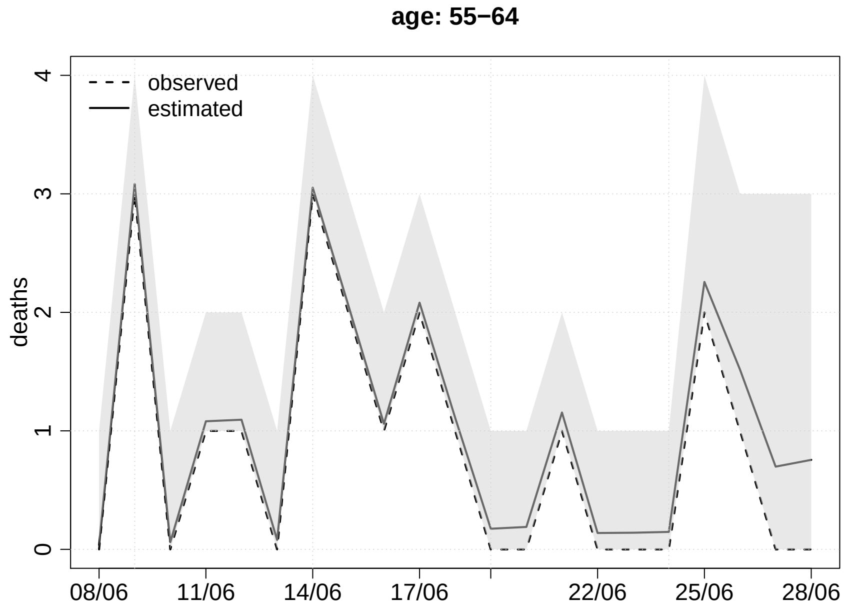

Figure S40: Estimates of the numbers of deaths occurring on each of the last 21 days (solid line) in the 55–64 stratum in London, with numbers of deaths on each day reported by 29th June (broken line). Posterior 95% CIs are shown by shaded region.

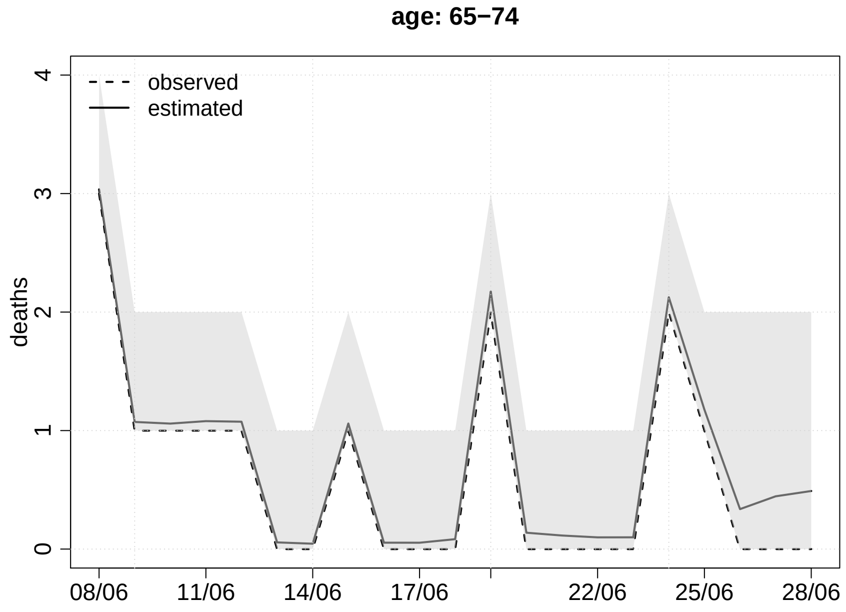

Figure S41: Estimates of the numbers of deaths occurring on each of the last 21 days (solid line) in the 65–74 stratum in London, with numbers of deaths on each day reported by 29th June (broken line). Posterior 95% CIs are shown by shaded region.

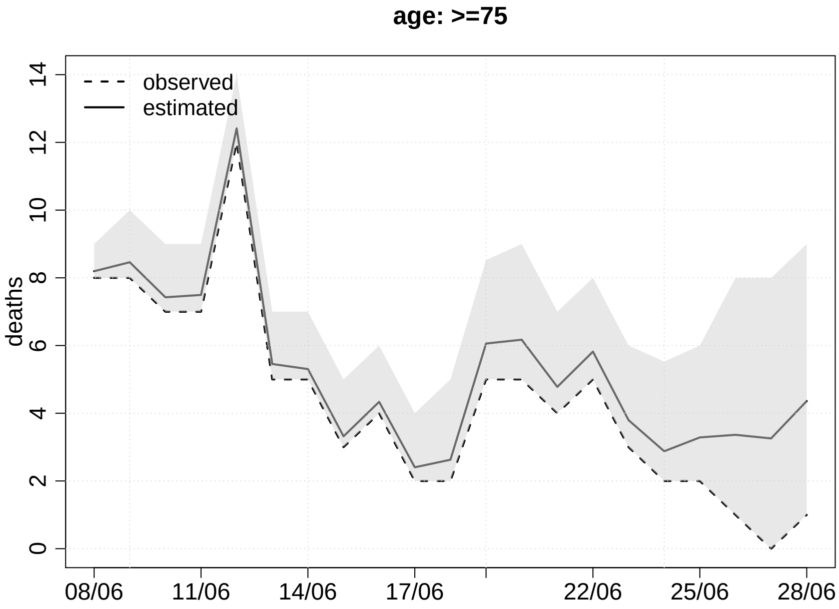

Figure S42: Estimates of the numbers of deaths occurring on each of the last 21 days (solid line) in the ≥ 75 stratum in London, with numbers of deaths on each day reported by 29th June (broken line). Posterior 95% CIs are shown by shaded region.

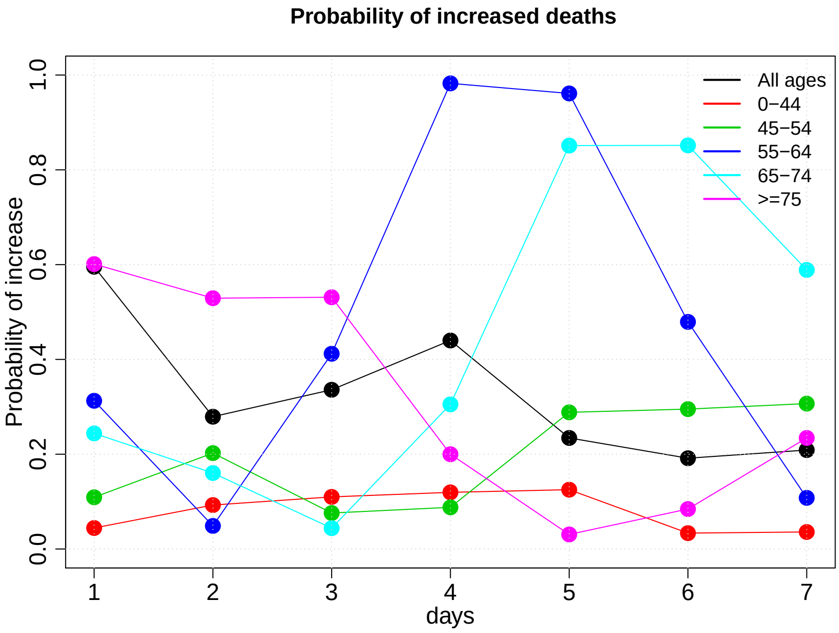

Figure S43: Posterior probabilities that the number of deaths in London in the most recent x days was greater than the number in the preceding x days ($x = 1, \ldots 7$)

S5 Nowcasts for other regions

Figure S44 shows the nowcasts for the five age strata for the Southeast region. Figures S45–S50 are enlargements of these nowcasts, showing only the most recent 21 days.

Figures S51–S57 show the corresponding results for the Southwest region.

Figures S58–S64 show the corresponding results for the East region.

Figures S65–S71 show the corresponding results for the Midlands region.

Figures S72–S78 show the corresponding results for the Northeast region.

Figures S79–S85 show the corresponding results for the Northwest region.

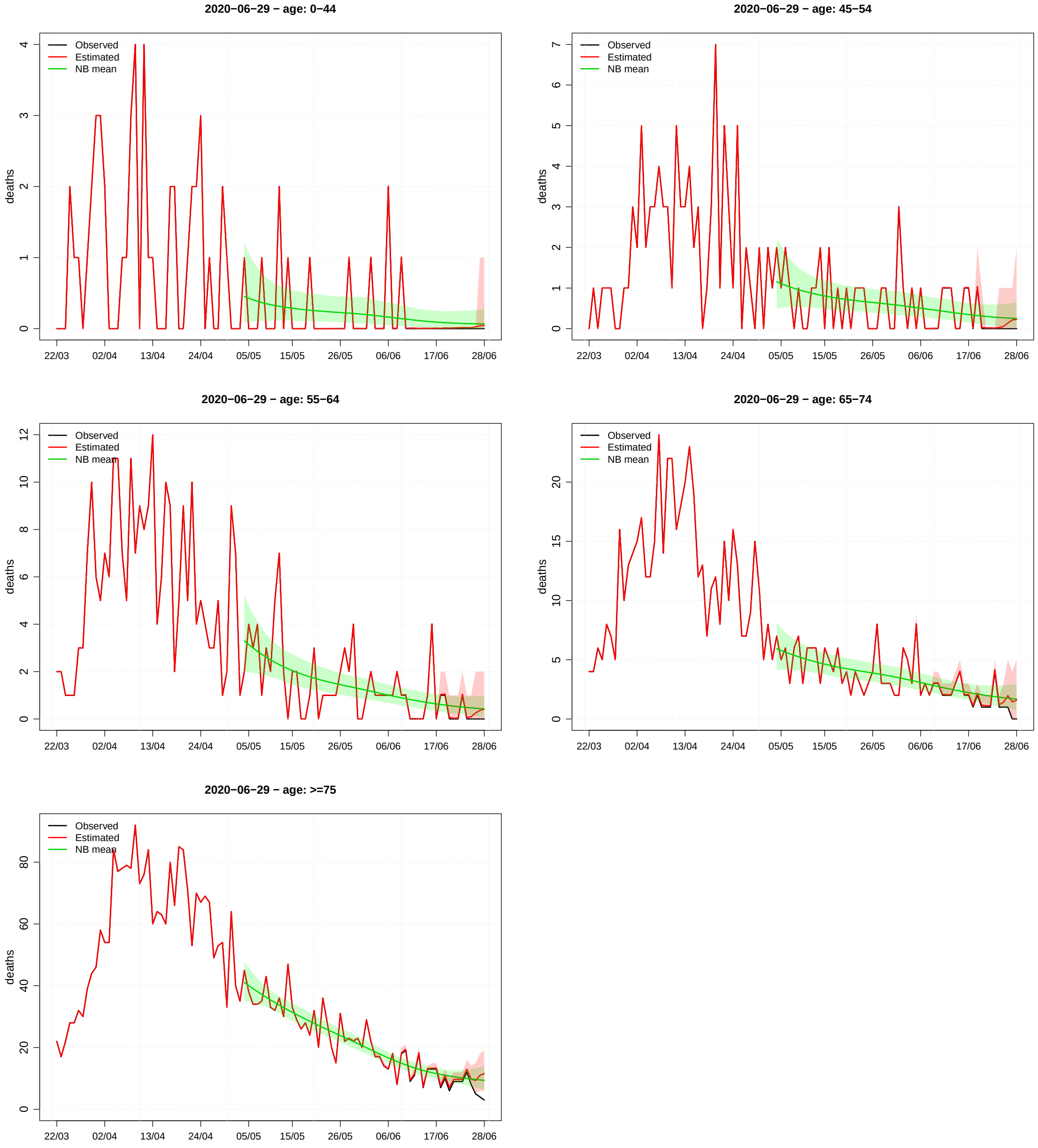

Figure S44: Estimates of the numbers of deaths occurring on each day in Southeast (red line), with numbers of deaths on each day reported by 29th June (black line). Posterior 95% CIs are shown by shaded red region. The green line shows the posterior mean of λ_{st}, the expected number of deaths. Posterior 95% CIs for this expected number are shown by shaded green region.

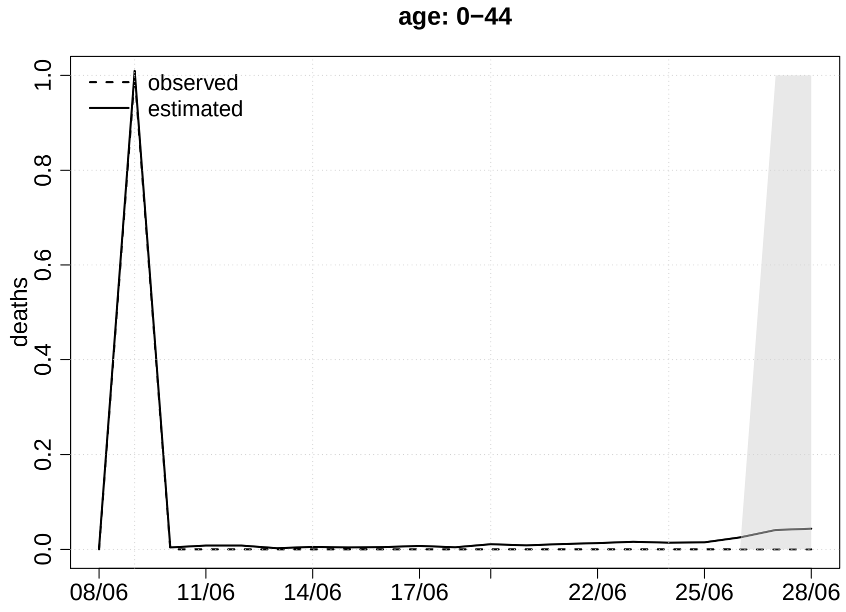

Figure S45: Estimates of the numbers of deaths occurring on each of the last 21 days (solid line) in the 0–44 stratum for Southeast, with numbers of deaths on each day reported by 29th June (broken line). Posterior 95% CIs are shown by shaded region.

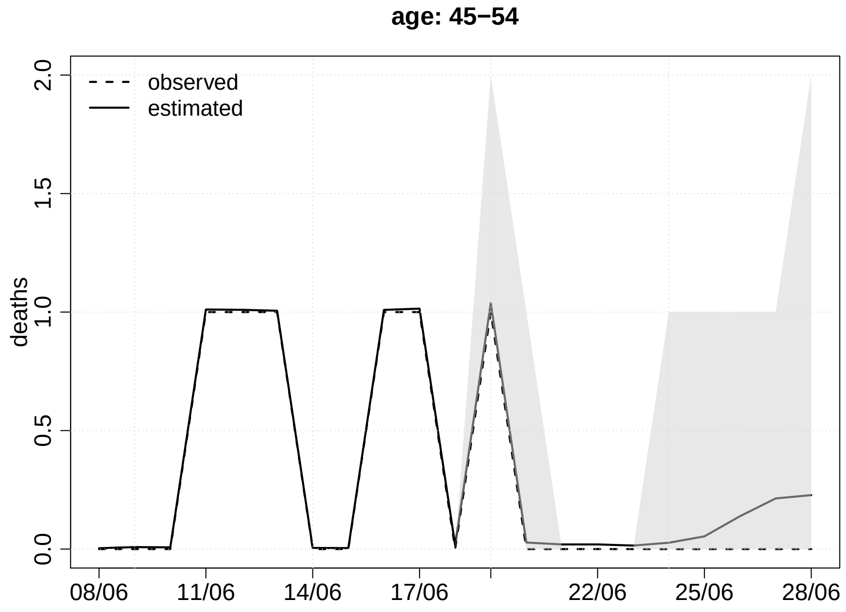

Figure S46: Estimates of the numbers of deaths occurring on each of the last 21 days (solid line) in the 45–54 stratum for Southeast, with numbers of deaths on each day reported by 29th June (broken line). Posterior 95% CIs are shown by shaded region.

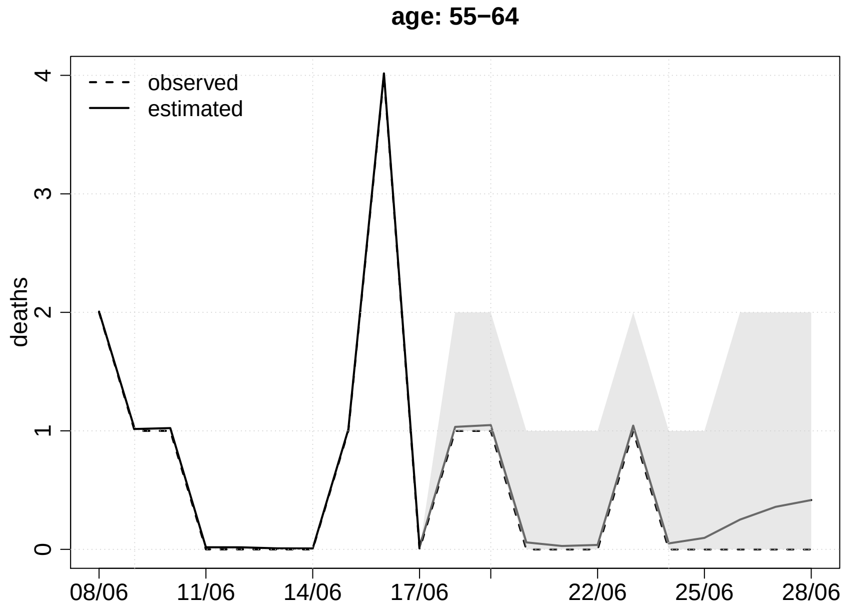

Figure S47: Estimates of the numbers of deaths occurring on each of the last 21 days (solid line) in the 55–64 stratum for Southeast, with numbers of deaths on each day reported by 29th June (broken line). Posterior 95% CIs are shown by shaded region.

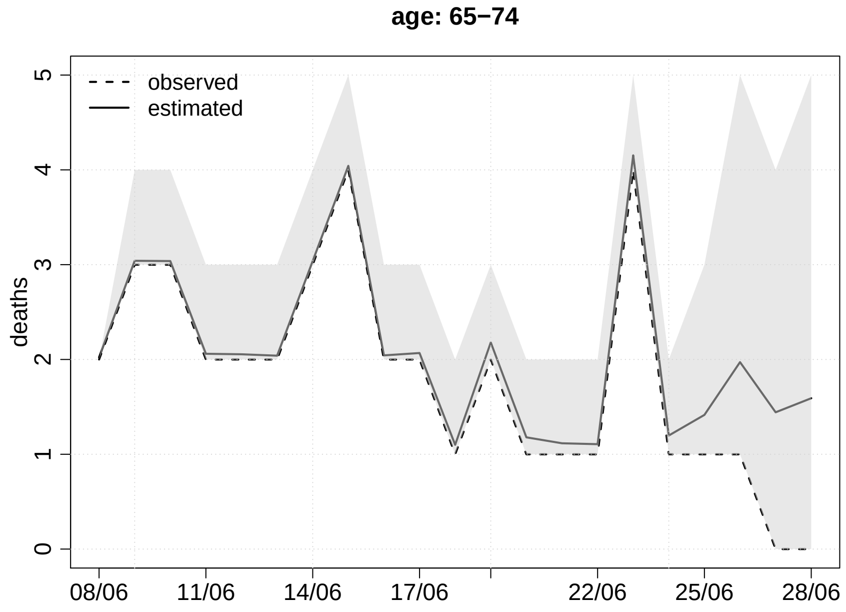

Figure S48: Estimates of the numbers of deaths occurring on each of the last 21 days (solid line) in the 65–74 stratum for Southeast, with numbers of deaths on each day reported by 29th June (broken line). Posterior 95% CIs are shown by shaded region.

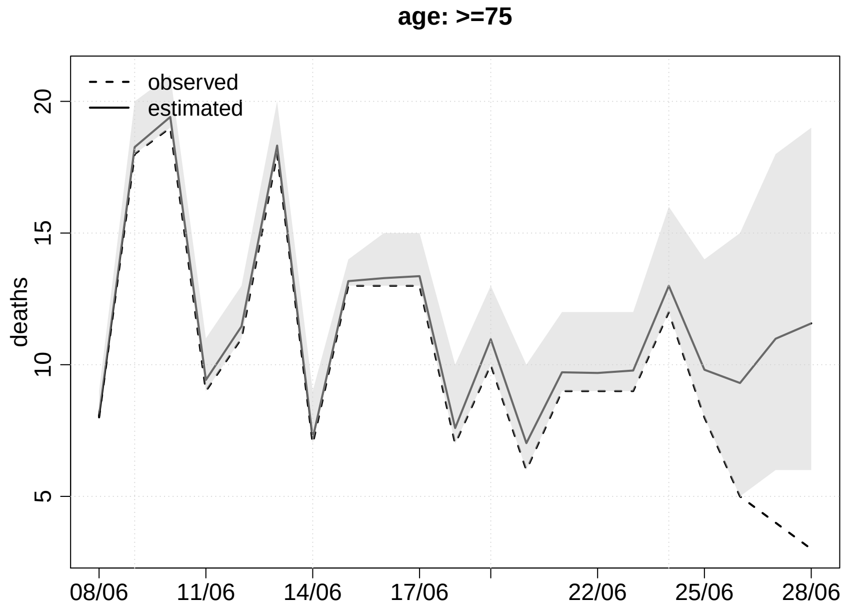

Figure S49: Estimates of the numbers of deaths occurring on each of the last 21 days (solid line) in the ≥ 75 stratum for Southeast, with numbers of deaths on each day reported by 29th June (broken line). Posterior 95% CIs are shown by shaded region.

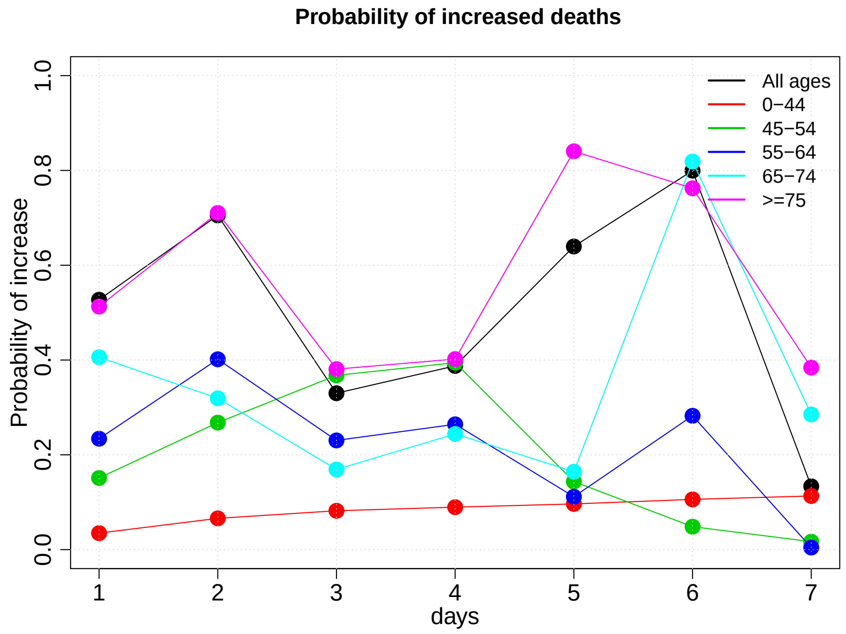

Figure S50: Posterior probabilities that the number of deaths in Southeast in the most recent x days was greater than the number in the preceding x days ($x = 1, \ldots 7$)

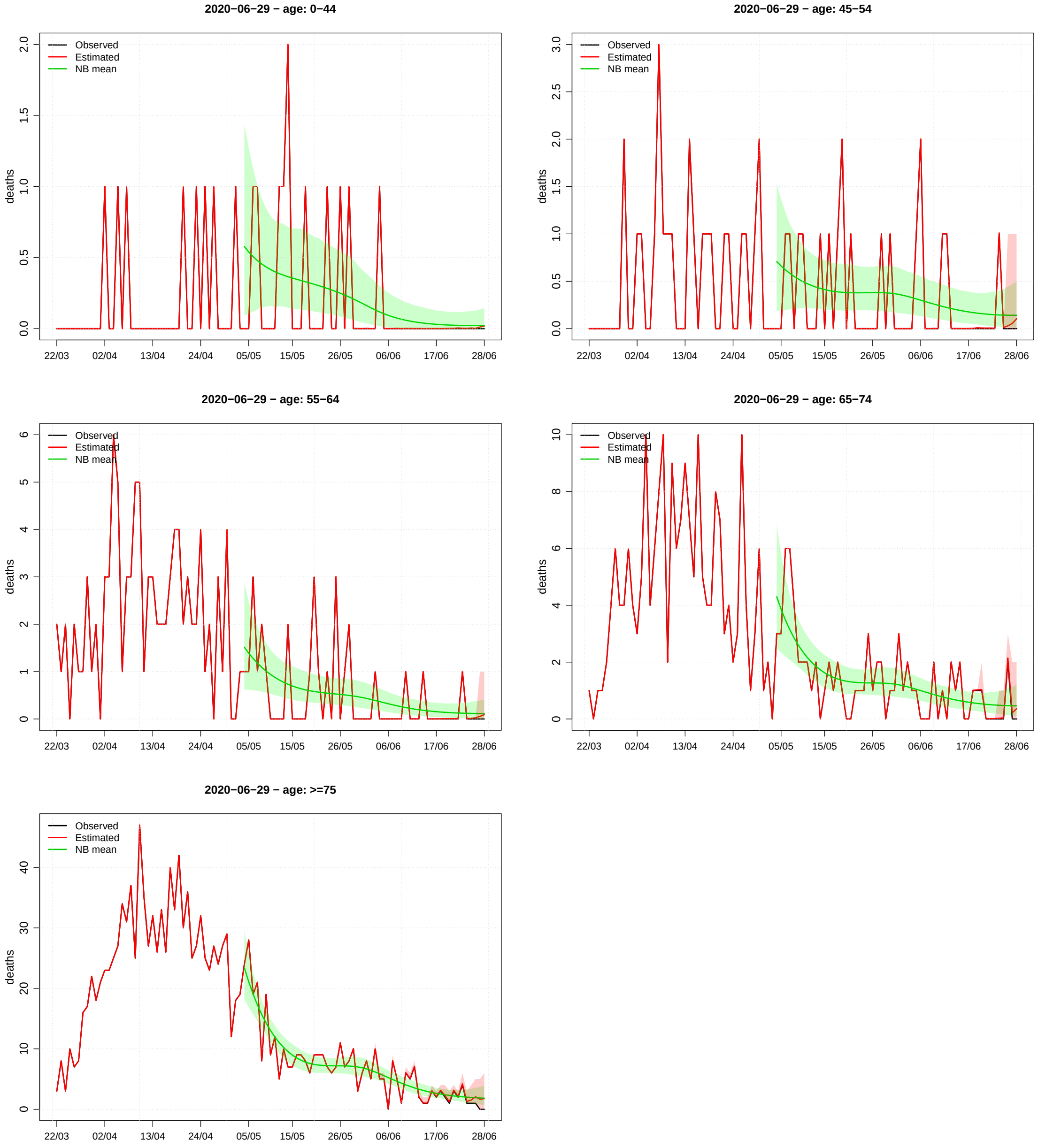

Figure S51: Estimates of the numbers of deaths occurring on each day in Southwest (red line), with numbers of deaths on each day reported by 29th June (black line). Posterior 95% CIs are shown by shaded red region. The green line shows the posterior mean of λ_{st}, the expected number of deaths. Posterior 95% CIs for this expected number are shown by shaded green region.

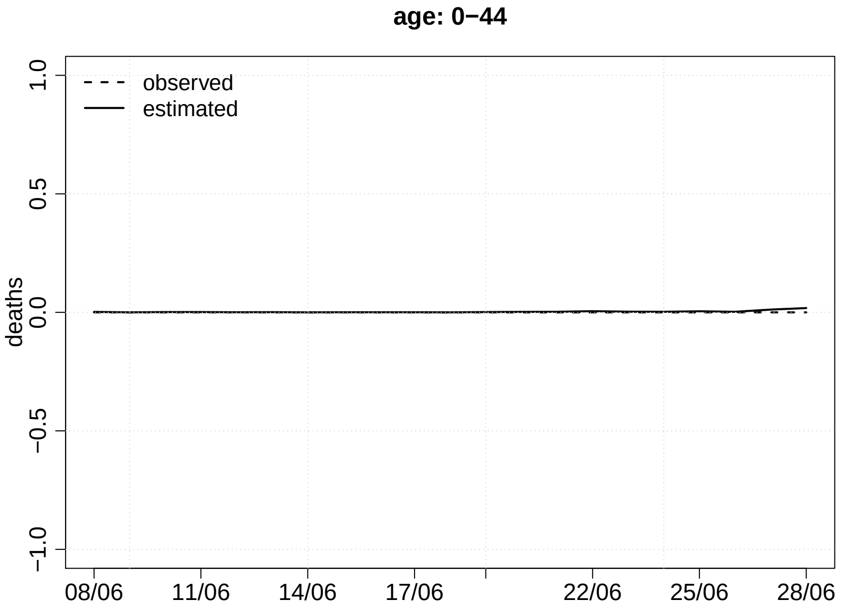

Figure S52: Estimates of the numbers of deaths occurring on each of the last 21 days (solid line) in the 0–44 stratum for Southwest, with numbers of deaths on each day reported by 29th June (broken line). Posterior 95% CIs are shown by shaded region.

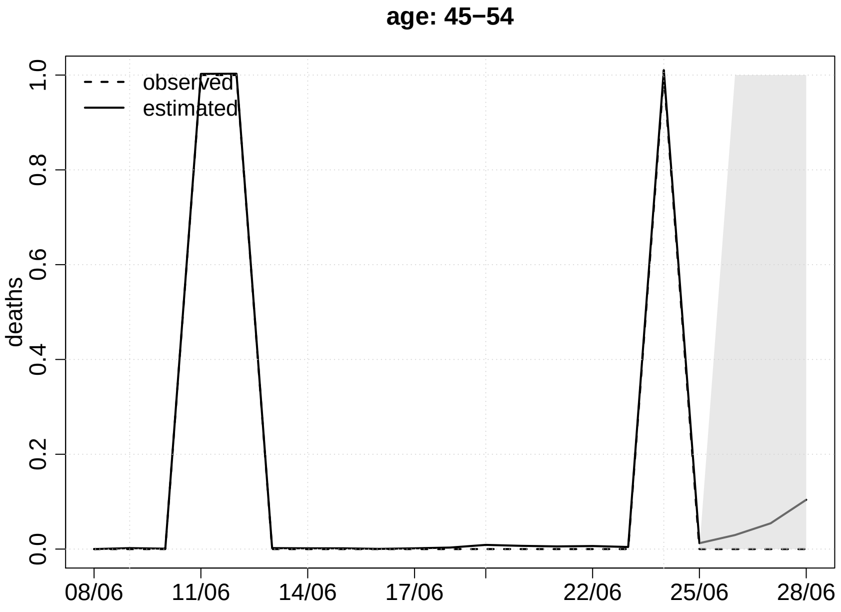

Figure S53: Estimates of the numbers of deaths occurring on each of the last 21 days (solid line) in the 45–54 stratum for Southwest, with numbers of deaths on each day reported by 29th June (broken line). Posterior 95% CIs are shown by shaded region.

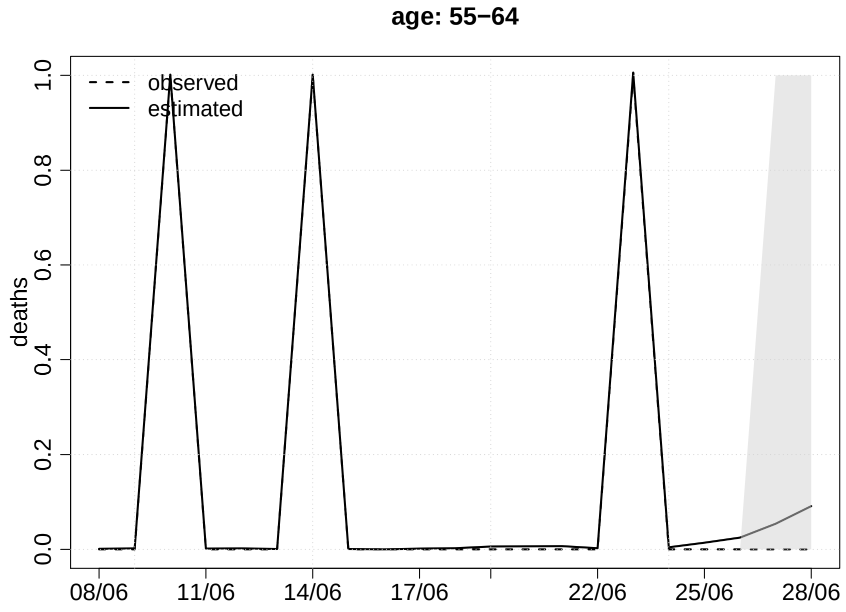

Figure S54: Estimates of the numbers of deaths occurring on each of the last 21 days (solid line) in the 55–64 stratum for Southwest, with numbers of deaths on each day reported by 29th June (broken line). Posterior 95% CIs are shown by shaded region.

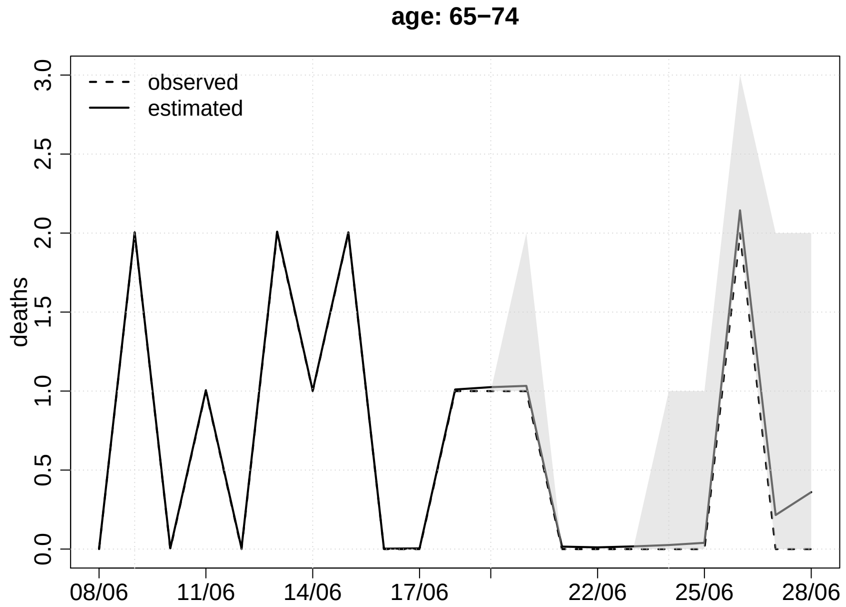

Figure S55: Estimates of the numbers of deaths occurring on each of the last 21 days (solid line) in the 65–74 stratum for Southwest, with numbers of deaths on each day reported by 29th June (broken line). Posterior 95% CIs are shown by shaded region.

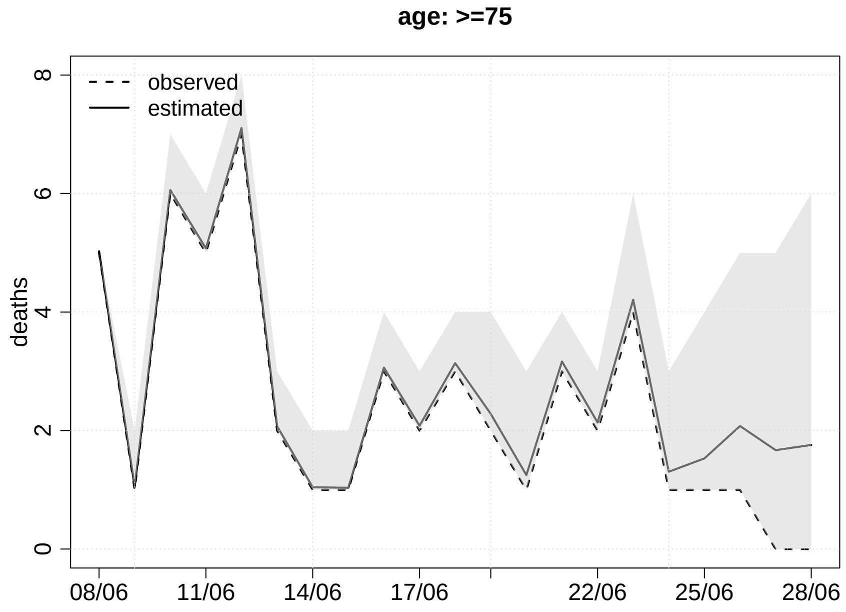

Figure S56: Estimates of the numbers of deaths occurring on each of the last 21 days (solid line) in the ≥ 75 stratum for Southwest, with numbers of deaths on each day reported by 29th June (broken line). Posterior 95% CIs are shown by shaded region.

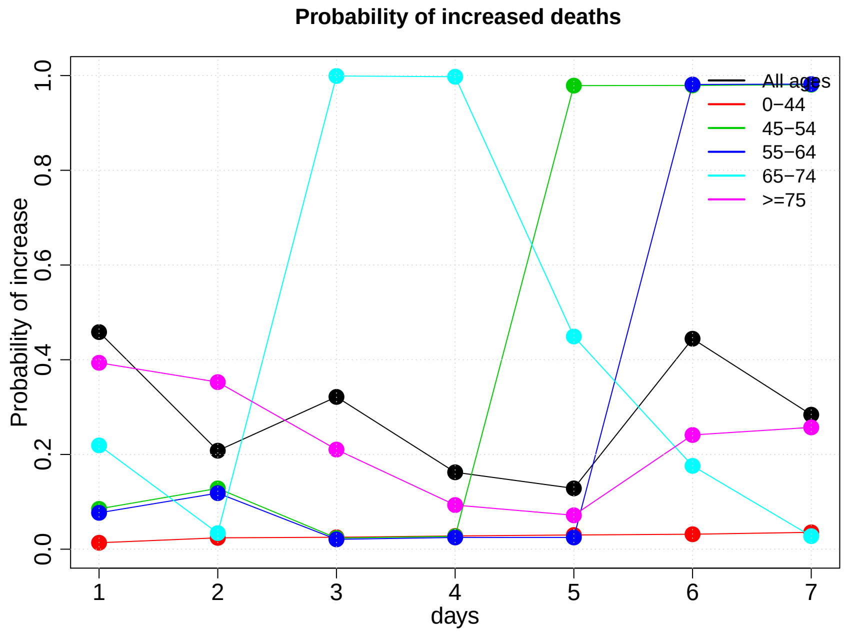

Figure S57: Posterior probabilities that the number of deaths in Southwest in the most recent x days was greater than the number in the preceding x days $(x = 1, \ldots 7)$

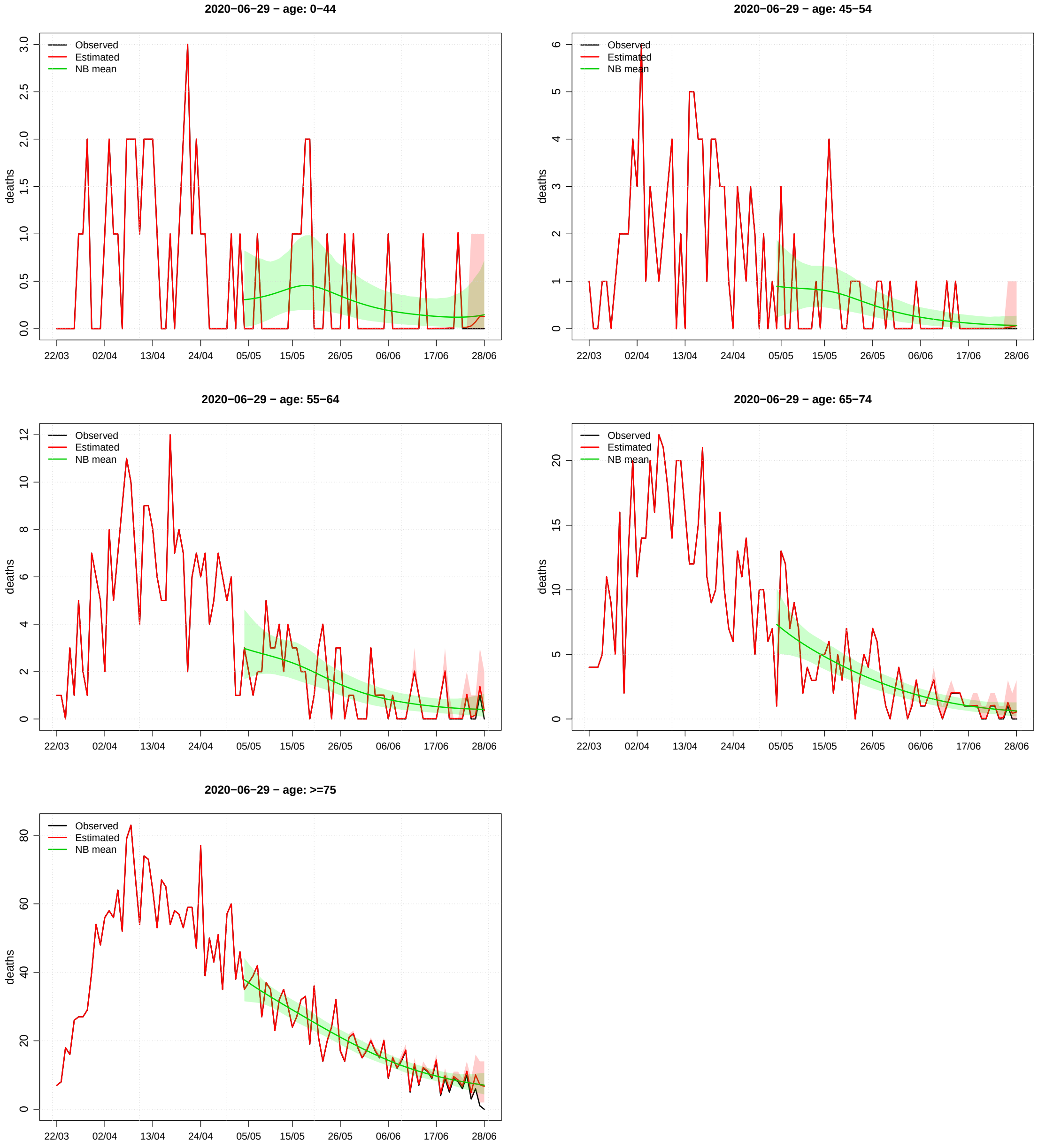

Figure S58: Estimates of the numbers of deaths occurring on each day in East (red line), with numbers of deaths on each day reported by 29th June (black line). Posterior 95% CIs are shown by shaded red region. The green line shows the posterior mean of λ_{st}, the expected number of deaths. Posterior 95% CIs for this expected number are shown by shaded green region.

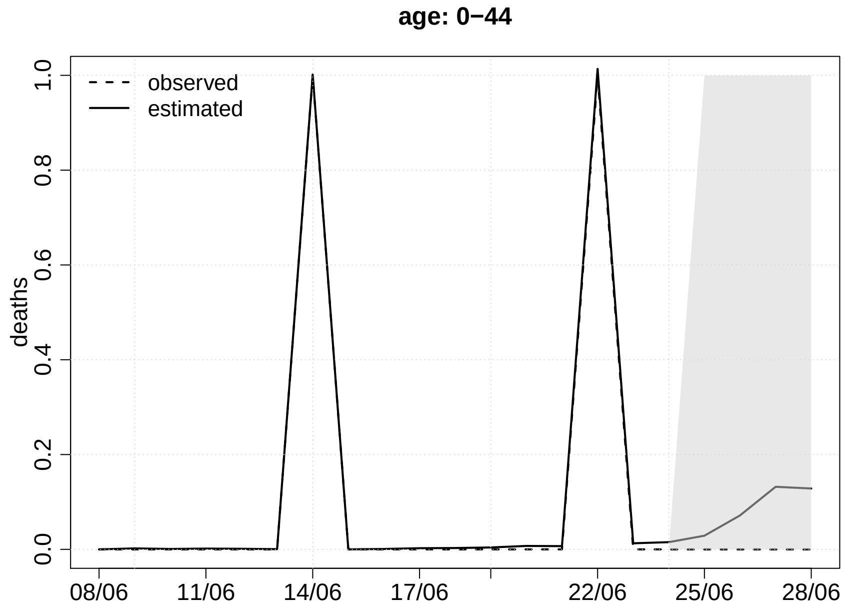

Figure S59: Estimates of the numbers of deaths occurring on each of the last 21 days (solid line) in the 0–44 stratum for East, with numbers of deaths on each day reported by 29th June (broken line). Posterior 95% CIs are shown by shaded region.

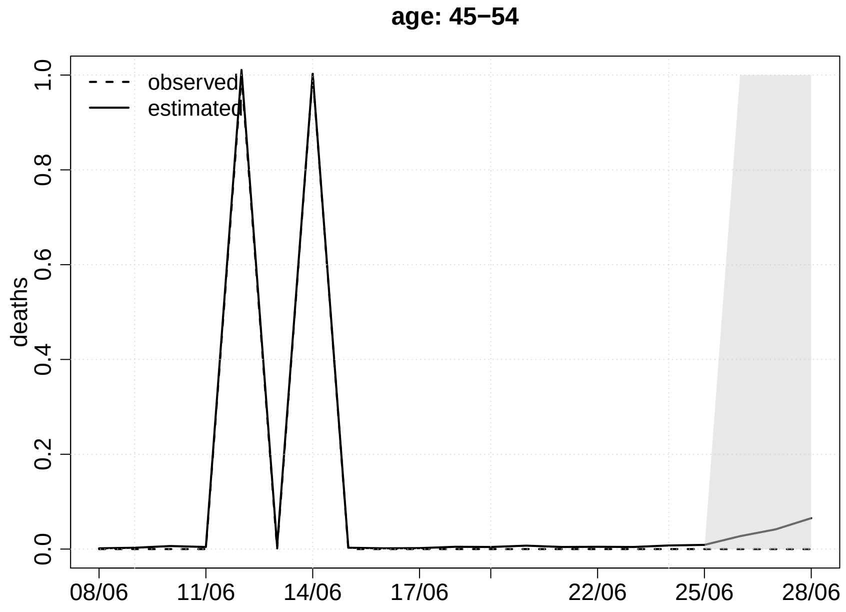

Figure S60: Estimates of the numbers of deaths occurring on each of the last 21 days (solid line) in the 45–54 stratum for East, with numbers of deaths on each day reported by 29th June (broken line). Posterior 95% CIs are shown by shaded region.

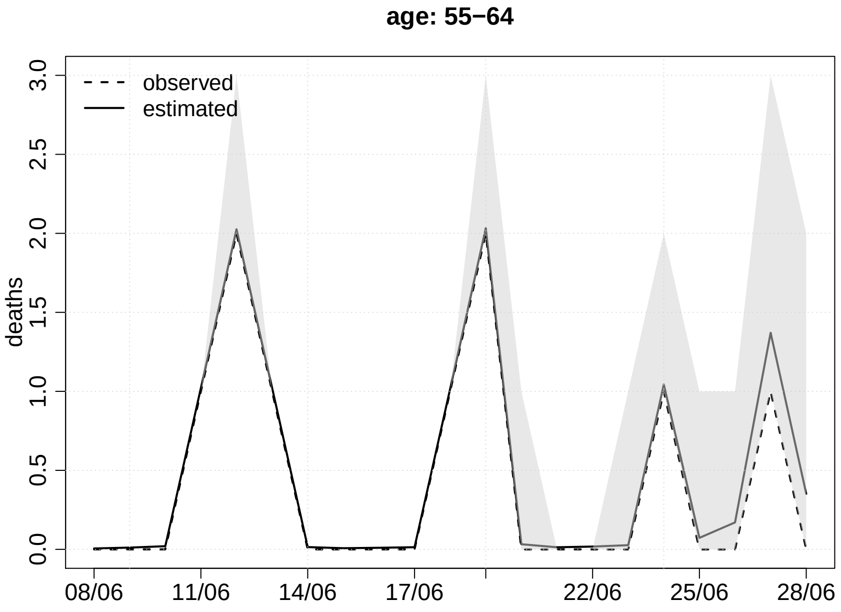

Figure S61: Estimates of the numbers of deaths occurring on each of the last 21 days (solid line) in the 55–64 stratum for East, with numbers of deaths on each day reported by 29th June (broken line). Posterior 95% CIs are shown by shaded region.

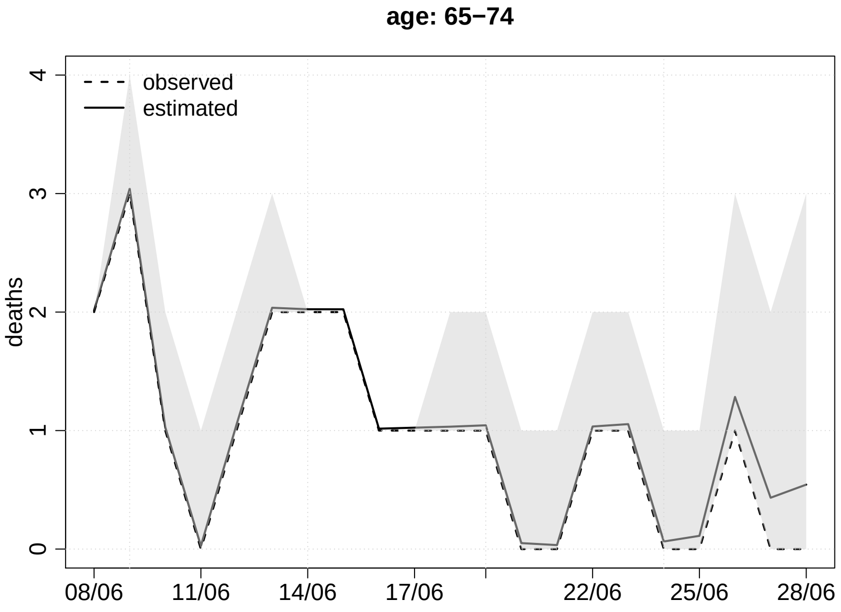

Figure S62: Estimates of the numbers of deaths occurring on each of the last 21 days (solid line) in the 65–74 stratum for East, with numbers of deaths on each day reported by 29th June (broken line). Posterior 95% CIs are shown by shaded region.

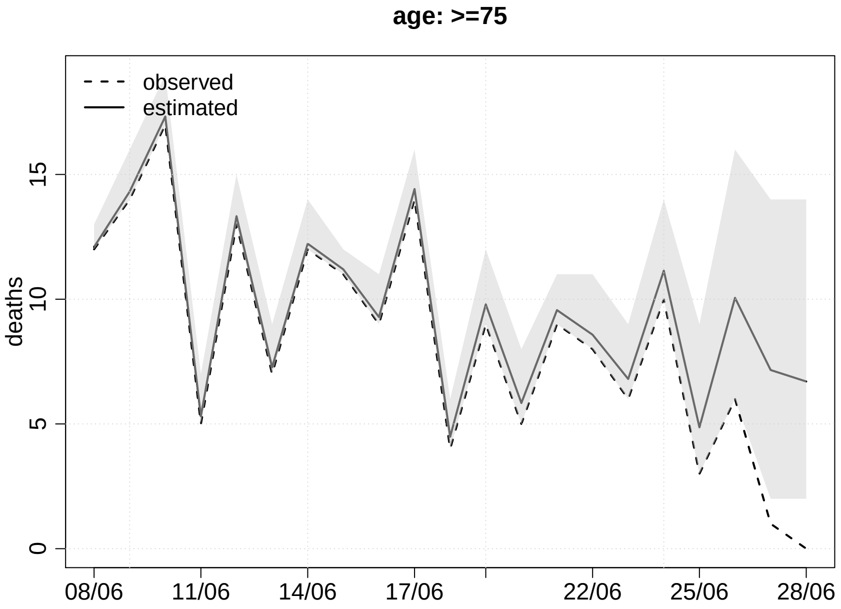

Figure S63: Estimates of the numbers of deaths occurring on each of the last 21 days (solid line) in the ≥ 75 stratum for East, with numbers of deaths on each day reported by 29th June (broken line). Posterior 95% CIs are shown by shaded region.

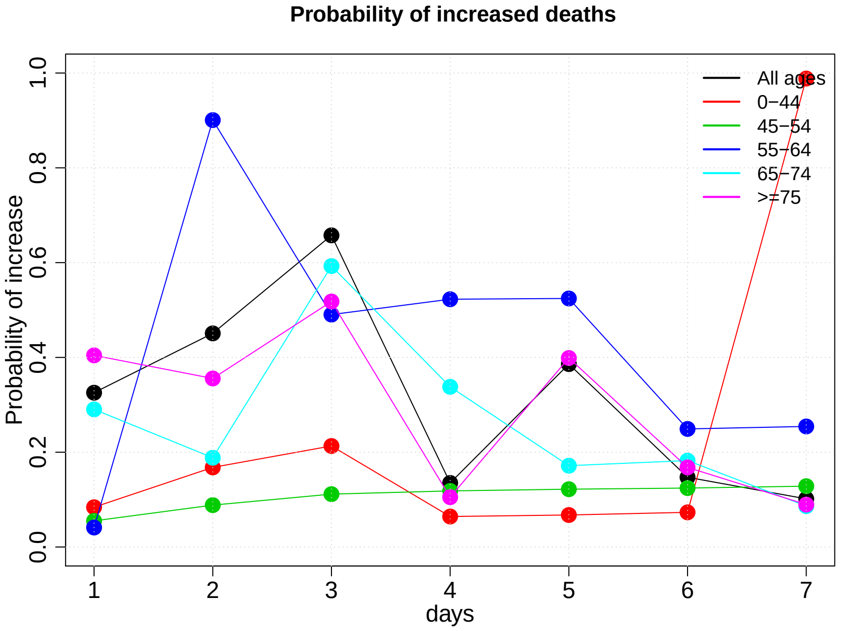

Figure S64: Posterior probabilities that the number of deaths in East in the most recent x days was greater than the number in the preceding x days ($x = 1, \ldots 7$)

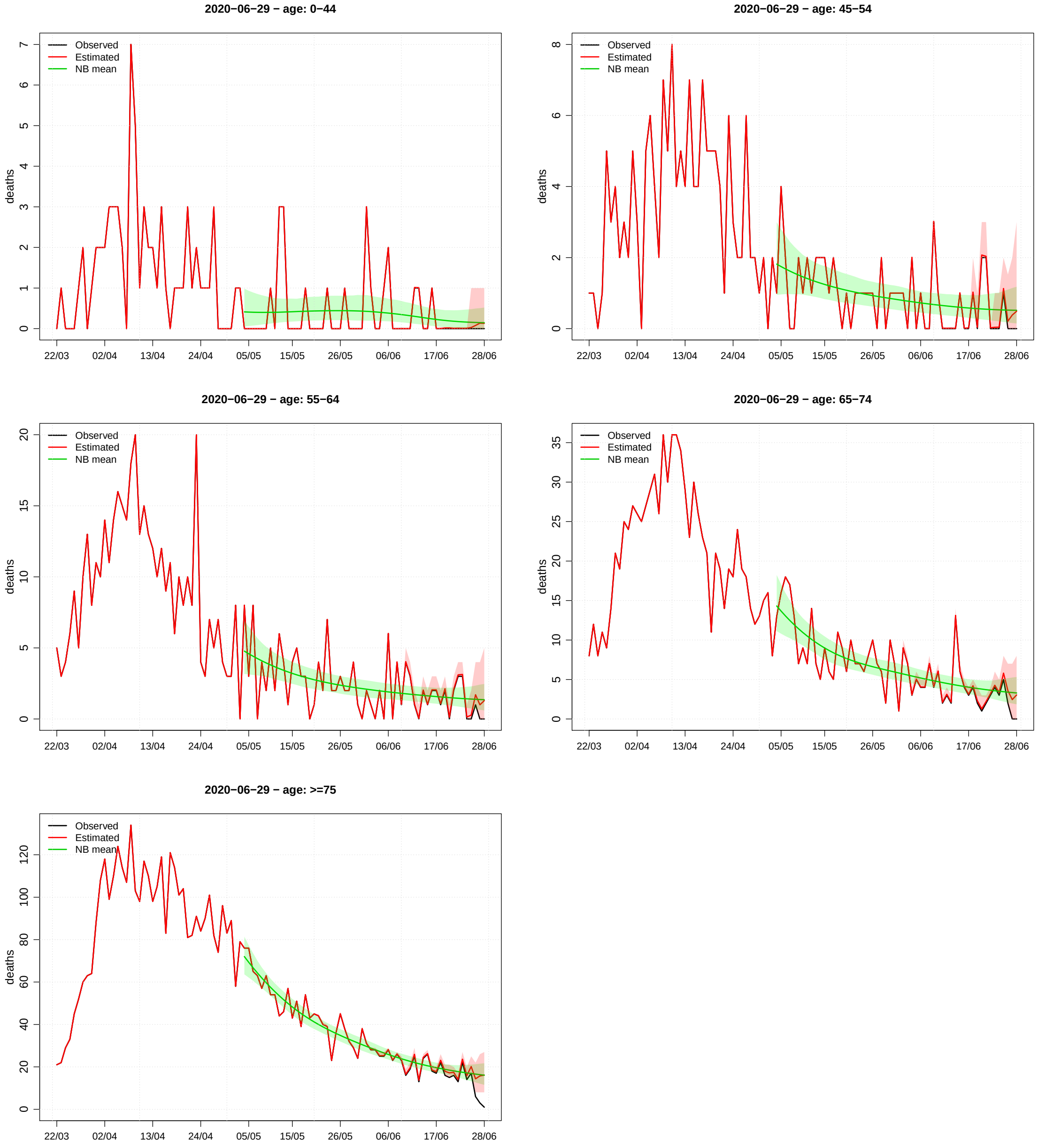

Figure S65: Estimates of the numbers of deaths occurring on each day in Midland (red line), with numbers of deaths on each day reported by 29th June (black line). Posterior 95% CIs are shown by shaded red region. The green line shows the posterior mean of λ_{st}, the expected number of deaths. Posterior 95% CIs for this expected number are shown by shaded green region.

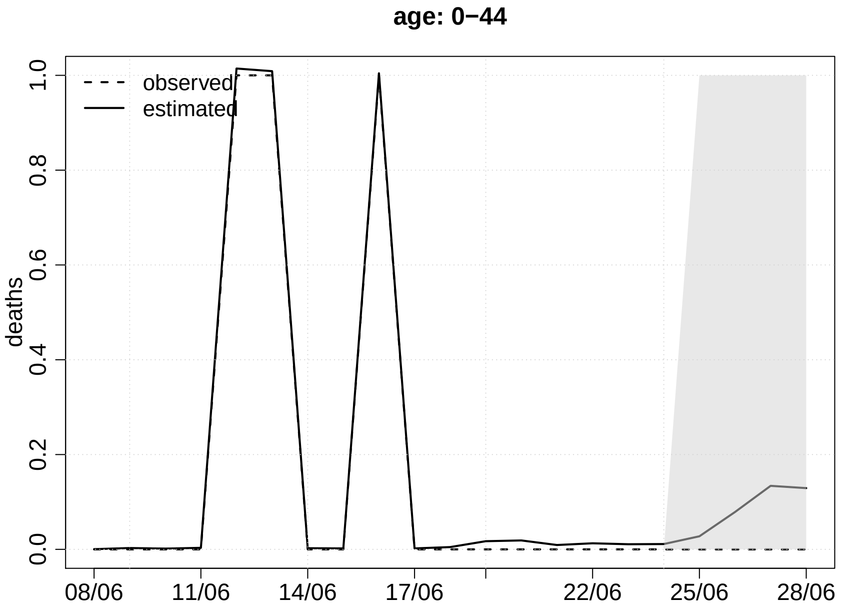

Figure S66: Estimates of the numbers of deaths occurring on each of the last 21 days (solid line) in the 0–44 stratum for Midlands, with numbers of deaths on each day reported by 29th June (broken line). Posterior 95% CIs are shown by shaded region.

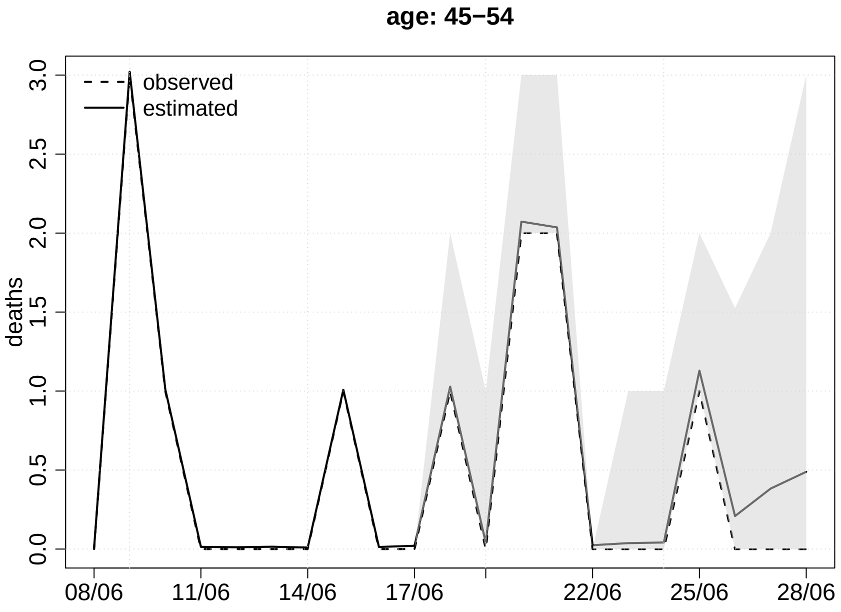

Figure S67: Estimates of the numbers of deaths occurring on each of the last 21 days (solid line) in the 45–54 stratum for Midlands, with numbers of deaths on each day reported by 29th June (broken line). Posterior 95% CIs are shown by shaded region.

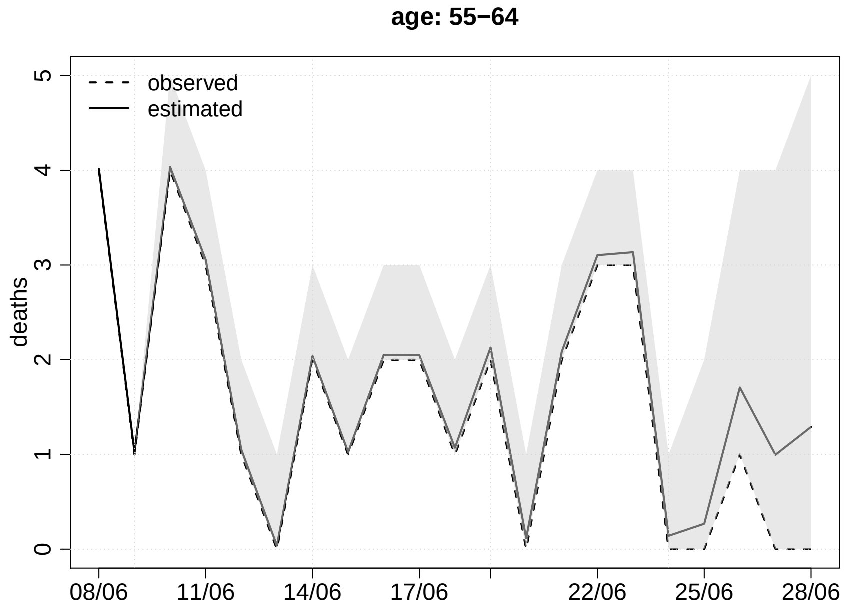

Figure S68: Estimates of the numbers of deaths occurring on each of the last 21 days (solid line) in the 55–64 stratum for Midlands, with numbers of deaths on each day reported by 29th June (broken line). Posterior 95% CIs are shown by shaded region.

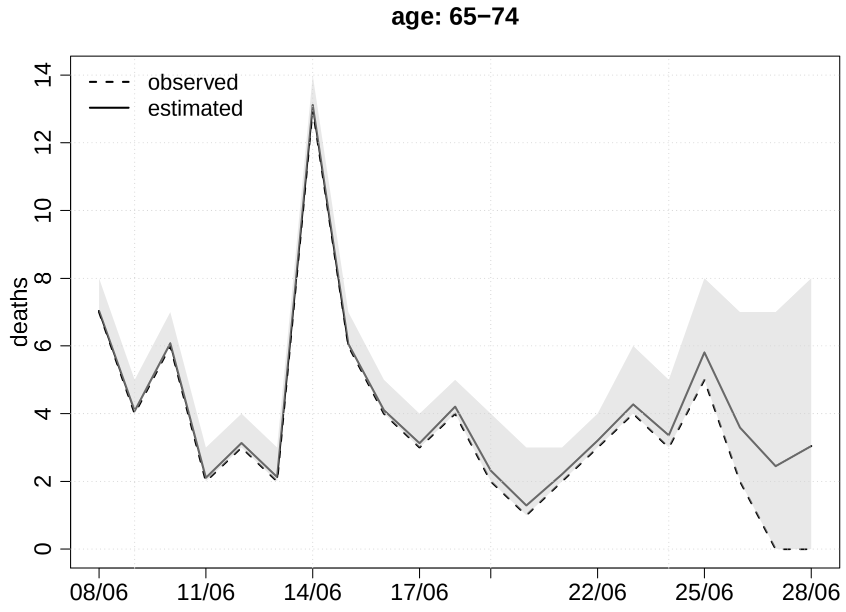

Figure S69: Estimates of the numbers of deaths occurring on each of the last 21 days (solid line) in the 65–74 stratum for Midlands, with numbers of deaths on each day reported by 29th June (broken line). Posterior 95% CIs are shown by shaded region.

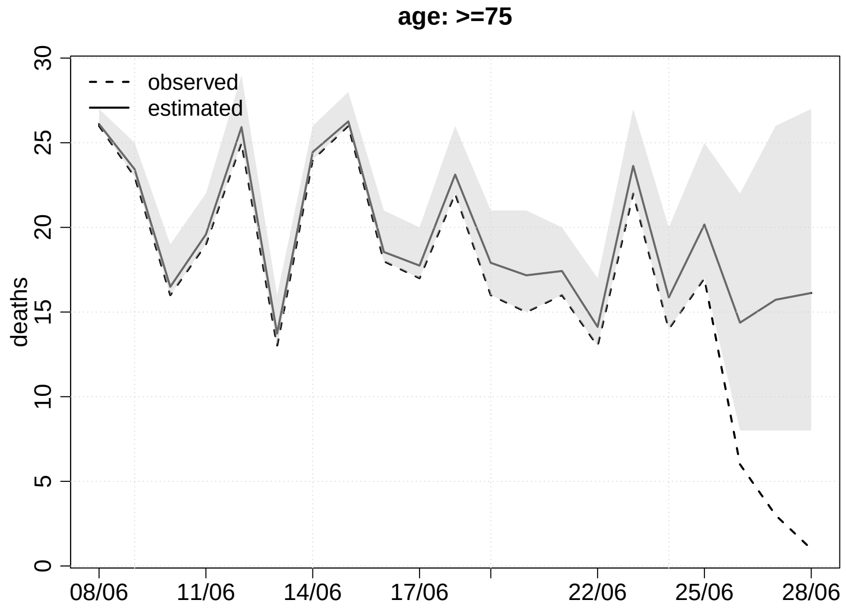

Figure S70: Estimates of the numbers of deaths occurring on each of the last 21 days (solid line) in the ≥ 75 stratum for Midlands, with numbers of deaths on each day reported by 29th June (broken line). Posterior 95% CIs are shown by shaded region.

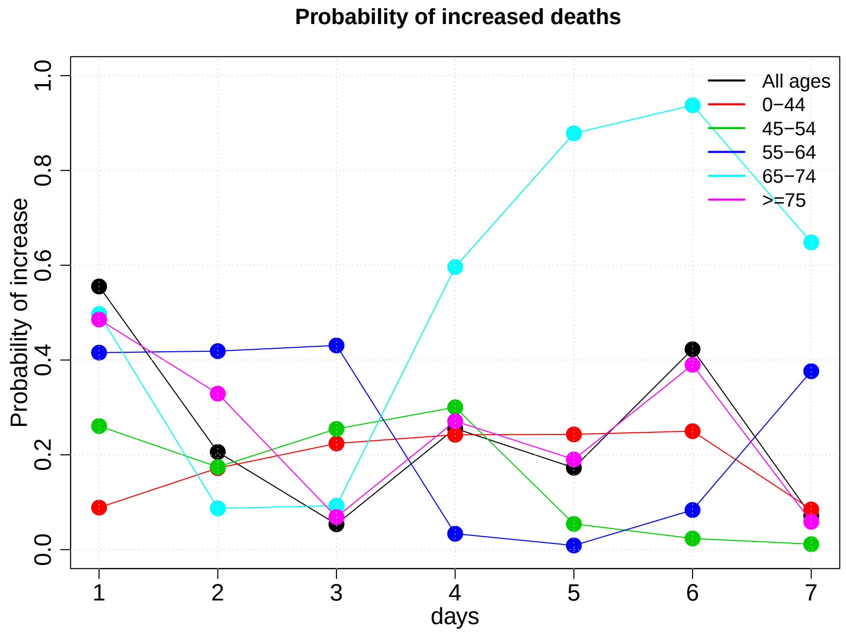

Figure S71: Posterior probabilities that the number of deaths in Midlands in the most recent x days was greater than the number in the preceding x days ($x = 1, \ldots 7$)

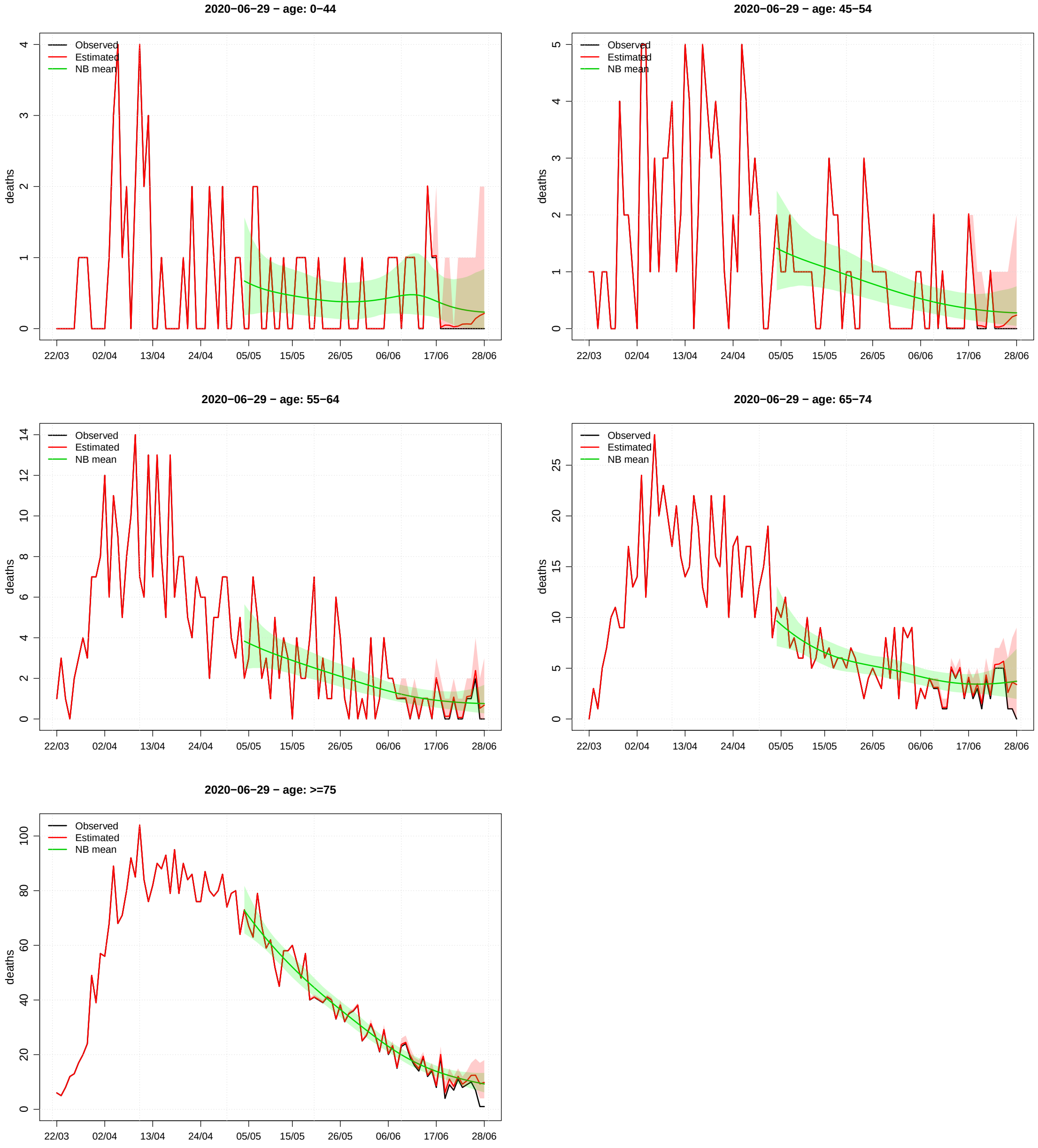

Figure S72: Estimates of the numbers of deaths occurring on each day in Northeast (red line), with numbers of deaths on each day reported by 29th June (black line). Posterior 95% CIs are shown by shaded red region. The green line shows the posterior mean of λ_{st}, the expected number of deaths. Posterior 95% CIs for this expected number are shown by shaded green region.

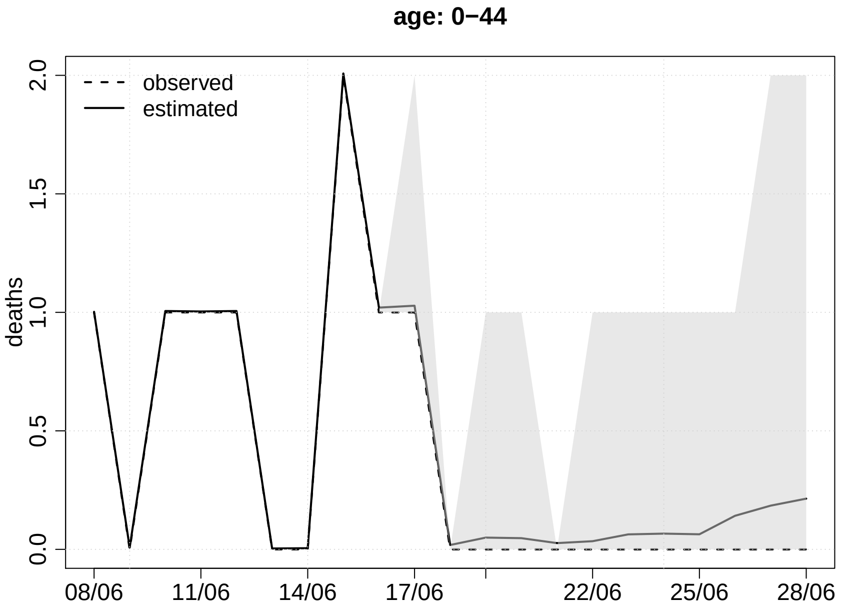

Figure S73: Estimates of the numbers of deaths occurring on each of the last 21 days (solid line) in the 0–44 stratum for Northeast, with numbers of deaths on each day reported by 29th June (broken line). Posterior 95% CIs are shown by shaded region.

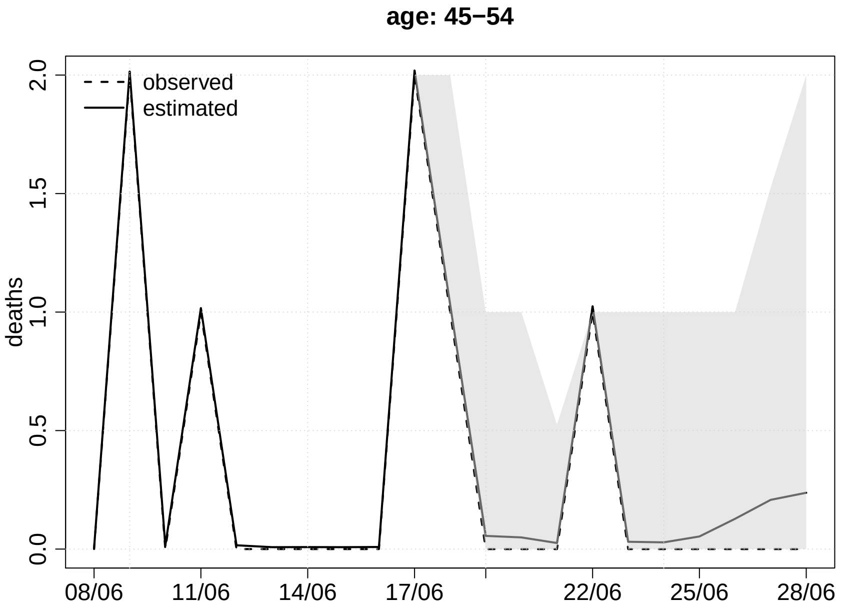

Figure S74: Estimates of the numbers of deaths occurring on each of the last 21 days (solid line) in the 45–54 stratum for Northeast, with numbers of deaths on each day reported by 29th June (broken line). Posterior 95% CIs are shown by shaded region.

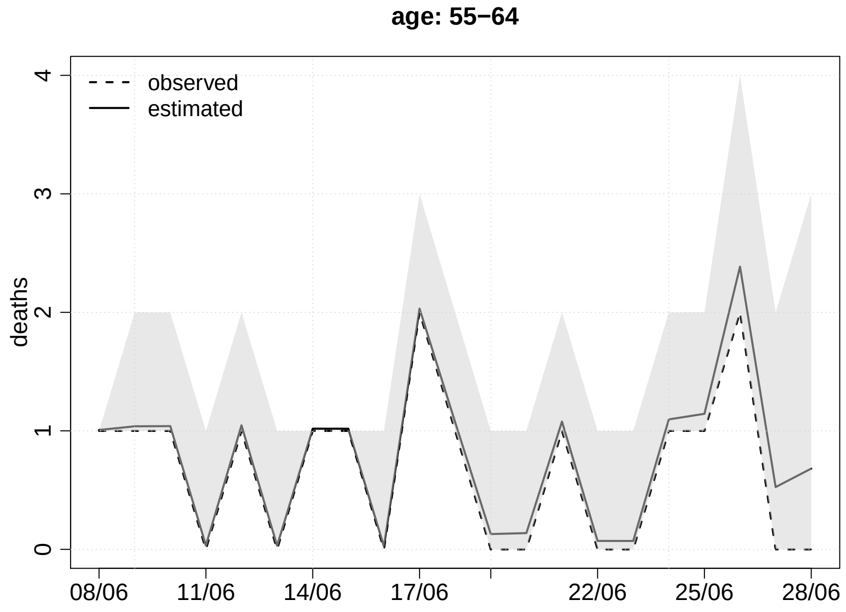

Figure S75: Estimates of the numbers of deaths occurring on each of the last 21 days (solid line) in the 55–64 stratum for Northeast, with numbers of deaths on each day reported by 29th June (broken line). Posterior 95% CIs are shown by shaded region.

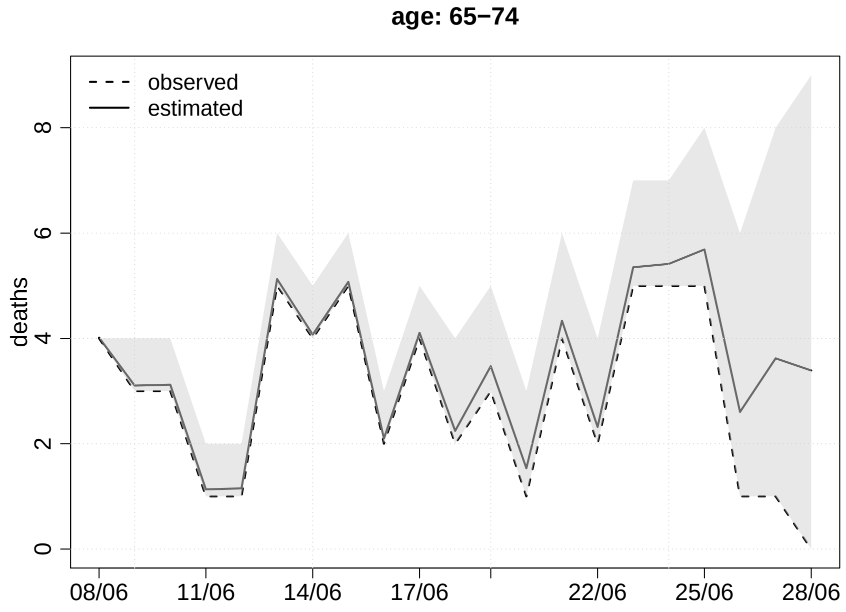

Figure S76: Estimates of the numbers of deaths occurring on each of the last 21 days (solid line) in the 65–74 stratum for Northeast, with numbers of deaths on each day reported by 29th June (broken line). Posterior 95% CIs are shown by shaded region.

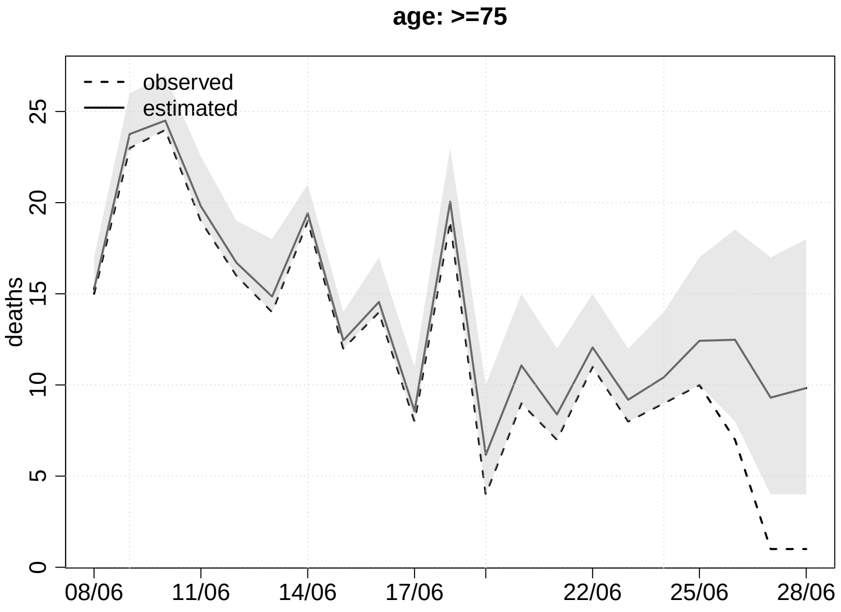

Figure S77: Estimates of the numbers of deaths occurring on each of the last 21 days (solid line) in the ≥ 75 stratum for Northeast, with numbers of deaths on each day reported by 29th June (broken line). Posterior 95% CIs are shown by shaded region.

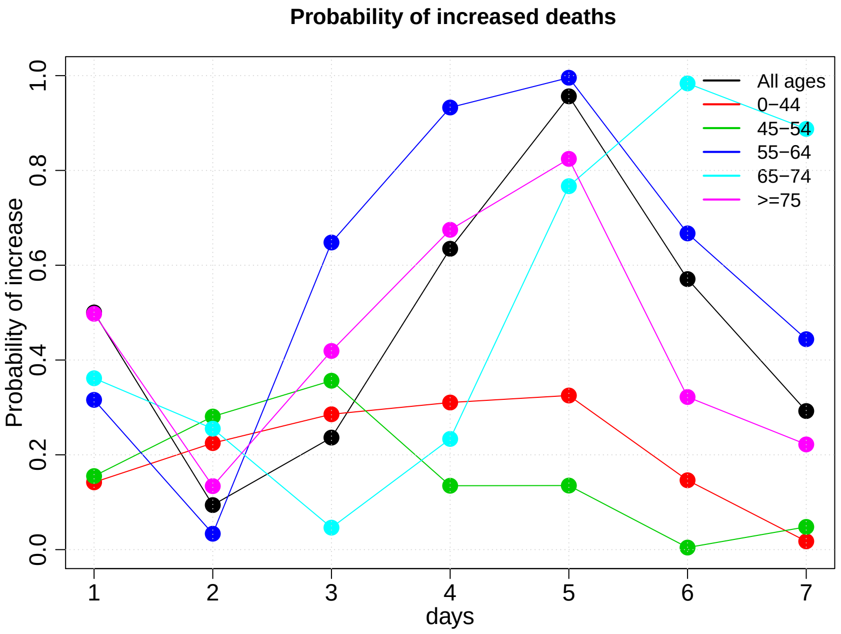

Figure S78: Posterior probabilities that the number of deaths in Northeast in the most recent x days was greater than the number in the preceding x days ($x = 1, \ldots 7$)

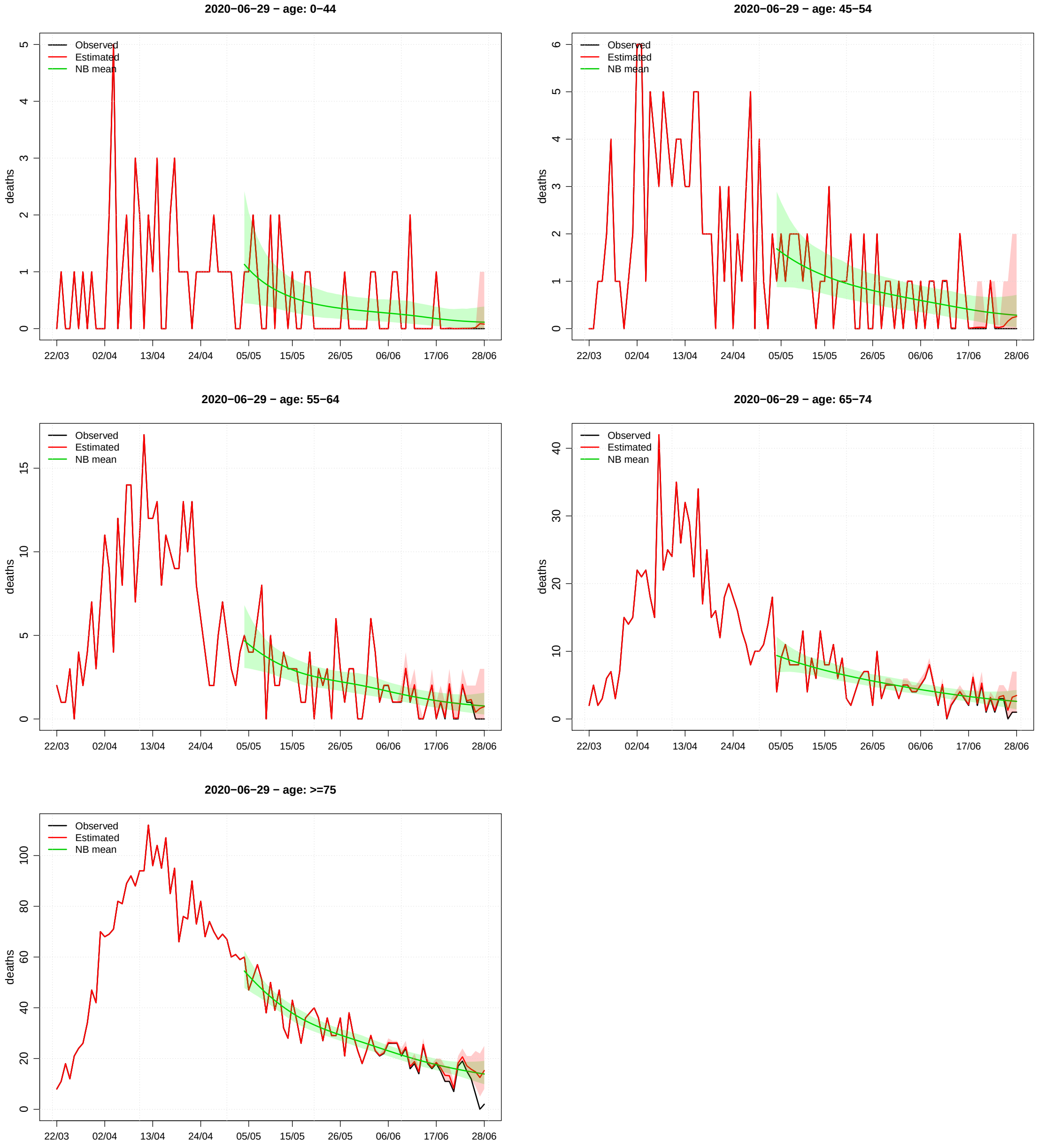

Figure S79: Estimates of the numbers of deaths occurring on each day in Northwest (red line), with numbers of deaths on each day reported by 29th June (black line). Posterior 95% CIs are shown by shaded red region. The green line shows the posterior mean of λ_{st}, the expected number of deaths. Posterior 95% CIs for this expected number are shown by shaded green region.

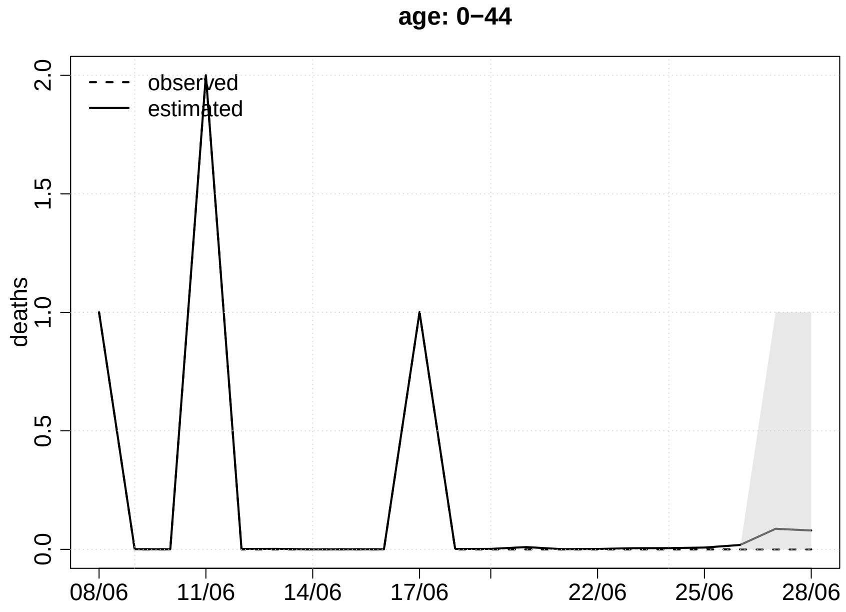

Figure S80: Estimates of the numbers of deaths occurring on each of the last 21 days (solid line) in the 0–44 stratum for Northwest, with numbers of deaths on each day reported by 29th June (broken line). Posterior 95% CIs are shown by shaded region.

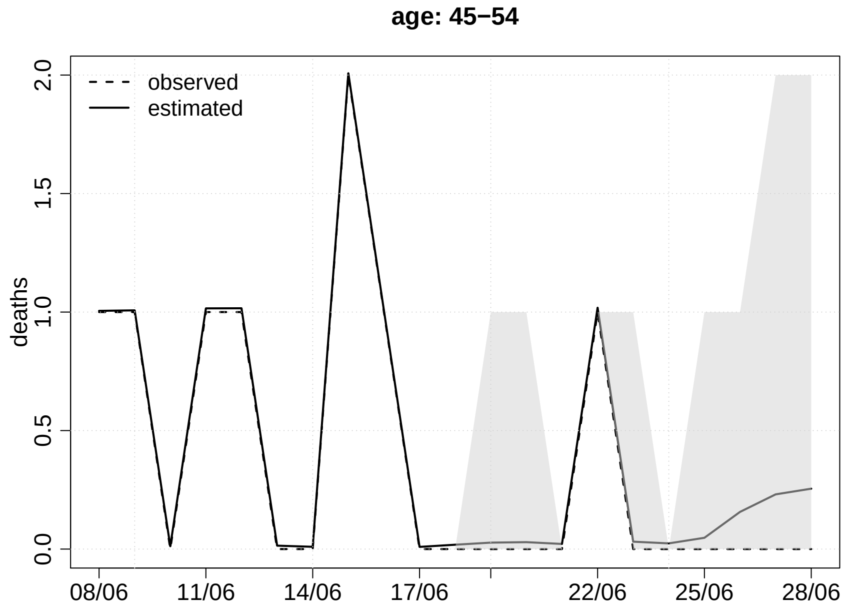

Figure S81: Estimates of the numbers of deaths occurring on each of the last 21 days (solid line) in the 45–54 stratum for Northwest, with numbers of deaths on each day reported by 29th June (broken line). Posterior 95% CIs are shown by shaded region.

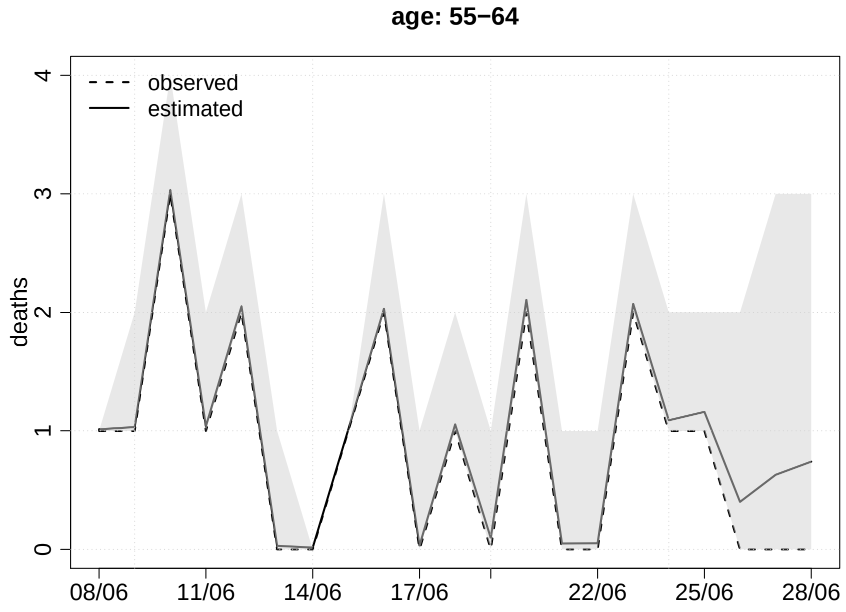

Figure S82: Estimates of the numbers of deaths occurring on each of the last 21 days (solid line) in the 55–64 stratum for Northwest, with numbers of deaths on each day reported by 29th June (broken line). Posterior 95% CIs are shown by shaded region.

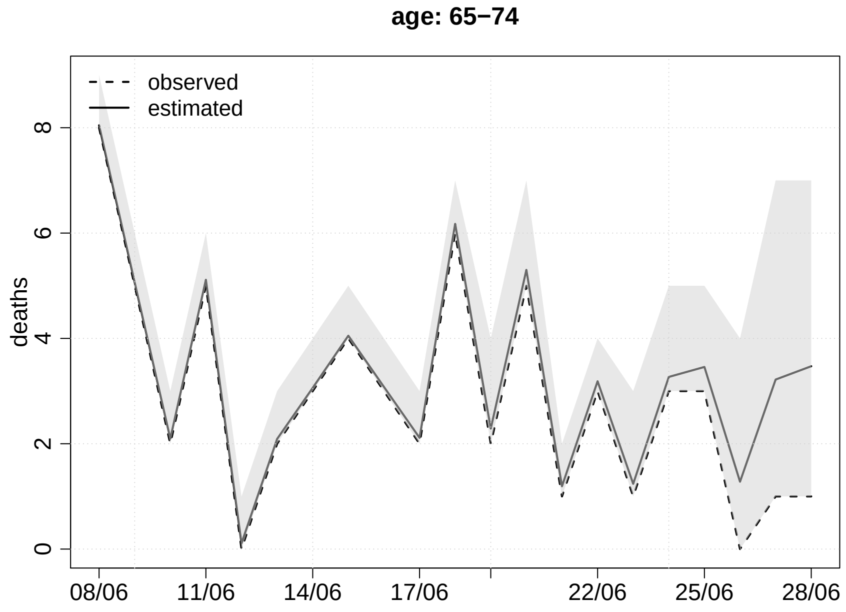

Figure S83: Estimates of the numbers of deaths occurring on each of the last 21 days (solid line) in the 65–74 stratum for Northwest, with numbers of deaths on each day reported by 29th June (broken line). Posterior 95% CIs are shown by shaded region.

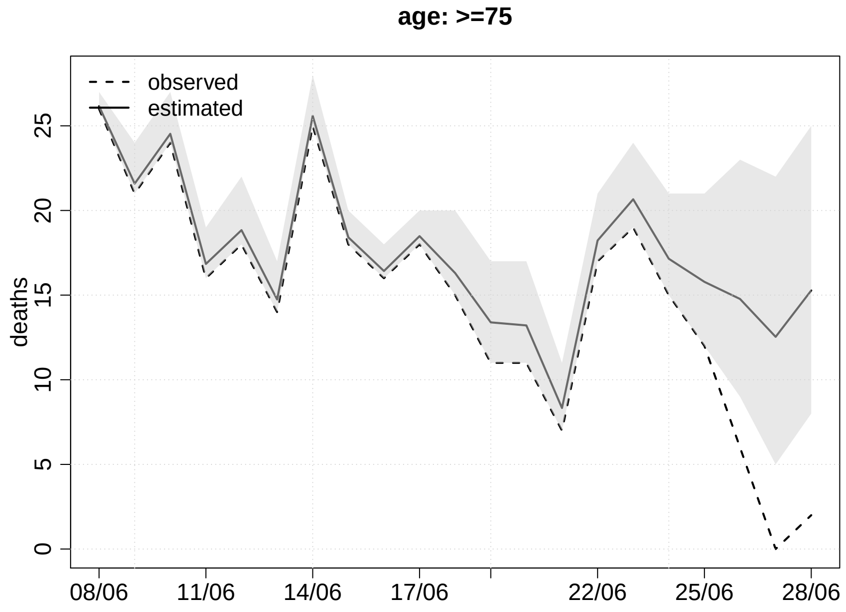

Figure S84: Estimates of the numbers of deaths occurring on each of the last 21 days (solid line) in the ≥ 75 stratum for Northwest, with numbers of deaths on each day reported by 29th June (broken line). Posterior 95% CIs are shown by shaded region.

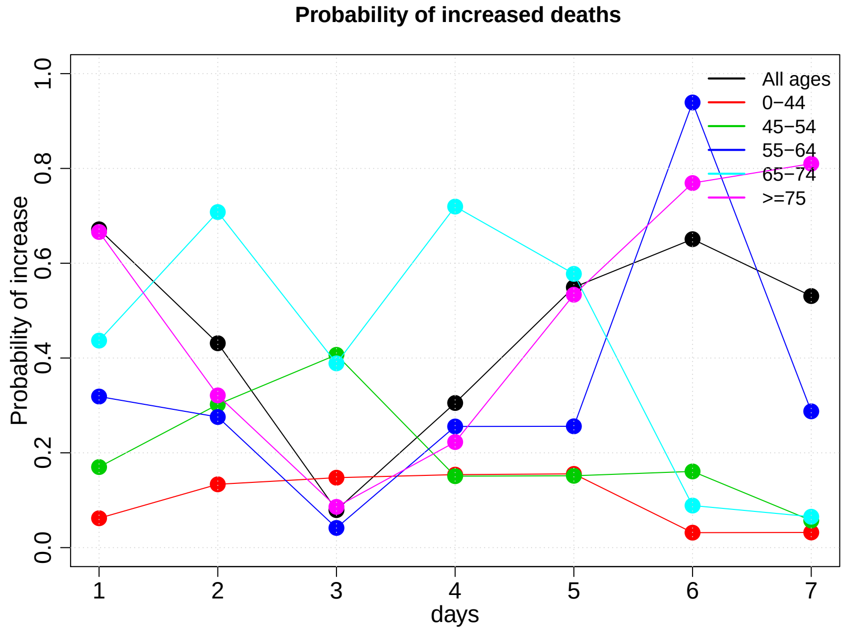

Figure S85: Posterior probabilities that the number of deaths in Northwest in the most recent x days was greater than the number in the preceding x days ($x = 1, \ldots 7$)

S6 Trace plots

Figures S86–S91 shows MCMC trace plots for Y_{st} for each stratum s and the most recent ten days from the analysis of the data from the whole of England available on 29th June.

Figures S92–S97 shows the corresponding trace plots from the analysis of the data from London.

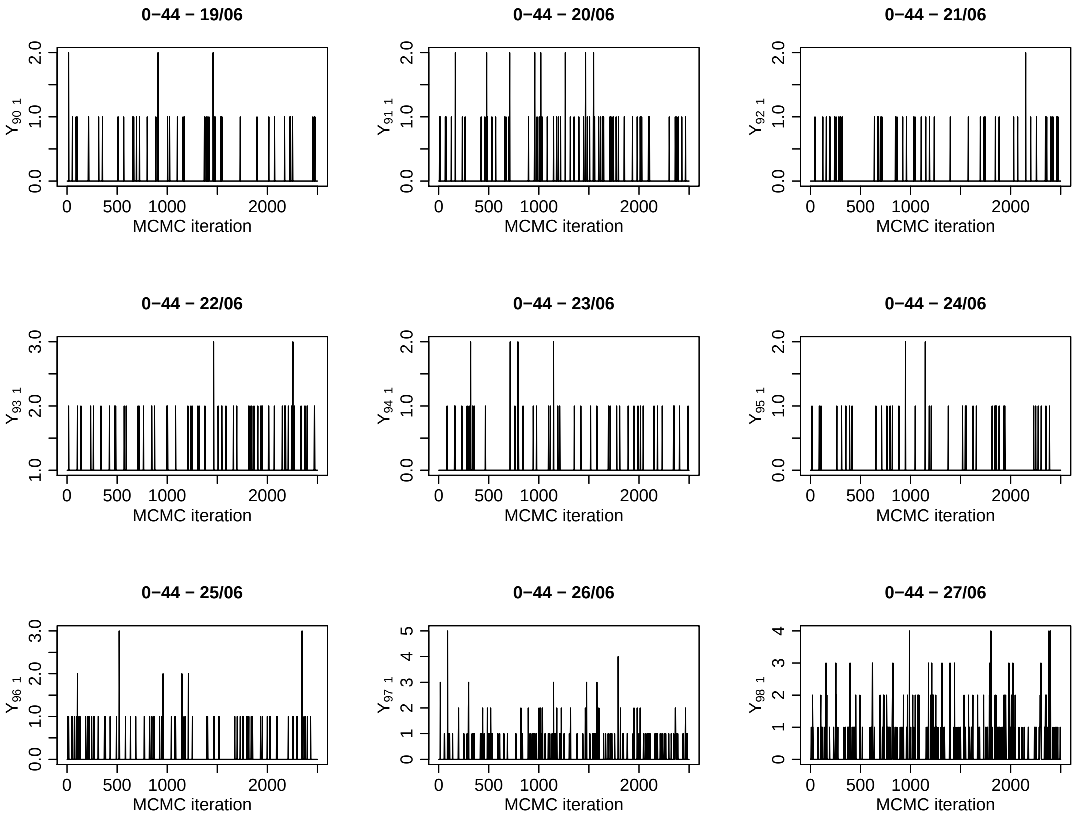

Figure S86: Trace plots for Y_{st} from analysis of England data

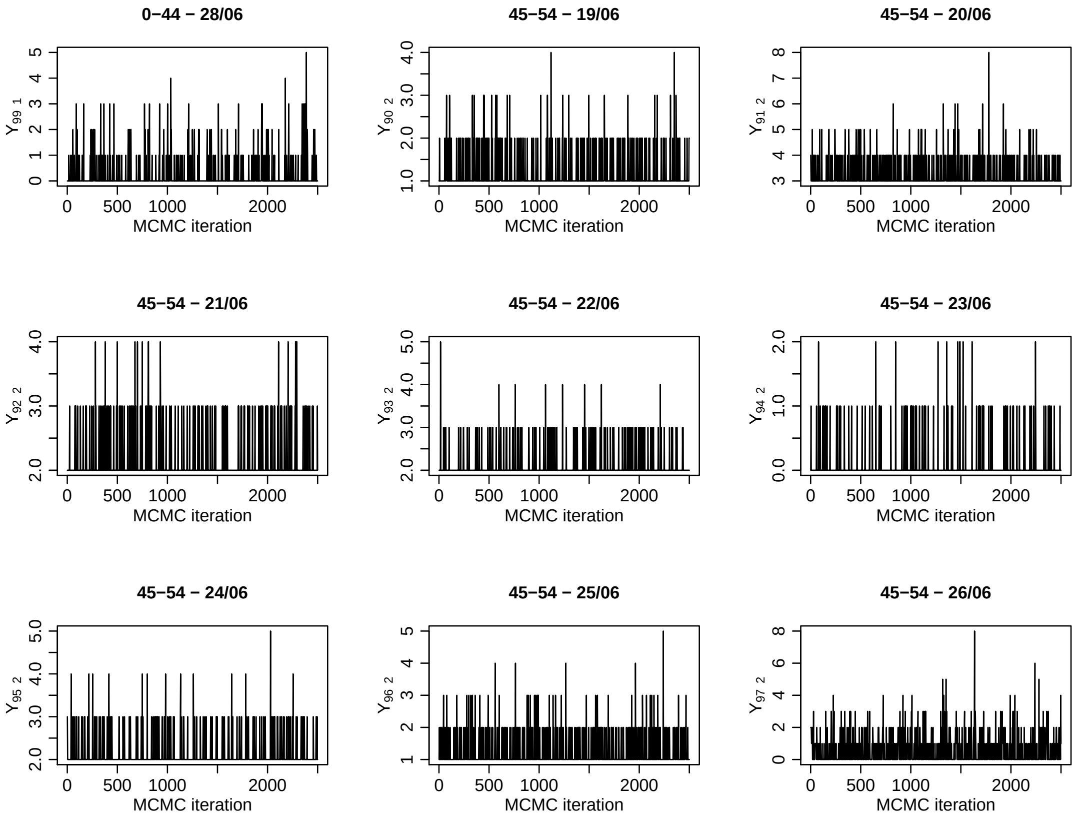

Figure S87: Trace plots for Y_{st} from analysis of England data

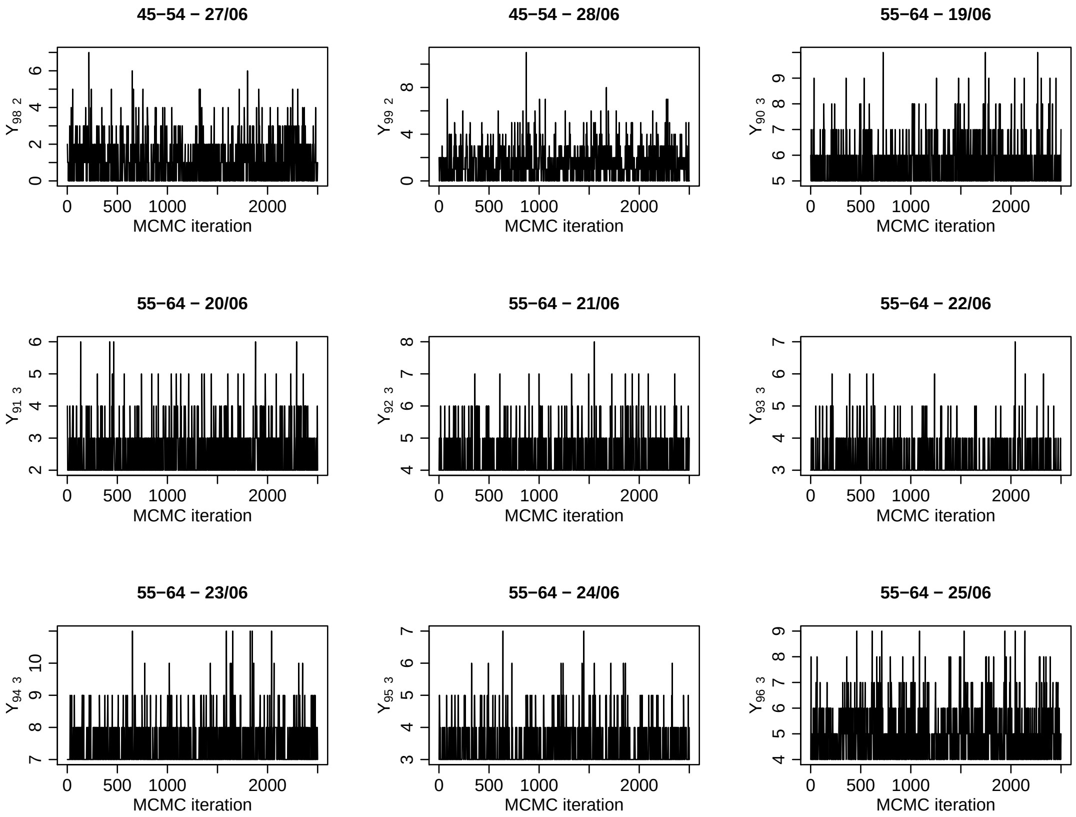

Figure S88: Trace plots for Y_{st} from analysis of England data

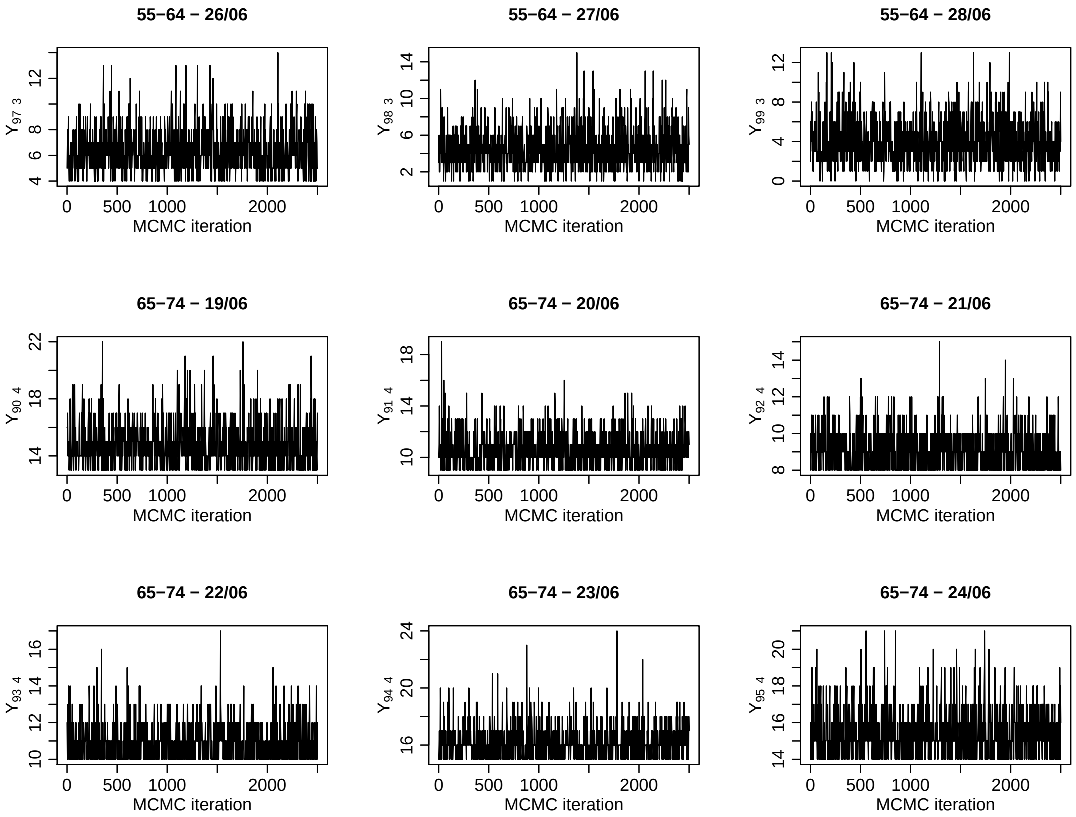

Figure S89: Trace plots for Y_{st} from analysis of England data

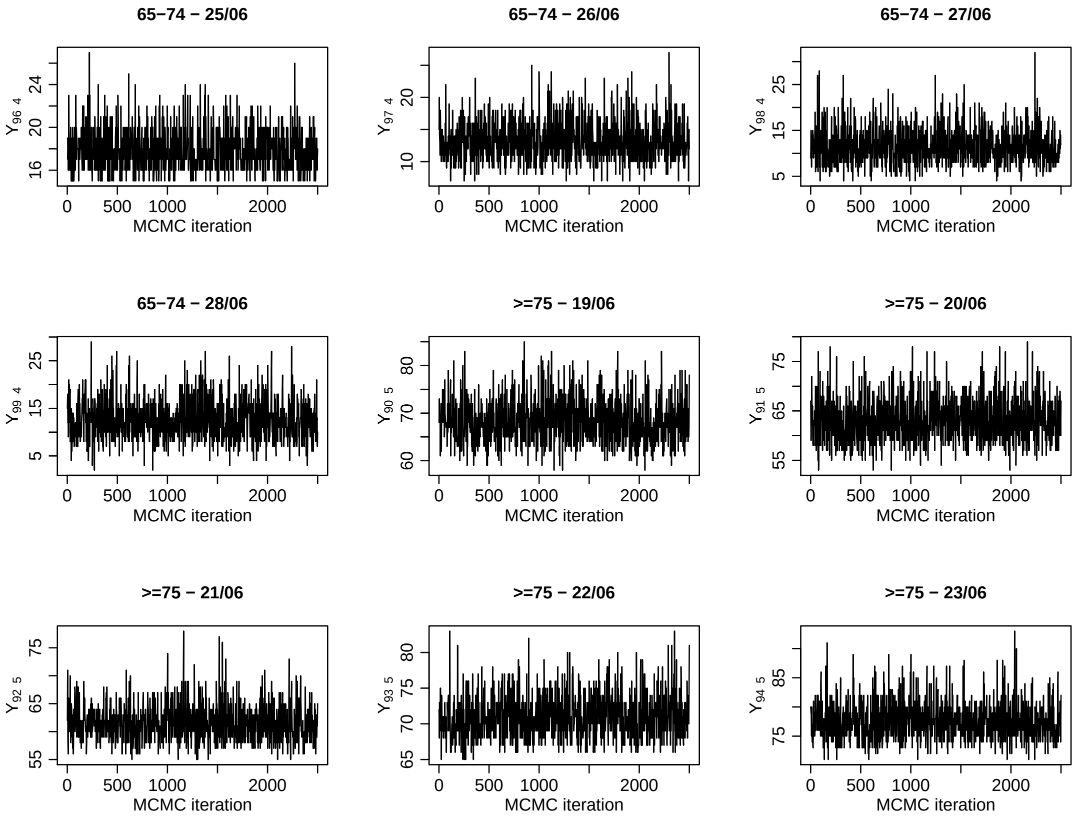

Figure S90: Trace plots for Y_{st} from analysis of England data

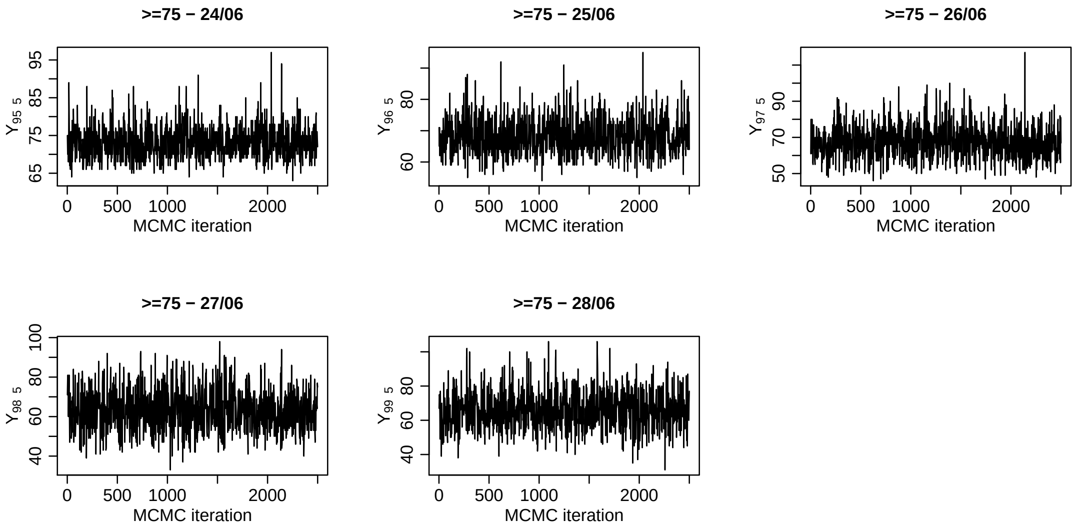

Figure S91: Trace plots for Y_{st} from analysis of England data

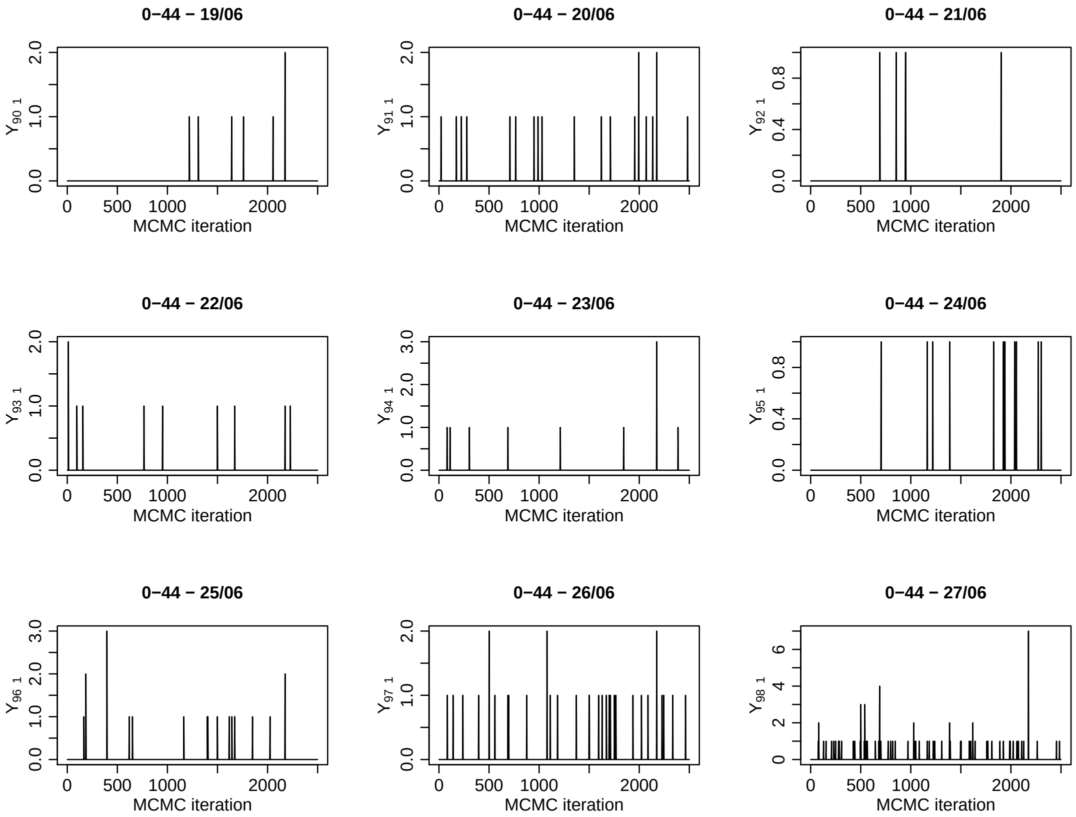

Figure S92: Trace plots for Y_{st} from analysis of London data

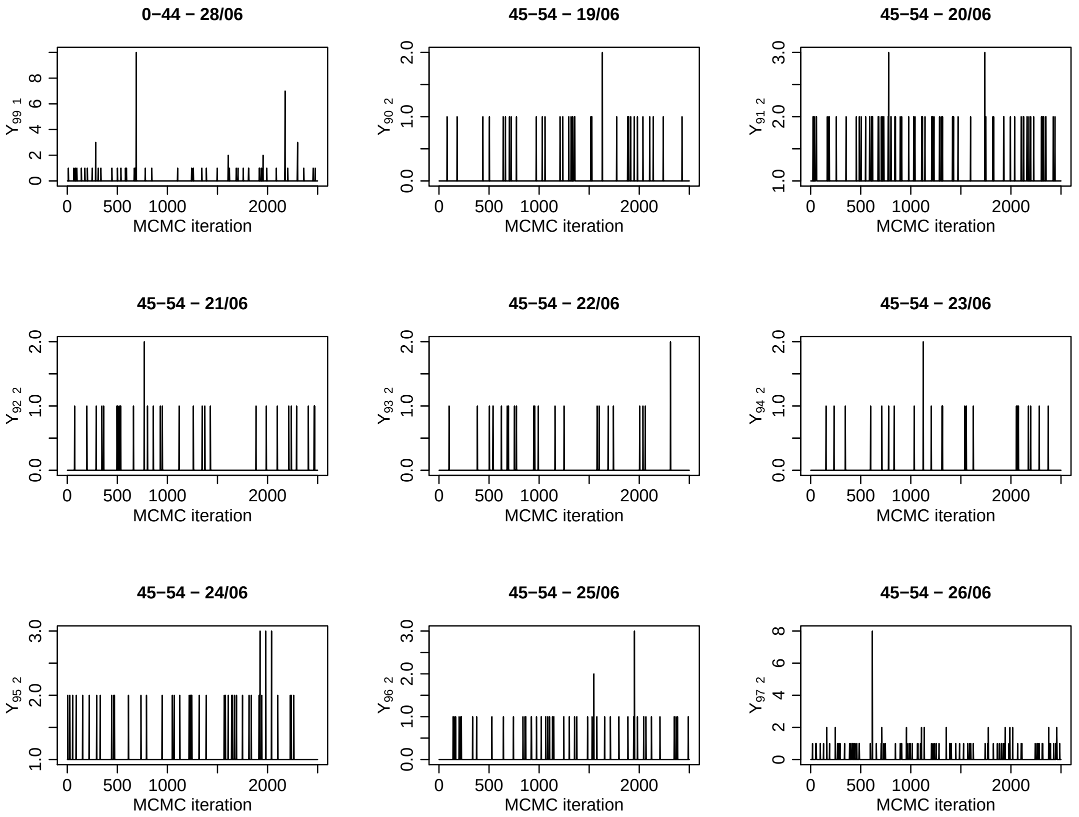

Figure S93: Trace plots for Y_{st} from analysis of London data

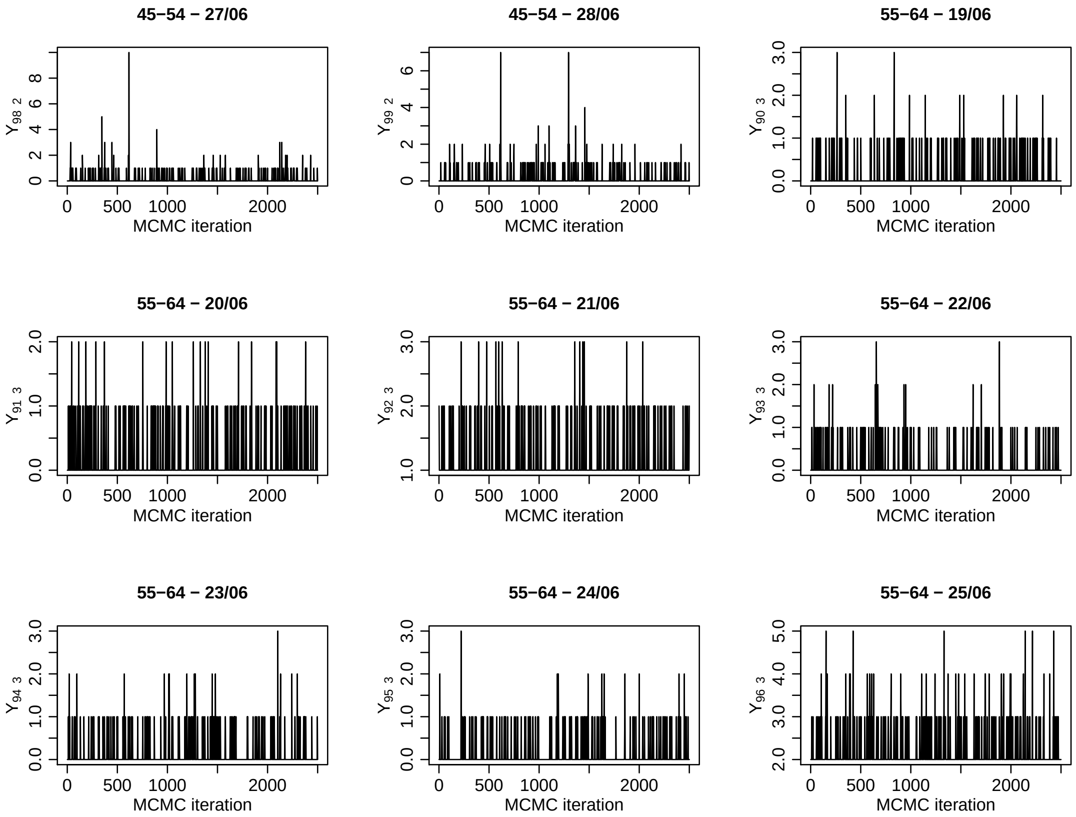

Figure S94: Trace plots for Y_{st} from analysis of London data

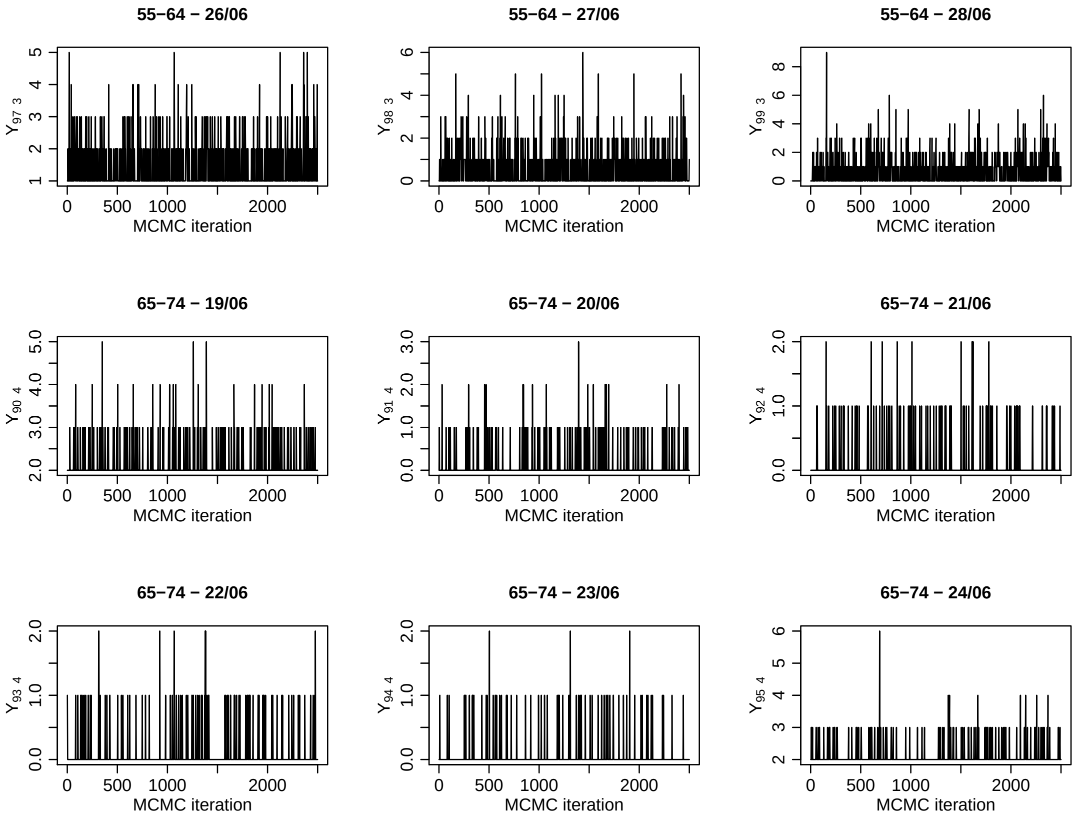

Figure S95: Trace plots for Y_{st} from analysis of London data

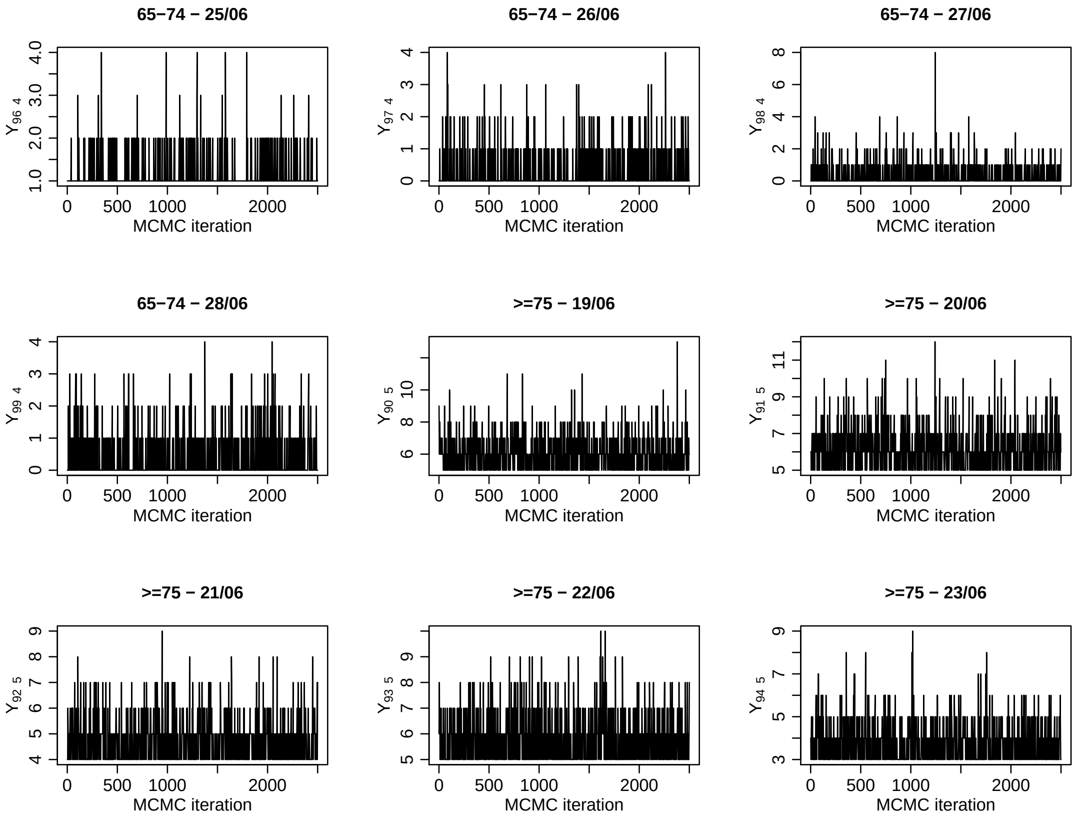

Figure S96: Trace plots for Y_{st} from analysis of London data

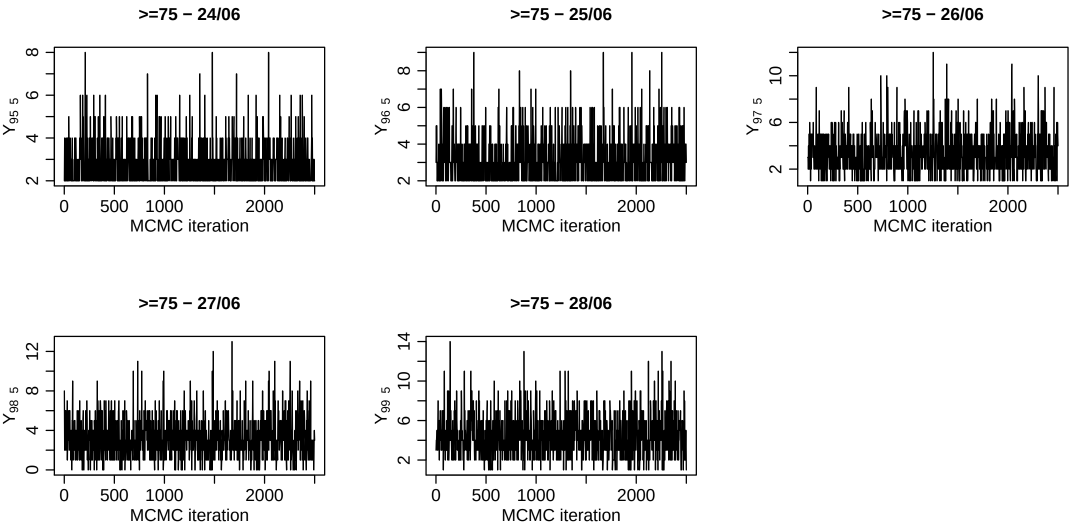

Figure S97: Trace plots for Y_{st} from analysis of London data
